# DeCoST: A New Approach in Drug Repurposing From Control System Theory

**DOI:** 10.3389/fphar.2018.00583

**Published:** 2018-06-05

**Authors:** Thanh M. Nguyen, Syed A. Muhammad, Sara Ibrahim, Lin Ma, Jinlei Guo, Baogang Bai, Bixin Zeng

**Affiliations:** ^1^Department of Computer and Information Science, Indiana University-Purdue University Indianapolis, Indianapolis, IN, United States; ^2^Institute of Molecular Biology and Biotechnology, Bahauddin Zakariya University, Multan, Pakistan; ^3^Department of Biology, School of Science, Indiana University-Purdue University Indianapolis, Indianapolis, IN, United States; ^4^The 1st School of Medicine and School of Information and Engineering, Wenzhou Medical University, Zhejiang, China; ^5^Institute of Lasers and Biomedical Photonics, Wenzhou Medical University, Wenzhou, China

**Keywords:** drug repurposing, system control, breast cancer, bladder cancer, pathway, expression profile

## Abstract

In this paper, we propose DeCoST (Drug Repurposing from Control System Theory) framework to apply control system paradigm for drug repurposing purpose. Drug repurposing has become one of the most active areas in pharmacology since the last decade. Compared to traditional drug development, drug repurposing may provide more systematic and significantly less expensive approaches in discovering new treatments for complex diseases. Although drug repurposing techniques rapidly evolve from “one: disease-gene-drug” to “multi: gene, dru” and from “lazy guilt-by-association” to “systematic model-based pattern matching,” mathematical system and control paradigm has not been widely applied to model the system biology connectivity among drugs, genes, and diseases. In this paradigm, our DeCoST framework, which is among the earliest approaches in drug repurposing with control theory paradigm, applies biological and pharmaceutical knowledge to quantify rich connective data sources among drugs, genes, and diseases to construct disease-specific mathematical model. We use linear–quadratic regulator control technique to assess the therapeutic effect of a drug in disease-specific treatment. DeCoST framework could classify between FDA-approved drugs and rejected/withdrawn drug, which is the foundation to apply DeCoST in recommending potentially new treatment. Applying DeCoST in Breast Cancer and Bladder Cancer, we reprofiled 8 promising candidate drugs for Breast Cancer ER+ (Erbitux, Flutamide, etc.), 2 drugs for Breast Cancer ER- (Daunorubicin and Donepezil) and 10 drugs for Bladder Cancer repurposing (Zafirlukast, Tenofovir, etc.).

## Introduction

Drug repurposing (also called drug repositioning) has become one of the most active areas in pharmacology since last decade (Oprea et al., 2011) because this approach could significantly reduce the cost and time to invent a new treatment. Before drug repurposing research became active, it was expected to take about 15 years and $0.8–$1 billion to bring a new drug into the market (Dimasi, 2001) due to many tests and clinical trials in order to be commercially approved by Food and Drug Administration (FDA) (USFDA, 2016). It is expected that the failure probability during clinical trials is about 91.4% (Thomas et al., 2016). One of the key reasons for low productivity in traditional drug development is the lack of systematic evaluation of additional indications (Dudley et al., 2011), which may lead to unexpected side effects and low efficacy. Briefly, drug repurposing finds new indications for known drugs and compounds (Gupta et al., 2013) to reduce the risk of failure and shorten time of discovery. Drug repurposing applies modern computational techniques to digitalize genomic (Power et al., 2014), bioinformatics, chemical informatics (Bisson, 2012) and patients' individual health records (Xu et al., 2014) to offer more systematic evaluation of the chemical compound before entering the laboratory testing and clinical trial steps. In addition, drug repurposing could explore the large set of chemical compounds, which is estimated to be more than 90 million by PubChem statistics (Wang et al., 2014), to reduce the cost of synthesizing new compounds. Prominent successful examples for drug repurposing include Viagra, Avastin, and Rituxan (Dudley et al., 2011).

**Figure 1:**
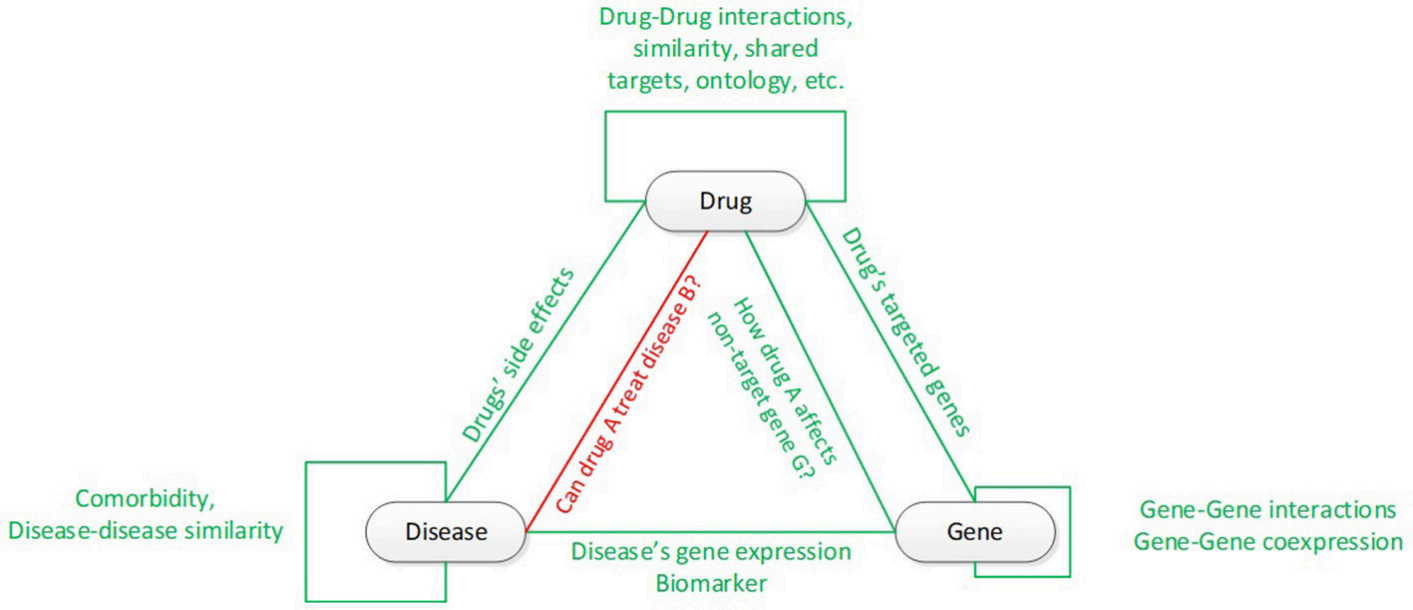
Connectivity among drugs, genes, and diseases. The red line and text show the key connectivity in drug repurposing.

System biology (Pujol et al., 2010) plays an important role to in the evolvement of drug repurposing evolved from “one: disease-gene-drug” (Durrant et al., 2010) to “multi: gene, drug” (Chou, 2010; Medina-Franco et al., 2013) and from “lazy guilt-by-association” (Campillos et al., 2008; Keiser et al., 2009; Iorio et al., 2010; Gottlieb et al., 2011) to “systematic model-based pattern matching,” such as the Broad Institute's Connectivity Maps (CMAP), C2MAP, etc. (Lamb et al., 2006; Hu and Agarwal, 2009; Huang et al., 2012; Jensen et al., 2012; Li and Lu, 2013; Subramanian et al., 2017). System biology reveals connectivity among drug, gene, and diseases (Figure 1). In this Figure, the green connectivity shows the types of connectivity for which drug repurposing could utilize to answer the key question: could drug A be re-indicated to treat disease B. The literature and public data sources for these types of connectivity have been thoroughly developed in the recent two decades, such as DrugBank (Law et al., 2013) and SFINX (Andersson et al., 2015) for drug-drug interaction; DrugBank (Law et al., 2013) and STITCH (Kuhn et al., 2012) for drug-gene/protein interaction; BioGRID (Chatr-Aryamontri et al., 2013), STRING (Szklarczyk et al., 2015), HAPPI (Chen et al., 2017), KEGG (Kanehisa et al., 2017) and Reactome (Croft et al., 2011) for protein-protein interaction and human pathway; OMIM (Baxevanis, 2012) and GEO (Barrett et al., 2013) for disease-specific gene curation and analysis; the human disease network (Goh et al., 2007) for disease-disease connectivity; and SIDER for diseases' drug-side-effect (Kuhn et al., 2016). The integration of rich data sources enable mathematical system modeling and analysis in system biology to deepen our understanding and predictive capability for biological processes, disease ontology (Hannon and Ruth, 2014; Goel and Richter-Dyn, 2016; Woodhead et al., 2016) and personalized medicine (Weston and Hood, 2004).

From the mathematical system-model-control-based point of view, there exist a mechanism regulating the gene expression profile. In the healthy condition, the gene expression stays in the stable equilibrium region such that **x**(*t*) = *f* (**x**(*t*−1)) ≈ **x**(*t*−1), where *f* indicates the expression-regulating mechanism computed from data integration, **x** stands for expression and *t* stands for time. In the disease state, the critical gene expression strays outside the stable region. In this case, without a control (treatment), the expression will be unbounded. The system control algorithms aim to find the sequence of control-treatment that optimally stabilize the expression back to the original equilibrium point, such as linear control (Willems, 1971; Chen et al., 2016), nonlinear control (Bardi and Capuzzo-Dolcetta, 2008; Falcone and Ferretti, 2013), adaptive neural network (Rovithakis and Christodoulou, 1994; Tong et al., 2014). By comparing the real drug treatments with the optimal control-treatment (also called hypo-treatment), we can evaluate the potential efficacy of the drug before being repurposed.

However, applying mathematical system modeling and control in drug repurposing is still in very early steps. There are three key challenges in applying system control approach. First, it is difficult to quantify the gene expression and real drug treatment, as there is very little literature discussing the “normal range” of each gene's expression. Second, constructing a comprehensive and accurate mathematical model to simulate the gene expression change is complicated due to the diversity of gene-gene interaction mechanisms, mutation, and under-discovered data. Third, the biological systems are known for large scale for system control: there may be from hundreds to thousands of genes of interest in a specific disease or biological process.

In this paper, we propose DeCoST (Drug Repurposing from Control System Theory) to apply control system paradigm for drug repurposing purpose, with source code available at https://github.com/thamnguy/DeCoST. The DeCoST framework tackles these challenges above as follow. First, although we could not completely solve the “normal range” challenge, we discretized the gene expression and the connectivity data so that the control-system algorithm could be executed logically without the “normal range” impact. Second, to overcome the comprehensiveness challenge, we utilized the biological and pharmaceutical knowledge and public data sources to quantify the drug-protein interaction and disease-specific gene expression profile. We used the comprehensive public protein-protein databases to setup the mathematical model for the repurposing problem. Third, to reduce the complexity and high-dimensionality of the repurposing problem, we applied the linear-quadratic-regulator method, which is practical in large-scale system control, to compute the hypo-treatment and evaluate the drug therapy. We apply DeCoST in Breast Cancer and Bladder Cancer case studies. Among cancer diseases, Breast Cancer causes the most number of mortality women (Centers for Disease Control Prevention, 2013). Breast Cancer is also the most comprehensively studied disease among cancers, with nearly 20 approved drugs by Food and Drug Administration (FDA). In addition, Breast Cancer has many subtypes, which is ideal for personalized drug repurposing. In contrast, FDA only approves 4 drugs for Bladder Cancer treatment although Bladder Cancer is the fourth most commonly diagnosed cancer in the United States (American Cancer Society, 2017). Therefore, drug development in Bladder Cancer is still an opened and attractive research area. From good performance when classifying between approved drugs and withdrawn drugs, we find 7 compounds that may be promising in Breast Cancer ER-positive subtype, 3 compounds in Breast Cancer ER-negative subtype and 10 compounds in Bladder Cancer for further drug repurposing *in-vivo* study.

## Methods

**Figure 2:**
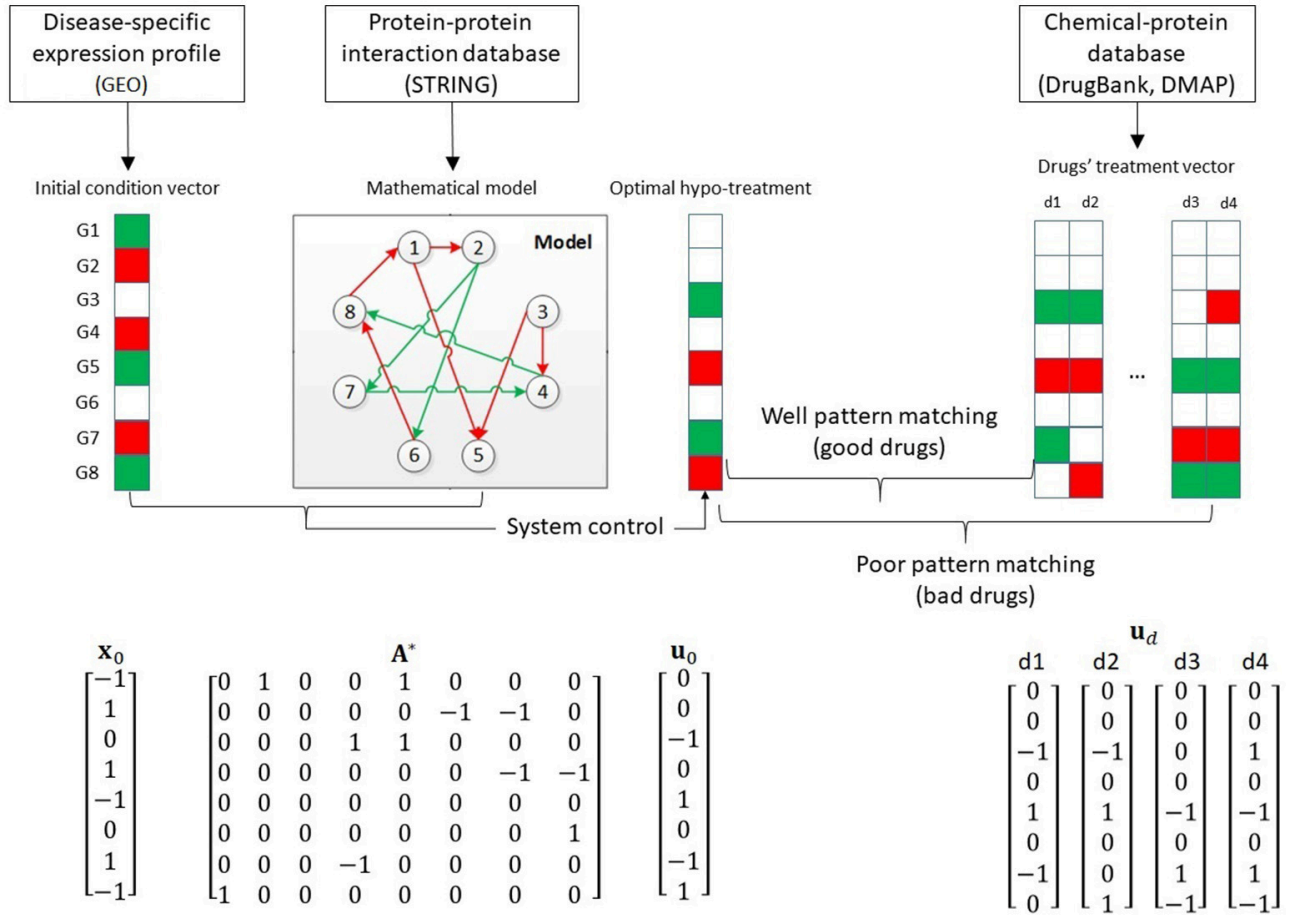
Overview of our drug repurposing framework and mathematical representation of drug, protein and interactome data. Red squares: overexpressed genes/drug's activation. Green squares: under expressed genes/drug's inhibition. Red arrow: activated protein-protein interaction. Green arrows: inhibited protein-protein interaction.

We developed our drug repurposing framework from the system modeling and control points (Figure 2). The framework integrates three types of data. First, from the Disease-specific expression profile, we quantified the expression as the system initial condition vector, where each vector elements specified whether the corresponding gene was overexpressed (red), underexpressed (green) or normally expressed (white). Second, from the protein-protein interaction database, we built the mathematical system model in order to apply the system-control algorithm. The red arrows implies activative; and the green arrow implies inhibitive interactions. Third, from the chemical-protein interaction data, we quantified the treatment vector for each drug for later ranking. Using the initial condition vector and the mathematical model, we computed the optimal hypo-treatment. By mapping the pattern of the optimal hypo-treatment and the drugs' treatment vectors, we could rank the drugs and suggest repurposed drugs.

### Retrieve the expression profile as the initial condition vector

We used GEO2R service (https://www.ncbi.nlm.nih.gov/geo/geo2r/) to analyze GEO dataset for the initial condition vector. The GEO2R service runs on R 3.2.3 platform and utilizes the well-known bioinformatics packages Biobase 2.30.0 (Huber et al., 2015), GEOquery 2.40.0 (Davis and Meltzer, 2007), and limma 3.26.8 (Ritchie et al., 2015). In GEO2R's result, we filtered out genes whose adjusted *p*-values exceed 0.05. The filtered-out genes were marked with 0 in the initial condition vector. For genes, whose adjusted *p*-values are less than 0.05, we used the sign of base-10 logarithm fold-change (logFC) in the initial condition vector. In the other words, genes with logFC > 0, which implied that the genes were overexpressed in the disease condition, were marked by 1. Genes with logFC < 0, which implied that the gene were under expressed in the disease condition, were marked by −1.

We chose GSE10886 dataset for expression profile in Breast Cancer case study. GSE10886 is among the largest and most comprehensive Breast Cancer microarray in GEO at the tissue level. After the latest update in January 2013, GSE10886 has 226 samples and including 97 ER-positive-subtype samples, 69 ER-negative-subtype samples, and 32 control samples. We chose GSE31189 dataset for Bladder Cancer expression profile. This dataset contains 52 cancer samples and 40 control samples.

### Build disease-specific mathematical system model from interactome data

Due to the availability of public data sources for disease-specific pathway models, we built the disease-specific system model for Breast and Bladder Cancer differently. To avoid potential false-positive, which is a well-known issue in predictive data source, we preferred using the pathway data to construct the mathematical model. For Breast Cancer, we conducted literature search on public curated pathway databases Reactome (Croft et al., 2011) and Wikipathway (Pico et al., 2008) for human disease pathways. In these databases, we only select pathways where the disease name appears in the pathways' titles or description. As the result, we found the Integrated Breast Cancer Pathway (Ibrahim et al., 2015) on Wikipathway. This pathway is among the most comprehensive Breast Cancer human pathway in the literature, which covers 239 genes and 467 interactions. The pathway also integrates 24 Breast Cancer-related pathways, including several signaling network. The entire detail about this pathway could be found in Supplemental Table 2. However, we could not find any pathways having more than 50 genes for Bladder Cancer, which implied low coverage. Therefore, for the Bladder Cancer model, we queried Bladder-Cancer-associated genes from PubMed Gene (https://www.ncbi.nlm.nih.gov/gene), one of the most comprehensive literature collection in biomedical and life sciences. To filter the possible noise during the retrieval process, we used specific query in format: <Disease Name> AND “Homo sapiens”[porgn: __txid9606]. After retrieving the Bladder-Cancer-associated genes, we converted the gene identification to UniProt Knowledge Base Reviewed identification (UniProt, 2013) to filter possible alias. We queried the STRING database v10 (Szklarczyk et al., 2015), one of the most comprehensive interactome databases to retrieve the interactions information among the candidate disease-specific proteins, especially the directionality and mechanism of interactions. To filter out possible noisy information, we limited the search results only on interaction with minimum of 500 confidence score. STRING database covers 7 types of mechanism: activation, expression, inhibition, catalysis, ptmod, binding, reaction.

After retrieving the disease-associated genes and interactions from these models above, we quantified the interactome to finalize the mathematical systems for these diseases. Among the interactions, activation and inhibitions are the mechanisms with the clearest and the most unambiguous impact/directionality. Thus, we quantified the activation mechanisms by +1 and the inhibition mechanisms by −1. For the other mechanisms, we quantified them by the default value of 0. The results of this step could be represented by adjacency matrices, as showed in Supplemental Figure 1.

### Retrieve chemical-protein interaction for treatment vector

For each disease, we curated literature for two set of drugs. The positive set, denoted by D1, includes all drugs which are approved for treatment by Food and Drug Administration (FDA). The negative set, denoted by D2, includes drugs which are withdrawn from disease treatment, or withdrawn/terminated from disease-specific clinical trials due to toxic or inefficient issues. We query https://clinicaltrials.gov/ for clinical trials information. To avoid the complexity of multi-drug and multi-disease treatment, we ignored literature mentioning more than one drug/disease during curation. We also ignored the biotech drugs since this type of drug does not target the molecular level, therefore it is difficult to setup the treatment vector for biotech drugs. Table 1 summarizes the list of D1 and D2 drugs we curated for Breast Cancer and Bladder Cancer. For Breast Cancer, we found 16 D1 drugs and 7 D2 drugs. In addition, to examine the possible newly therapeutic drugs for Breast Cancer, we referred to 24 drug proposed by Huang et al. (2011) as D3, in which these drugs have been approved for some other diseases by never in trial for Breast Cancer. For Bladder Cancer, we found 3 D1 drugs and 2 D2 drugs. Since we could not find any repurposed drug list for Bladder Cancer in the literature, we selected all of the 421 FDA-approved drugs for non-Bladder-Cancer diseases, which have at least one drug-gene interaction with genes in Bladder Cancer model, as D3 for Bladder Cancer. The entire D3 drug lists for both Breast Cancer and Bladder Cancer could be found in Supplemental Table 1.

**Table 1:** Drug lists (D1 and D2) curated for Breast and Bladder cancer.

**Disease**	**Drugs**	**Drug sets**	**Disease**	**Drugs**	**Drug sets**
Breast cancer	Anastrozole	D1	Breast cancer	Trastuzumab	D1
Breast cancer	Cycloheximide	D1	Breast cancer	Vinblastine	D1
Breast cancer	Exemestane	D1	Breast cancer	Diethylstilbestrol	D2
Breast cancer	Fluorouracil	D1	Breast cancer	Dromostanolone	D2
Breast cancer	Fluoxymesterone	D1	Breast cancer	Formestane	D2
Breast cancer	Fulvestrant	D1	Breast cancer	Ixabepilone	D2
Breast cancer	Lapatinib	D1	Breast cancer	Avastin	D2
Breast cancer	Letrozole	D1	Breast cancer	Ethyl Carbamate	D2
Breast cancer	Miltefosine	D1	Breast cancer	Imetelstat	D2
Breast cancer	Paclitaxel	D1	Breast cancer	Tivozanib	D2
Breast cancer	Pamidronate	D1	Bladder cancer	Cisplatin	D1
Breast cancer	Raloxifene	D1	Bladder cancer	Doxorubicin HCl	D1
Breast cancer	Tamoxifen	D1	Bladder cancer	Thiotepa	D1
Breast cancer	Thiotepa	D1	Bladder cancer	Mitomycin C	D2
Bladder cancer	Gemcitabine	D2			

*D1, FDA-approved drugs (positive/good drug set); D2, FDA-rejected/withdrawn drugs (negative/bad drug set)*.

We queried the DrugBank (Law et al., 2013) and DMAP (Huang et al., 2015) database for the list of drug-protein interaction mechanism. DMAP and DrugBank covers 38 mechanisms of drug action. In DMAP, we filtered out interactions with confidence score less than 800 (over 1,000) to avoid noisy information. From biological knowledge, we quantified these mechanisms as showed in Table 2. Similar to quantification of protein-protein mechanism of action, an inhibited or similar action is map to −1; and an activated or similar action is map to +1.

**Table 2:** Quantification of drug-protein mechanism of action in drug-protein interaction databases.

**Mechanism of action**	**Quantification**	**Mechanism of action**	**Quantification**
Activator	1	Ligand	0
Adduct	0.5	Metabolizer	0
Agonist	1	Modulator	0
Allosteric modulator	0	Multitarget	0
Antagonist	−1	Negative modulator	−1
Antibody	0	Neutralizer	0
Binder	0	Other	0
Chaperone	1	Other/unknown	0
Chelator	0	Partial agonist	1
Cleavage	−1	Partial antagonist	−1
Cofactor	1	Positive allosteric modulator	1
Component of	0	Potentiator	1
Cross-linking/alkylation	0	Product of	0
Incorporation into and destabilization	−1	Reducer	−1
Inducer	1	Stimulator	1
Inhibitor	−1	Suppressor	−1
Inhibitor, competitive	−1	Unknown	0
Inhibitory allosteric modulator	−1	Other terms	0
Intercalation	0	–	–

*The Mechanism of Action terminologies are retrieved from drug-target annotation in DrugBank database. Quantification stands for the numerical representation of the Mechanism of Action in the modeling and computing steps*.

### Construct disease-specific drugs' therapeutic scoring for drug repurposing purpose

The key principle in applying system control to evaluate drugs' therapy relies in the following assumption: in disease condition, the gene expressions are derived away from the balanced level of 0. Therefore, a good treatment should reverse the gene expressions in disease condition and stabilize the expressions to the balance level. In Figure 2, we illustrate this principle and explain several mathematical notation in a toy example. Based on system biology literature (Alberghina, 2007), we assume that there exists a model governing the gene expressions, which allows us to model the expression using time-series perspective

(1) x(t)=f(x(t−1),u(t−1))

where **x** ϵ ℜ^*N*^ stands for the quantified gene expression of *N* genes, **u** ϵ ℜ^*N*^ stands for the quantified treatment and *t* is the iteration and *f* is the arbitrary function controlling the expression change. The initial **x**(0) is the quantified gene expression in disease condition. In this paper, we choose a linear model for *f*.

(2) x(t)=Ax(t−1)+u(t−1)

We chose the linear model because not only it is simple but also it has equilibrium point at the origin: if **x**(*t*−1) = **u**(*t*−1) = 0 then **x**(*t*) = 0. This fact implies that when the gene expressions are already at the balance level, treatment is no longer needed. In addition, it is easier to setup a linear system with stability (Chui and Chen, 2012)

(3) If||x(0)||<ε and u=0 then || x(t) ||<ε∀t

where ||**x**|| stands for the second norm of **x** and ε is an arbitrary small number. This fact implies the self-adjustment of the gene expression at the control level. We setup matrix **A** from quantification of protein-protein mechanism of interactions (section Methods). With temporal matrix A^*^ as the result of section Methods

(4) $$A^{*}(i,j)\;=\;\{\begin{array}{c} -1\text{ if protein }i\text{ inhibits protein }j\\ 1\text{ if protein }i\text{ activates protein }j\\ 0\text{ otherwise} \end{array}$$

Let λ be the eigenvalue of A^*^ with the largest magnitude. By setting up **A** as

(5) $$A=(1/\lambda )\;A^{*}$$

We can guarantee the stability of system (2) (Chui and Chen, 2012).

The objective of the linear control is to find a sequence of **u**(t) such that

(6) x(t)→0 as t→∞

Optimal control considers not only how to stabilize **x** quickly but also consider the cost-effective of the treatment **u**. Regarding this point, the optimal linear control aims to minimize

(7) $$J(x\left(0\right))=\sum_{t\;=\;0}^{\infty }\left(x{\left(t\right)}^{T}x\left(t\right)+u{\left(t\right)}^{T}u\left(t\right)\right)$$

To solve the optimization problem (2–7) we solved the corresponding Riccati equation (Arnold and Laub, 1984)

(8) $$A^{T}PA-P-A^{T}P{\left(P+I\right)}^{-1}PA+I=0$$

using DARE algorithm (Arnold and Laub, 1984) in Matlab (https://www.mathworks.com/help/control/ref/dare.html). In (8), P is just an intermediate result containing no biological representation. We compute the treatment vector **u**(*t*) as follow

(9) $$u\left(t\right)=-{\left(I+P\right)}^{-1}PAx(t)$$

In system control practice, since **u**(*t*) often converges to 0 quickly (Bemporad et al., 2002), the first treatment vector **u**(0) = −(**I** + **P**)^−1^**PAx**(0) often plays the most important role in optimally stabilizing the system (2). Therefore, we can consider **u**(0) as the optimal hypo-treatment. We compare the similarity between the real drug treatment (**u**_*d*_) and the hypo-treatment as the therapeutic score *T*(*d*) for each drug *d* as follow

(10) $$T_{d}=|u_{d}^{T}\text{sign}(u\left(0\right))|/|\text{abs}{\left(u_{d}\right)}^{T}\text{abs}(\text{sign}(u\left(0\right)))|$$

where abs stand for the absolute value function. Here, *T*_*d*_ ranges between −1 and 1. The numerator $\left|{\text{u}}_{d}^{T}sign\left(\text{u}\left(0\right)\right)\right|$ is the matching function between drug *d* and the optimal hypo-treatment, which is incremented when **u**_*d*_(*i*) and **u**(0)(*i*) are non-zero analog, and decremented when **u**_*d*_(*i*) and **u**(0)(*i*) are opposite. We measured the impact of *T*_*d*_ score by the receiver operating characteristic when we use *T*_*d*_ to classify D1 drugs vs. D2 drugs.

## Results

### Therapeutic scores for breast cancer drugs

From the Integrated Breast Cancer Pathway (Ibrahim et al., 2015) on Wikipathway (section Methods) and the Breast Cancer drug list in Supplemental Table 3, we queried 222 drug-protein interactions for the drugs' treatment vectors (Supplemental Table 4). Supplemental Table 5 contains the initial condition vector from GEO2R expression analysis.

**Figure 3:**
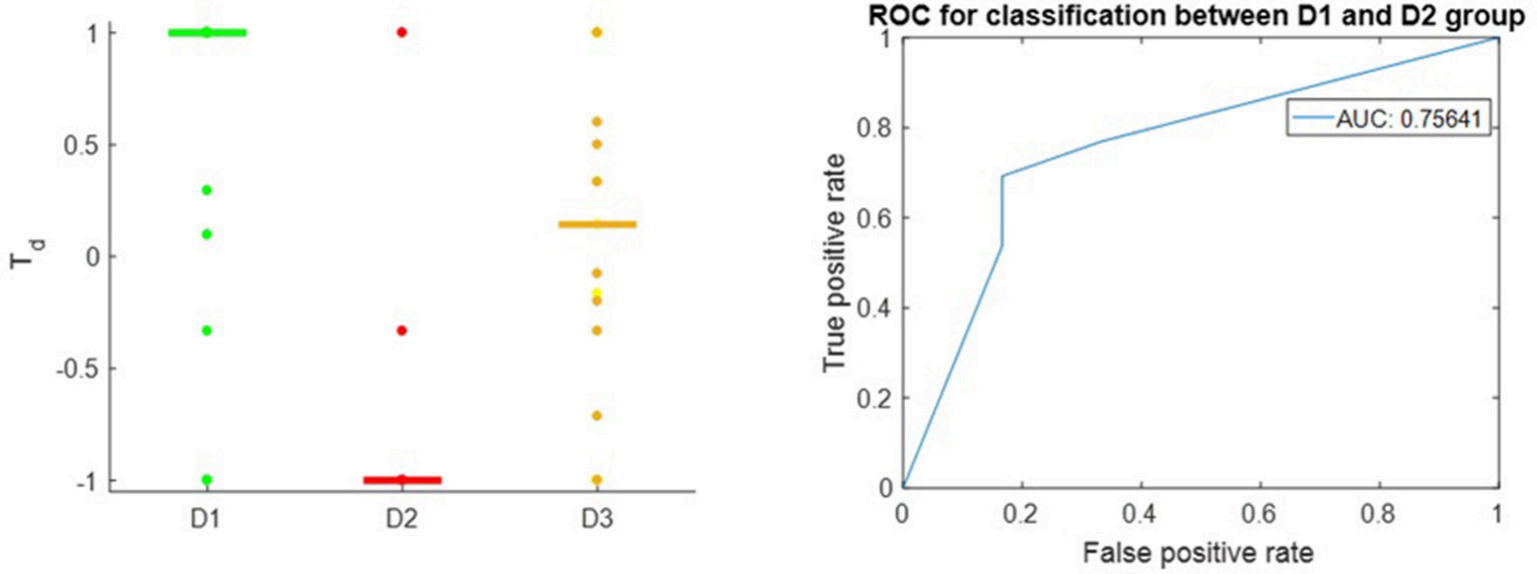
**Left**: *T*_*d*_ score in Breast Cancer, ER-positive subtype; the horizontal bars in each group stand for median value of *T*_*d*_. **Right**: ROC of *T*_*d*_ in classifying between D1 drugs and D2 drugs.

**Figure 4:**
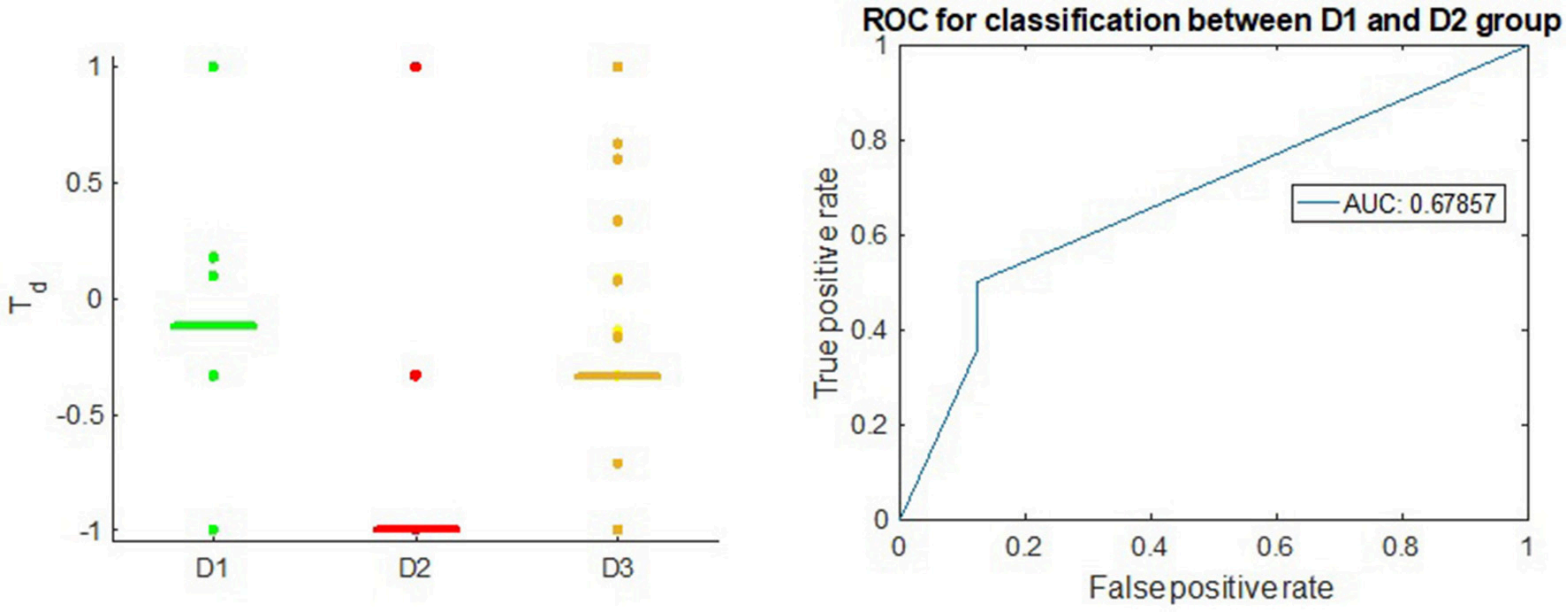
**Left**: T_d_ score in Breast Cancer, ER-negative subtype; the horizontal bars in each group stand for median value of T_d_. **Right**: ROC of T_d_ in classifying between D1 drugs and D2 drugs.

Figure 3 shows that the T_*d*_ score is able to give appropriate ranking for most of the well-known therapeutic drugs and suggest candidate drugs for repurposing in Breast Cancer ER-positive case. T_*d*_ score reflexes the difference between the D1 and D2 drugs with receiver operator characteristic (Hanley and McNeil, 1982) area under the curve (AUC) of 0.76. This result is comparable to the overall result queried from Broad Institute CMAP (Subramanian et al., 2017) on MCF-7, the Breast Cancer ER+ cell line, using the Touchstone tool (https://clue.io/touchstone). Especially on the drugs covered in CMAP, DeCoST achieves AUC of 0.91, which is much higher than the AUC achieved by CMAP (0.79), as showed in the Supplemental Text 1. We did not setup training set and test set for classification because the model construction and *T*_*d*_ calculation does not need the drug categories. The T_*d*_ scores for D1 drugs in Breast Cancer ER-negative case are relatively lower than the scores for ER-positive case (Figure 4). Comparison detail has been shown in Supplemental Table 5. Using T_*d*_ for classifying D1 and D2 drugs yields AUC of 0.68. In fact, clinical trials and literature have showed several drugs which are effective in ER-positive treatment but show little or no impact in ER-negative treatment. For example, Tamoxifen (*T*_*d*_ ER-positive: 0.294, *T*_*d*_ ER-negative: 0.176), which is a selective estrogen receptor modulator, does not prevent ER-negative Breast Cancer, when the estrogen receptor genes do not express (Fabian, 2007; Uray and Brown, 2011).

### Therapeutic scores for bladder cancer drugs

Since we could not find any human pathway with sufficient coverage for Bladder Cancer, our Bladder Cancer system model retrieved the Bladder-Cancer-specific genes from PubMed Gene server. The model contains 738 proteins and 1,241 protein-protein interactions. From 6 drugs in the Bladder Cancer case-study, we retrieved 48 drug-protein interactions for drugs' treatment vector. From GSE31189 gene expression dataset, we found 221 genes whose expression differs from the balance level. Details about the Bladder Cancer system could be found in Supplemental Tables 6–8.

**Figure 5:**
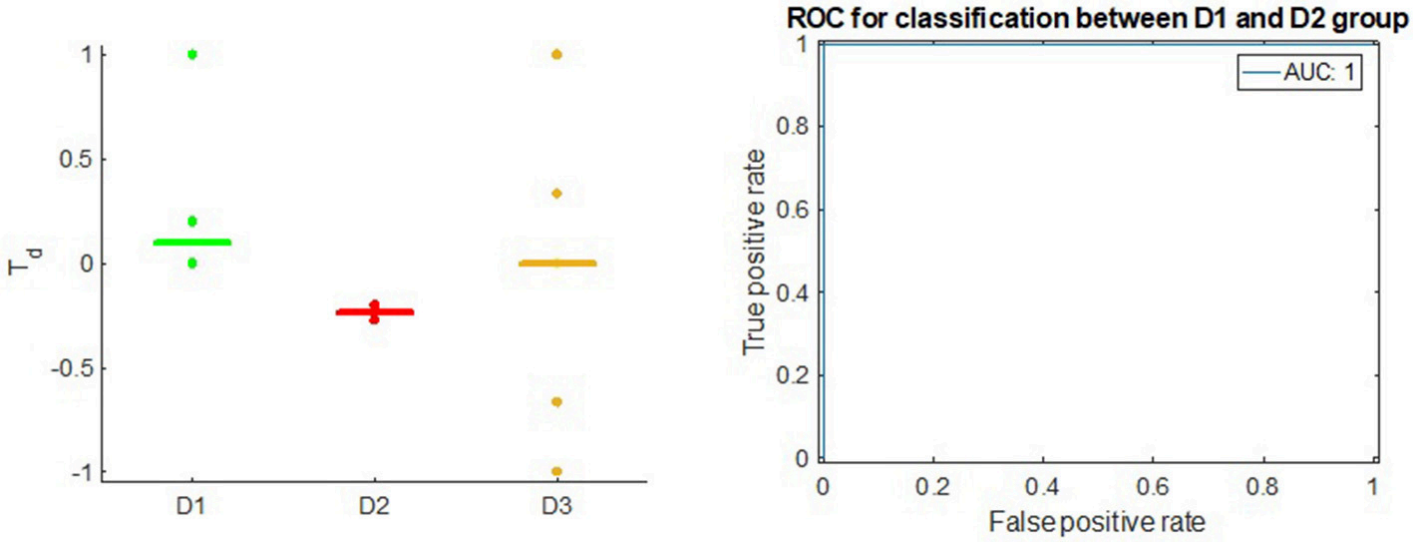
**Left**: *T*_*d*_ score in Bladder Cancer; the horizontal bars in each group stand for median value of *T*_*d*_. **Right**: ROC of *T*_*d*_ in classifying between D1 drugs and D2 drugs.

We observed AUC of 1.0 (Figure 5) when we used *T*_*d*_ score to classify between D1 and D2 drugs in Bladder Cancer. Here, all of the D1 drugs receive non-negative *T*_*d*_ scores: Cisplatin receives the score of 0.2, Doxorubicin Hydrochloride receives the score of 0.0 and Thiotepa receives the score of 1.0. All of the D2 drugs receive negative *T*_*d*_ scores: Mitomycin C receives the score of −0.2 and Gemcitabine receives the score of −0.09.

### Potential drugs for breast cancer studies and biological insights

**Figure 6:**
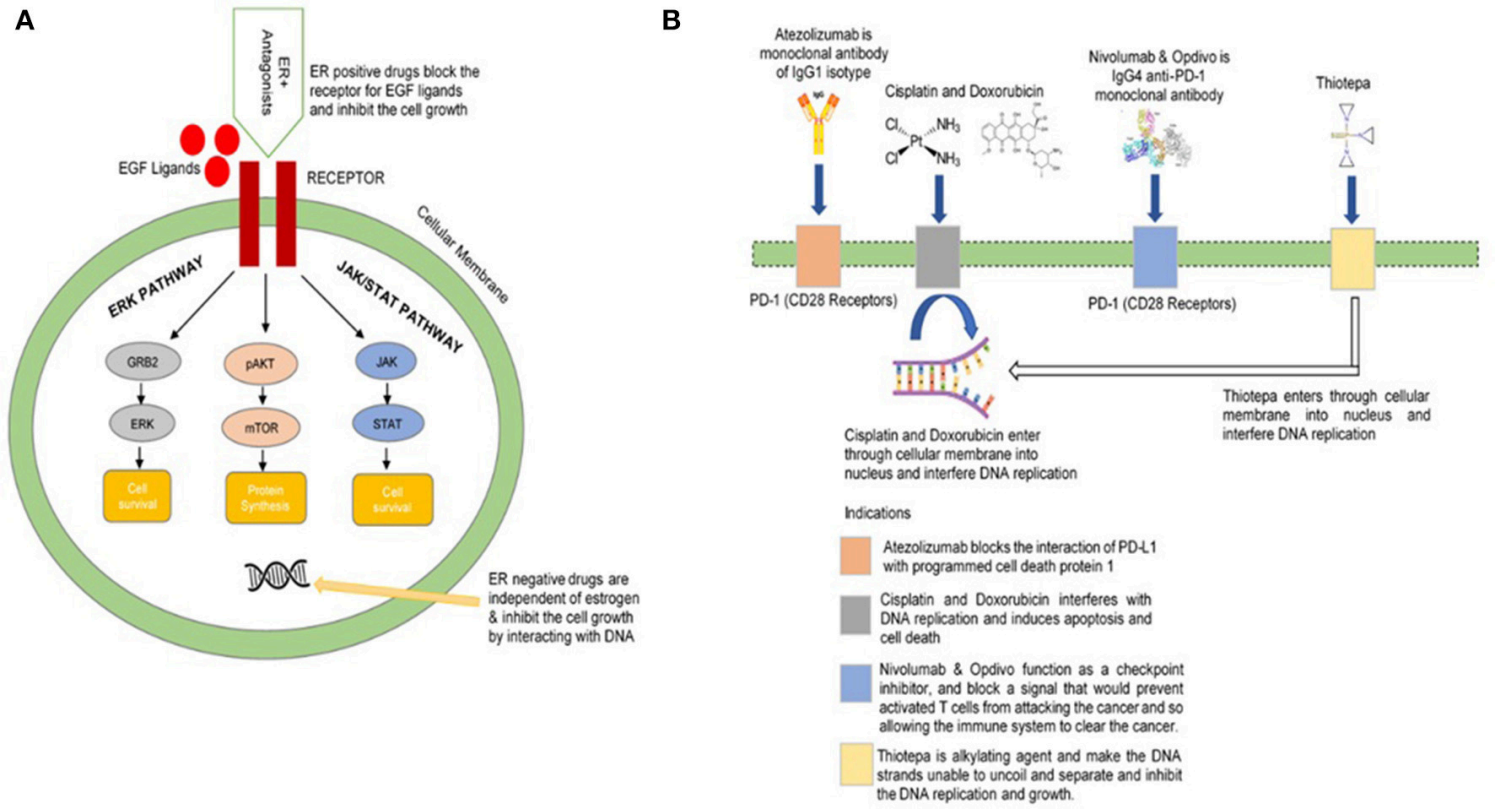
Illustration of biological mechanism of few FDA approved drugs **(A)** for breast cancer **(B)** for bladder cancer.

From the *T*_*d*_ scores for D3 drugs, our framework suggests 8 drugs (Erbitux, Flutamide, Medrysone, Methylprednisolone, Norethindrone, Prednisolone, Prednisonea, and Vandetanib) with high potential efficacy in Breast Cancer ER+ drug repurposing. Significantly, these drugs do not directly target Estrogen receptor, which is the most well-known approach in Breast Cancer ER+ drug design. Tamoxifen is a typical example of Breast Cancer drugs which slows cancer process by blocking estrogen hormone receptors, preventing hormones from binding to them. About 80% of all breast cancers are ER+: the cancer cells grow in response to the hormone estrogen (Bulut and Altundag, 2015). About 65% of the ER+ cases grow in response to another hormone, progesterone (Hefti et al., 2013). Tumors in ER/PR-positive cases are much more likely to respond to hormone therapy than tumors that are ER/PR-negative. ER+ breast cancer entirely depends on the estrogen for growth and propagation involving genomic and non-genomic pathways. Epidermal growth factor receptor (EGFR) is a receptor found on both normal and tumor cells that is important for cell growth (Herbst, 2004; Khoo et al., 2015). ER-positive (ER+) drugs recommended for repurposing in this framework block the activities and growth of EGFR (Figure 6A). These drugs show different mechanism of action with the common objective of the inhibition of the growth of cancerous cells. By adjusting and modifying the known biases of the interactomic networks, our procedure would help to reveal the therapeutic effect of drugs along with effective treatments.

For Breast Cancer ER- case, our framework suggests Daunorubicin and Donepezil as the repurposing candidates. These drugs are independent of estrogen and usually inhibit the cell growth by either interacting with DNA or inhibiting Cholinesterases. Daunorubicin interacts with DNA by intercalation and inhibition of macromolecular biosynthesis (Momparler et al., 1976). This inhibits the progression of the enzyme topoisomerase II, and thereby stopping the process of replication. Donepezil is in a class of cholinesterase inhibitor that improves mental function and fatigue in cancer. The current research focused on recent large-scale efforts to systematically find repositioning candidates and elucidate individual disease mechanisms in cancer (Bruera et al., 2007). Personalized medicine and repositioning both aim to improve the productivity of current drug discovery pipelines. Standard drug discovery strategies can also lead to repositioning opportunities. D1, D2, and D3 drugs (Table 1) found to potently modulate the desired activity are repositioning candidates.

### Potential drugs for bladder cancer studies and biological insights

From the list of 143 FDA-approved drug with high *T*_d_ score, we found 10 candidates drugs (with *T*_d_ = 1) whose mechanisms are promising for Bladder Cancer repurposing. The *T*_d_ scores for all Bladder Cancer drugs could be found in Supplemental Table 9. The prevalence of drug-repositioning studies has resulted in a variety of innovative computational methods for the identification of new opportunities for the use of old drugs. We sorted the potential list of drugs against bladder cancer. The reprofiling of these drugs followed the same biological mechanisms. For example, Zafirlukast antagonizes ATP-binding cassette and may improve the efficacy of anticancer effects (Sun et al., 2012). Similarly, Tenofovir may reduce the risk of bladder or others cancers while dopamine receptor antagonist Thioridazine inhibits tumor growth (Yin et al., 2015). Losartan is an angiotensin II receptor (AT-II-R) blocker that is widely used by human for blood pressure regulation but it also shows antitumor property (Barreras and Gurk-Turner, 2003). Ciclopirox was first marketed in 1982 as an antifungal agent found in several topical drug products. However, further research demonstrated that it was able to kill bladder cancer cells (Weir et al., 2011). The Atezolizumab, Cisplatin, Doxorubicin, Nivolumab, Opdivo, Thiotepa, and others (Figure 6B) are FDA approved drugs which are recommended for bladder cancer.

## Discussion

The applications of drug-repositioning studies have brought a variety of new *in silico* approaches in drug designing and development. In most of the studies, the anticancer effect of newly designed drugs usually has been presented *in vitro* as clinical trials are very expensive and time consuming, but remain the only way to validate drug efficiency *in vivo*. Therefore, to establish accurate and effective drug-repositioning framework needs development of new computational techniques. In this work, we discuss and demonstrate the application of control system theory as a computational method to evaluate drug efficacy and repurposing from integrated system biology data. The capability in classification between approved and withdrawn drugs is the fundamental foundation for our framework in drug repurposing. It is important to note that although our AUC of 0.76 and 0.68 in Breast Cancer is inferior compared to the state-of-the-art methods (Cheng et al., 2012; Zheng et al., 2015), our validation is conducted from the pharmaceutical knowledge of drug's efficacy on treatment at the system-pathway level; meanwhile, the other methods often validate at the targeted molecular level. In addition, we set strict criteria in choosing the negative set by only choosing drugs that are rejected or withdrawn from disease-specific clinical trials and treatments. The state-of-the-art methods tend to be more relaxed on the negative set by choosing drug not being used in disease-specific drugs, which may have limitation on repurposing options. In addition, the appropriate assessment of tamoxifen efficacy between Breast Cancer ER+ and Breast Cancer ER- highlights the potential advantages of our framework in personalized drug repurposing. Compare to the approved drugs, the candidate drugs suggested in this work show different promising drug mechanisms which may be useful in future drug design.

In our work, although the number of target may be among the key difference between the D1 drugs and the D2 drugs, our analysis shows that the number of drugs' targeted genes and the targeted genes are not the only factors affecting the clinical outcome and predictive results in drug repurposing. As showed in Suppemental Table 3, D1 drugs, on the average, has more targets than D2 drugs. However, D1 drugs for Breast Cancer (average number of targets: 4.8) include both single-target (such as Anastrozole, Exemestane, and Fluorouracil) and multi-target (such as Tamoxifen, Paclitaxel, and Cycloheximide) ones. D2 (average number of targets: 3.3) drugs also contains the single-target (such as Ixabepilone and Avastin) and the multi-target (such as Imetelstat and Diethylstilbestrol). In the result section, DeCoST's evaluation for these drugs showed above is appropriate for their clinical outcome. In addition, drugs targeting the same marker genes do not necessary have the same outcome. For example, both Tamoxifen and Diethylstilbestrol target the estrogen receptors ESR1 and ESR2, which are the marker in Breast Cancer ER+ (Yip and Rhodes, 2014). However, their clinical outcomes and DeCoST's evaluation are opposite, primarily because they have opposite mechanisms on the same targets of estrogen receptors: Tamoxifen is the estrogen inhibitor while Diethylstilbestrol is the estrogen activator. Since Breast Cancer ER+ is strongly associated with the overexpression of estrogen receptors (Yip and Rhodes, 2014), Tamoxifen could have therapeutic outcome because it reverses the disease signature. Meanwhile, Diethylstilbestrol should have poor outcome because it shows the analog to the disease signature.

In this work, we have showed the results between DeCoST and the Broad Institute CMAP, which is among the most well-known and comprehensive platforms for drug repurposing. In addition, our strategy of repurposing is similar to CMAP. Although Supplemental Text 1 shows that our DeCoST has higher AUC than CMAP does, it is inappropriate to conclude that DeCoST is better than the CMAP. There are fundamental differences in conducting experiment making comparison not totally solid. First, the expression profiles acquired by CMAP are at the cell line level; meanwhile, in this work DeCoST acquires the expression profile at the tissue level, which is closer to *in-vivo* studies. Second, due to several factors in experimental design, CMAP does not contains cell line for Breast Cancer ER- and Bladder Cancer. CMAP also covered less number of drugs, compared to the drug list evaluated in this work. Therefore, the key point in comparative evaluation should be on the repurposing hypotheses suggested by these platforms in future *in-vivo* studies and the biological insights of these hypotheses. In our results, we have offered several biological explanations why drugs recommended by DeCoST could be repurposed. Unfortunately, we could not compare between CMAP and DeCoST at this point. DeCoST focuses primarily on recommending drugs that have never been in disease-specific clinical trials; meanwhile, CMAP (https://clue.io/repurposing-app) primarily reports on drugs that has been under early phases of clinical trials. Therefore, we believe that DeCoST could provide complimentary advantages, in addition to CMAP.

The advantages of our framework are established not only by advanced computational method but also by two layers of personalized system (Li and Jones, 2012). In the first layer, the disease-specific gene expression could differ among different patients and subtypes, which results in different initial state condition. In the second layer, different types of disturbance among molecular-molecular interactions could be discovered and represented differently in the system modeling step. In our results, we show that Tamoxifen, which is approved to treat Breast Cancer, may not be effective in treating Breast Cancer ER-. The strong support from literature to this evaluation is a good example of the personalized medicine characteristics. In addition, our framework could easily integrate the results from many other state-of-the-art repurposing approaches such as molecular docking and gene-set enrichment analysis to refine the efficacy prediction. The main idea in this framework, which is based on control system theory, could be applied in many other bioinformatics problem, such as target prioritization and discovering new combination of treatments. In addition, our framework could easily be extended to evaluate combination of treatment, with careful preprocessing the drug-drug interaction data (Ayvaz et al., 2015; Wang et al., 2017).

In addition, our framework shows repurposing capacity at both target level and pathway level. At the target level, we show typical examples for EGFR-targeted and ACHE-targeted drugs. Patients being considered for anti-epidermal EGFR therapy are often screened for mutations in the oncogene KRAS (Hoorens et al., 2010) because a constitutively active KRAS gene downstream of EGFR would not be affected by EGFR inhibition. Many diseases have approved combination regimens, such as metastatic colorectal and bladder cancer and its four-drug FOLFIRI (folinic acid, 5-fluorouracil, irinotecan) with cetuximab regimen (Raoul et al., 2009). Losartan is an angiotensin II receptor (AT-II-R) blocker and this angiotensin-converting enzyme inhibitors (ACE) may have a protective role in bladder and other cancers (Yazdannejat et al., 2016). In the other hand, a typical example at the pathway level is Thioridazine. Thioridazine-induced effects are associated with inhibition of the canonical NFκB pathway.

The limitations in this work are the method to quantify the categorical data from public genomic/proteomic databases and the simplicity of linear system control. First, all of the data are discretized into only three values: −1, 0, and 1, which could lower the resolution of the final drug therapeutic score. Second, the linear system control approach needs to assume that the gene expression transition could be approximate closely by a linear equation, which is still unverified due to the scarcity of time-series gene expression data. Therefore, when applying into another repurposing problem, biologists and pharmacologists should apply deeper domain knowledge to increase the resolution of discrete quantification. Furthermore, mathematical nonlinear system identification and reinforcement learning, which are popular approach in unknown system control, could be used to increase the accuracy of system modeling and make the system more personalized. Integration of other resources, such as drugs, genes, and systems associated with side-effects (Kuhn et al., 2016; Maier et al., 2018) and high-throughput screening (Deftereos et al., 2011; Macarron et al., 2011) would also be valuable expansions of this work in the future. Also, the computational complexity of DeCoST is generally high (expected O(*n*^8^), where *n* is the number of genes in the model). This complexity is manageable with most of the existing biological pathway model (expect about 400 genes). However, this could be a bottleneck if the number of genes raises to several thousands.

In addition, the advantages of our framework in personalized medicine may associate with the reproducibility issues (Draghici et al., 2006; Frye et al., 2015). As mentioned, the disease-specific gene expression could differ among different patients and subtypes. Therefore, we could not completely guarantee that applying our framework on different gene expression data and on different interactome data sources (Chatr-Aryamontri et al., 2013; Szklarczyk et al., 2015) would return the same result. Therefore, by reproducibility, we can only guarantee that given a specific gene expression profile and an interactome data source, we can always produce the same result. In this work, we have tried to tackle the reproducibility issue by using tight criteria to select the positive/negative drug set, by maintaining the relevance and coverage of the disease-specific model, and by choosing the expression data set with high number of samples.

## Conclusion

In this work, we have developed DeCoST, one of the first techniques from system control paradigm, to tackle the drug repurposing challenges. We showed that DeCoST could appropriately retrieve the clinical outcomes of drugs treating personalized Breast Cancer and Bladder Cancer. From the good retrieval result, DeCoST suggests repurposing 8-candidate drugs for Breast and 10 drugs for Bladder Cancer with biological insights. This framework would be promising to discover new therapeutic strategies to treat other cancer diseases.

## Author contributions

TN designed the study (including the mathematical details), curated the Bladder Cancer drug dataset and analyzed the computational results. SM validated and provided biological insights for the results. SI constructed the Breast Cancer pathway model and collected the drug clinical outcomes for Breast Cancer. LM processed the expression and protein-protein interaction data for Bladder Cancer. LM, JG, BB, and BZ implemented the system control algorithm used in the paper. All authors contributed to the manuscript writing and edition.

### Conflict of interest statement

The authors declare that the research was conducted in the absence of any commercial or financial relationships that could be construed as a potential conflict of interest. The reviewer YW and handling Editor declared their shared affiliation.
