# Supplementary material for: DeCoST: A New Approach in Drug Repurposing From Control System Theory
Source: Front Pharmacol. 2018 Jun 5;9:583. doi: 10.3389/fphar.2018.00583 (PMC5996185; doi:10.3389/fphar.2018.00583)
Supplement: Supplementary file 1 [file Data_Sheet_1.DOCX]

## Supplemental Text 1: Breast Cancer repurposing using CMAP

In this section, to give a fair assessment on the quality of DeCosT in recommending drugs for repurposing, we queries the Connectivity Score between the drug signature and the disease-specific cell line signature from Broad Institute’s Connectivity Map (CMAP) (Subramanian, Narayan et al. 2017). CMAP (Lamb 2007) is among the most well-known and comprehensive platforms for drug repurposing. In addition, our strategy of repurposing is similar to CMAP. CMAP hypothesizes that on a disease-specific cell line, a good drug should have treatment signature opposite to the non-treatement signature (Subramanian, Tamayo et al. 2005, Lamb 2007). The CMAP queries are conducted from CMAP web service at [https://clue.io/](https://clue.io/connection?url=data.clue.io/tsv2/digests/BRD-K04210847) using Touchstone tool and MCF-7 cell line. In this service, given a specific cell line, drugs having higher connectivity score (range between -100 and 100) show more opposite signature compared to the non-treatment signature and are hypothesizes to be more therapeutic.

Due to some reasons in experimental design, at this point, CMAP only contains the MCF-7 cell line (Fornari, Randolph et al. 1994), which is well-known for Breast Cancer ER+ testing, that matches to some DeCoST result. CMAP does not contain any cell line for Breast Cancer ER- / Bladder Cancer. In addition, we could only query 14/23 drugs tested by DeCoST (Table 1), as showed in the Supplemental Table T1. On these drugs, CMAP shows the AUC of 0.79. Meanwhile, DeCoST shows the AUC of 0.91 (Supplemental Figure T1)

| Drug | Class | CMAP Score | DeCoST T*_d_* score |
| --- | --- | --- | --- |
| Anastrozole | D1 | 20.13 | 1 |
| Cycloheximide | D1 | 93.44 | 0.097 |
| Exemestane | D1 | 16.62 | 1 |
| Fulvestrant | D1 | 99.48 | 1 |
| Lapatinib | D1 | 30.13 | 0 |
| Letrozole | D1 | 30.82 | 1 |
| Paclitaxel | D1 | 24.16 | 1 |
| Raloxifene | D1 | 99.01 | 0 |
| Tamoxifen | D1 | 99.98 | 0.294 |
| Thiotepa | D1 | -32.16 | 1 |
| Vinblastine | D1 | 77.65 | 0 |
| Diethylstilbestrol | D2 | -85.51 | 0 |
| Formestane | D2 | 31.19 | 0 |
| Imetelstat | D2 | 0 | -0.333 |

Supplemental table T1: CMAP score and DeCoST T*_d_* score for drugs that could be queried on MCF-7 cell line.

Supplemental figure T1: CMAP and DeCoST AUC for the 13 drugs queriable from CMAP.

## Supplemental Figure 1

Summary about constructing disease-specific mathematical model from protein-protein interaction

## Supplemental table 1

D3 drug list

| Disease | Drug |
| --- | --- |
| Breast Cancer | Bleomycin |
| Breast Cancer | Corticosterone |
| Breast Cancer | Daunorubicin |
| Breast Cancer | Dexamethasone |
| Breast Cancer | Dihydrotestosterone |
| Breast Cancer | Donepezil |
| Breast Cancer | Erbitux |
| Breast Cancer | Ethinyl Estradiol |
| Breast Cancer | Flutamide |
| Breast Cancer | Hydrocortisone |
| Breast Cancer | Hydroxyurea |
| Breast Cancer | Lithium Chloride |
| Breast Cancer | Medrysone |
| Breast Cancer | Methyl Methanesulfonate |
| Breast Cancer | Methylprednisolone |
| Breast Cancer | Mitomycin |
| Breast Cancer | Nocodazole |
| Breast Cancer | Plicamycin |
| Breast Cancer | Prednisolone |
| Breast Cancer | Prednisone |
| Breast Cancer | Progesterone |
| Breast Cancer | Testosterone |
| Breast Cancer | Trilostane |
| Breast Cancer | Vandetanib |
| Bladder Cancer | 4-methylpyrazole |
| Bladder Cancer | 5-ASA |
| Bladder Cancer | 5-azacytidine |
| Bladder Cancer | 8-MOP |
| Bladder Cancer | 9-cis-retinoic acid |
| Bladder Cancer | abiraterone |
| Bladder Cancer | acebutolol |
| Bladder Cancer | acetaminophen |
| Bladder Cancer | acitretin |
| Bladder Cancer | adapalene |
| Bladder Cancer | adenosine monophosphate |
| Bladder Cancer | agomelatine |
| Bladder Cancer | ajmaline |
| Bladder Cancer | allylestrenol |
| Bladder Cancer | amcinonide |
| Bladder Cancer | AMD3100 |
| Bladder Cancer | amiloride |
| Bladder Cancer | aminoglutethimide |
| Bladder Cancer | amiodarone |
| Bladder Cancer | amitriptyline |
| Bladder Cancer | amlodipine |
| Bladder Cancer | amodiaquine |
| Bladder Cancer | amrinone |
| Bladder Cancer | amsacrine |
| Bladder Cancer | anastrozole |
| Bladder Cancer | arsenic trioxide |
| Bladder Cancer | aspartame |
| Bladder Cancer | aspirin |
| Bladder Cancer | atomoxetine |
| Bladder Cancer | atorvastatin |
| Bladder Cancer | atropine |
| Bladder Cancer | axitinib |
| Bladder Cancer | azelastine |
| Bladder Cancer | azilsartan medoxomil |
| Bladder Cancer | azithromycin |
| Bladder Cancer | benazepril |
| Bladder Cancer | benzthiazide |
| Bladder Cancer | betaxolol |
| Bladder Cancer | bezafibrate |
| Bladder Cancer | bicalutamide |
| Bladder Cancer | biperiden |
| Bladder Cancer | bortezomib |
| Bladder Cancer | bosentan |
| Bladder Cancer | bromfenac |
| Bladder Cancer | bromocriptine |
| Bladder Cancer | bumetanide |
| Bladder Cancer | bupropion |
| Bladder Cancer | cabozantinib |
| Bladder Cancer | caffeine |
| Bladder Cancer | candesartan |
| Bladder Cancer | captopril |
| Bladder Cancer | carbinoxamine |
| Bladder Cancer | carteolol |
| Bladder Cancer | cefazolin |
| Bladder Cancer | celecoxib |
| Bladder Cancer | cerulenin |
| Bladder Cancer | chloroquine |
| Bladder Cancer | chlorpheniramine |
| Bladder Cancer | chlorpromazine |
| Bladder Cancer | cilazapril |
| Bladder Cancer | cimetidine |
| Bladder Cancer | cinacalcet |
| Bladder Cancer | ciprofloxacin |
| Bladder Cancer | cisplatin |
| Bladder Cancer | citalopram |
| Bladder Cancer | cladribine |
| Bladder Cancer | clarithromycin |
| Bladder Cancer | clemastine |
| Bladder Cancer | clobetasol |
| Bladder Cancer | clodronate |
| Bladder Cancer | clofibrate |
| Bladder Cancer | clomipramine |
| Bladder Cancer | clotrimazole |
| Bladder Cancer | clozapine |
| Bladder Cancer | conjugated estrogens |
| Bladder Cancer | corticotropin |
| Bladder Cancer | cortisol |
| Bladder Cancer | Cyclosporin A |
| Bladder Cancer | cyproterone |
| Bladder Cancer | dabrafenib |
| Bladder Cancer | dalteparin |
| Bladder Cancer | danazol |
| Bladder Cancer | darifenacin |
| Bladder Cancer | decitabine |
| Bladder Cancer | delavirdine |
| Bladder Cancer | deprenyl |
| Bladder Cancer | desipramine |
| Bladder Cancer | desloratadine |
| Bladder Cancer | desmopressin |
| Bladder Cancer | desogestrel |
| Bladder Cancer | dexamethasone |
| Bladder Cancer | dexmedetomidine |
| Bladder Cancer | dextromethorphan |
| Bladder Cancer | diclofenac |
| Bladder Cancer | dicumarol |
| Bladder Cancer | dienestrol |
| Bladder Cancer | diethylcarbamazine |
| Bladder Cancer | diflunisal |
| Bladder Cancer | diltiazem |
| Bladder Cancer | diphenhydramine |
| Bladder Cancer | DMSO |
| Bladder Cancer | dopamine |
| Bladder Cancer | doxepin |
| Bladder Cancer | doxorubicin |
| Bladder Cancer | dronedarone |
| Bladder Cancer | drospirenone |
| Bladder Cancer | duloxetine |
| Bladder Cancer | EDTA |
| Bladder Cancer | efavirenz |
| Bladder Cancer | eicosapentaenoic acid |
| Bladder Cancer | eletriptan |
| Bladder Cancer | eltrombopag |
| Bladder Cancer | Enalapril |
| Bladder Cancer | enoxacin |
| Bladder Cancer | entacapone |
| Bladder Cancer | epinastine |
| Bladder Cancer | epinephrine |
| Bladder Cancer | epirubicin |
| Bladder Cancer | eprosartan |
| Bladder Cancer | erlotinib |
| Bladder Cancer | erythromycin |
| Bladder Cancer | escitalopram |
| Bladder Cancer | estradiol |
| Bladder Cancer | estramustine |
| Bladder Cancer | estriol |
| Bladder Cancer | estrone |
| Bladder Cancer | estropipate |
| Bladder Cancer | ethinyloestradiol |
| Bladder Cancer | ethynodiol diacetate |
| Bladder Cancer | etodolac |
| Bladder Cancer | etonogestrel |
| Bladder Cancer | etoposide |
| Bladder Cancer | etoricoxib |
| Bladder Cancer | everolimus |
| Bladder Cancer | felodipine |
| Bladder Cancer | fenofibrate |
| Bladder Cancer | fenoprofen |
| Bladder Cancer | fexofenadine |
| Bladder Cancer | fleroxacin |
| Bladder Cancer | fludrocortisone |
| Bladder Cancer | fluoxetine |
| Bladder Cancer | fluphenazine |
| Bladder Cancer | flurbiprofen |
| Bladder Cancer | flutamide |
| Bladder Cancer | fluvastatin |
| Bladder Cancer | fluvoxamine |
| Bladder Cancer | fondaparinux |
| Bladder Cancer | fosinopril |
| Bladder Cancer | gallium nitrate |
| Bladder Cancer | gefitinib |
| Bladder Cancer | gemfibrozil |
| Bladder Cancer | glipizide |
| Bladder Cancer | Glutathione |
| Bladder Cancer | GR138950 |
| Bladder Cancer | griseofulvin |
| Bladder Cancer | halofantrine |
| Bladder Cancer | heparin |
| Bladder Cancer | histamine |
| Bladder Cancer | Humalog |
| Bladder Cancer | hydergin |
| Bladder Cancer | hydrochlorothiazide |
| Bladder Cancer | hydroflumethiazide |
| Bladder Cancer | hydroxocobalamin |
| Bladder Cancer | hydroxychloroquine |
| Bladder Cancer | hydroxyurea |
| Bladder Cancer | hydroxyzine |
| Bladder Cancer | ibuprofen |
| Bladder Cancer | Icatibant |
| Bladder Cancer | idarubicin |
| Bladder Cancer | imatinib |
| Bladder Cancer | imipramine |
| Bladder Cancer | indinavir |
| Bladder Cancer | indomethacin |
| Bladder Cancer | insulin detemir |
| Bladder Cancer | irbesartan |
| Bladder Cancer | isoniazid |
| Bladder Cancer | isoproterenol |
| Bladder Cancer | itraconazole |
| Bladder Cancer | ketoconazole |
| Bladder Cancer | ketoprofen |
| Bladder Cancer | ketorolac |
| Bladder Cancer | labetalol |
| Bladder Cancer | lansoprazole |
| Bladder Cancer | Lantus |
| Bladder Cancer | lapatinib |
| Bladder Cancer | lenalidomide |
| Bladder Cancer | lercanidipine |
| Bladder Cancer | letrozole |
| Bladder Cancer | levofloxacin |
| Bladder Cancer | levonorgestrel |
| Bladder Cancer | lidocaine |
| Bladder Cancer | lipoic acid |
| Bladder Cancer | Lisinopril |
| Bladder Cancer | lithium |
| Bladder Cancer | L-methionine |
| Bladder Cancer | lomefloxacin |
| Bladder Cancer | lomustine |
| Bladder Cancer | lopinavir |
| Bladder Cancer | lornoxicam |
| Bladder Cancer | losartan |
| Bladder Cancer | lovastatin |
| Bladder Cancer | lucanthone |
| Bladder Cancer | lumefantrine |
| Bladder Cancer | lumiracoxib |
| Bladder Cancer | marimastat |
| Bladder Cancer | masoprocol |
| Bladder Cancer | meclofenamate |
| Bladder Cancer | medroxyprogesterone |
| Bladder Cancer | mefenamic acid |
| Bladder Cancer | mefloquine |
| Bladder Cancer | melatonin |
| Bladder Cancer | meloxicam |
| Bladder Cancer | memantine |
| Bladder Cancer | menadione |
| Bladder Cancer | mepyramine |
| Bladder Cancer | mestranol |
| Bladder Cancer | methadone |
| Bladder Cancer | methazolamide |
| Bladder Cancer | methimazole |
| Bladder Cancer | methylnaltrexone |
| Bladder Cancer | methylphenidate |
| Bladder Cancer | methyltestosterone |
| Bladder Cancer | metoclopramide |
| Bladder Cancer | metoprolol |
| Bladder Cancer | metyrapone |
| Bladder Cancer | mexiletine |
| Bladder Cancer | mianserin |
| Bladder Cancer | miconazole |
| Bladder Cancer | midodrine |
| Bladder Cancer | mifepristone |
| Bladder Cancer | mimosine |
| Bladder Cancer | minocycline |
| Bladder Cancer | mirabegron |
| Bladder Cancer | mitiglinide |
| Bladder Cancer | mitoxantrone |
| Bladder Cancer | moclobemide |
| Bladder Cancer | moexipril |
| Bladder Cancer | moxifloxacin |
| Bladder Cancer | mycophenolate mofetil |
| Bladder Cancer | mycophenolic acid |
| Bladder Cancer | nabumetone |
| Bladder Cancer | nafcillin |
| Bladder Cancer | naloxone |
| Bladder Cancer | naproxen |
| Bladder Cancer | nateglinide |
| Bladder Cancer | nelfinavir |
| Bladder Cancer | neomycin |
| Bladder Cancer | nepafenac |
| Bladder Cancer | nevirapine |
| Bladder Cancer | niacin |
| Bladder Cancer | nicardipine |
| Bladder Cancer | niclosamide |
| Bladder Cancer | nicotine |
| Bladder Cancer | nifedipine |
| Bladder Cancer | niflumic acid |
| Bladder Cancer | nilotinib |
| Bladder Cancer | nilutamide |
| Bladder Cancer | nilvadipine |
| Bladder Cancer | nisoldipine |
| Bladder Cancer | nitroprusside |
| Bladder Cancer | norepinephrine |
| Bladder Cancer | norfloxacin |
| Bladder Cancer | norgestimate |
| Bladder Cancer | nortriptyline |
| Bladder Cancer | NovoLog |
| Bladder Cancer | ofloxacin |
| Bladder Cancer | olanzapine |
| Bladder Cancer | olmesartan |
| Bladder Cancer | omeprazole |
| Bladder Cancer | ondansetron |
| Bladder Cancer | orlistat |
| Bladder Cancer | oxamniquine |
| Bladder Cancer | oxandrolone |
| Bladder Cancer | oxaprozin |
| Bladder Cancer | oxprenolol |
| Bladder Cancer | oxybutynin |
| Bladder Cancer | paclitaxel |
| Bladder Cancer | pantoprazole |
| Bladder Cancer | paroxetine |
| Bladder Cancer | pazopanib |
| Bladder Cancer | pefloxacin |
| Bladder Cancer | pentolinium |
| Bladder Cancer | pentosan polysulfate |
| Bladder Cancer | perhexiline |
| Bladder Cancer | perindopril |
| Bladder Cancer | perphenazine |
| Bladder Cancer | phenobarbital |
| Bladder Cancer | phenylbutazone |
| Bladder Cancer | phenylephrine |
| Bladder Cancer | Phosphoramidon |
| Bladder Cancer | pilocarpine |
| Bladder Cancer | pimecrolimus |
| Bladder Cancer | pimozide |
| Bladder Cancer | pioglitazone |
| Bladder Cancer | pipotiazine |
| Bladder Cancer | piroxicam |
| Bladder Cancer | podophyllotoxin |
| Bladder Cancer | pomalidomide |
| Bladder Cancer | ponatinib |
| Bladder Cancer | pranlukast |
| Bladder Cancer | pravastatin |
| Bladder Cancer | praziquantel |
| Bladder Cancer | prednisolone |
| Bladder Cancer | primaquine |
| Bladder Cancer | primidone |
| Bladder Cancer | progesterone |
| Bladder Cancer | proguanil |
| Bladder Cancer | promethazine |
| Bladder Cancer | propafenone |
| Bladder Cancer | propofol |
| Bladder Cancer | propranolol |
| Bladder Cancer | pyrimethamine |
| Bladder Cancer | quetiapine |
| Bladder Cancer | quinapril |
| Bladder Cancer | quinidine |
| Bladder Cancer | quinine |
| Bladder Cancer | rabeprazole |
| Bladder Cancer | raloxifene |
| Bladder Cancer | ramipril |
| Bladder Cancer | ranitidine |
| Bladder Cancer | ranolazine |
| Bladder Cancer | rapamycin |
| Bladder Cancer | rasagiline |
| Bladder Cancer | regorafenib |
| Bladder Cancer | repaglinide |
| Bladder Cancer | rescinnamine |
| Bladder Cancer | retigabine |
| Bladder Cancer | riboflavin |
| Bladder Cancer | ridogrel |
| Bladder Cancer | rifadin |
| Bladder Cancer | rifapentine |
| Bladder Cancer | rifaximin |
| Bladder Cancer | rilpivirine |
| Bladder Cancer | riluzole |
| Bladder Cancer | risedronate |
| Bladder Cancer | risperidone |
| Bladder Cancer | ritonavir |
| Bladder Cancer | rivastigmine |
| Bladder Cancer | ropinirole |
| Bladder Cancer | rosiglitazone |
| Bladder Cancer | rotigotine |
| Bladder Cancer | rutin |
| Bladder Cancer | ruxolitinib |
| Bladder Cancer | S-adenosylmethionine |
| Bladder Cancer | salicylate |
| Bladder Cancer | salsalate |
| Bladder Cancer | saquinavir |
| Bladder Cancer | secobarbital |
| Bladder Cancer | sertraline |
| Bladder Cancer | sildenafil |
| Bladder Cancer | simvastatin |
| Bladder Cancer | sorafenib |
| Bladder Cancer | sparfloxacin |
| Bladder Cancer | sparteine |
| Bladder Cancer | Spirapril |
| Bladder Cancer | spironolactone |
| Bladder Cancer | streptozotocin |
| Bladder Cancer | sulfaphenazole |
| Bladder Cancer | sulfasalazine |
| Bladder Cancer | sulindac |
| Bladder Cancer | sulodexide |
| Bladder Cancer | sunitinib |
| Bladder Cancer | tamibarotene |
| Bladder Cancer | tamoxifen |
| Bladder Cancer | tapentadol |
| Bladder Cancer | tasosartan |
| Bladder Cancer | tazarotene |
| Bladder Cancer | telithromycin |
| Bladder Cancer | telmisartan |
| Bladder Cancer | temsirolimus |
| Bladder Cancer | teniposide |
| Bladder Cancer | tenofovir |
| Bladder Cancer | tenoxicam |
| Bladder Cancer | terbinafine |
| Bladder Cancer | testosterone |
| Bladder Cancer | testosterone propionate |
| Bladder Cancer | tetrahydrobiopterin |
| Bladder Cancer | tetrahydrofolate |
| Bladder Cancer | theophylline |
| Bladder Cancer | thiabendazole |
| Bladder Cancer | thioridazine |
| Bladder Cancer | thiothixene |
| Bladder Cancer | tiaprofenic acid |
| Bladder Cancer | ticlopidine |
| Bladder Cancer | tioconazole |
| Bladder Cancer | tipranavir |
| Bladder Cancer | tirofiban |
| Bladder Cancer | tocainide |
| Bladder Cancer | tolmetin |
| Bladder Cancer | tramadol |
| Bladder Cancer | trandolapril |
| Bladder Cancer | tranylcypromine |
| Bladder Cancer | treprostinil |
| Bladder Cancer | triamcinolone |
| Bladder Cancer | triamterene |
| Bladder Cancer | trifluoperazine |
| Bladder Cancer | triflusal |
| Bladder Cancer | tripelennamine |
| Bladder Cancer | triprolidine |
| Bladder Cancer | troleandomycin |
| Bladder Cancer | Trospium chloride (Sanctura |
| Bladder Cancer | UDCA |
| Bladder Cancer | valproate |
| Bladder Cancer | valrubicin |
| Bladder Cancer | valsartan |
| Bladder Cancer | vandetanib |
| Bladder Cancer | varenicline |
| Bladder Cancer | vemurafenib |
| Bladder Cancer | venlafaxine |
| Bladder Cancer | verapamil |
| Bladder Cancer | vinblastine |
| Bladder Cancer | vinorelbine |
| Bladder Cancer | vitamin B |
| Bladder Cancer | vitamin D3 |
| Bladder Cancer | vitamin E |
| Bladder Cancer | vorinostat |
| Bladder Cancer | yohimbine |
| Bladder Cancer | zafirlukast |
| Bladder Cancer | zidovudine |
| Bladder Cancer | ziprasidone |
| Bladder Cancer | zonisamide |

## Supplemental table 2

1. List of the 24 pathways that were integrated to create the integrated cancer pathway

| Pathway Name | Source |
| --- | --- |
| Molecular Mechanisms of Cancer | Protein Lounge |
| P53 Signaling | Protein Lounge |
| Cell Cycle | KEGG |
| Chronic Myeloid Leukemia | KEGG |
| P53 signaling molecule | KEGG |
| Pancreatic Cancer | KEGG |
| DNA repair mechanism | Protein Lounge |
| ATM Signaling pathway | BioCarta |
| Aurora A signaling | NCI Nature curated |
| Role of BRCA1, BRCA2, and ATR in cancer suseption | BioCarta |
| Apoptosis | KEGG |
| BRCA1 Pathway | Protein Lounge |
| Cell cycle: checkpoint | BioCarta |
| Regulation of Telomerase | NCI-Nature Curated |
| Prostate Cancer | KEGG |
| Breast Cancer Regulation by Stathmin1 | Protein Lounge |
| Non-small cell lung cancer | KEGG |
| Endometrial Cancer | KEGG |
| Colorectal cancer | KEGG |
| Pancreatic Cancer | KEGG |
| Pathways in Cancer | KEGG |
| Transcriptional misregulation in cancer | KEGG |
| PTEN Pathway | Protein Lounge |
| EGF Pathway | Protein Lounge |

1. Protein-protein interactions in Breast Cancer pathway model

| Interactor | Interactee | Mechanism of Action |
| --- | --- | --- |
| SELK | C9JNK1 | activation |
| RAC1 | C9JNK1 | activation |
| MEK1 | C9JNK1 | activation |
| ITPKC | C9JNK1 | activation |
| C9JNK1 | P53 | activation |
| HIPK2 | P53 | activation |
| CSNK1D | P53 | activation |
| ATR | P53 | activation |
| DCAKD | P53 | activation |
| SIRT1 | P53 | inhibition |
| ATM | P53 | activation |
| CHK2 | P53 | activation |
| CHK1 | P53 | activation |
| HDAC1 | P53 | inhibition |
| MDM2 | P53 | inhibition |
| FADD | P53 | activation |
| TRADD | P53 | activation |
| CBP | P53 | activation |
| AURKA | P53 | inhibition |
| LIG1/3 | P53 | activation |
| CHK2 | BRCA1 | activation |
| ESR1 | BRCA1 | activation |
| BARD1 | BRCA1 | activation |
| E2F1 | SIRT1 | activation |
| FOXO1 | SIRT1 | activation |
| ATF1 | CDK2 | activation |
| MYC | CDK2 | inhibition |
| CIP1 | CDK2 | inhibition |
| JKIP1 | CDK2 | inhibition |
| SP1 | CDK2 | inhibition |
| CDK2 | CDK4 | inhibition |
| CDC25A | CDK4 | activation |
| UBP15 | CDK4 | inhibition |
| UBP16 | CDK4 | inhibition |
| BRCA1 | E2F1 | activation |
| CDK2 | E2F1 | activation |
| CDK4 | E2F1 | activation |
| PPRB/RB | E2F1 | activation |
| UBP16 | E2F1 | activation |
| NFKB1 | E2F1 | activation |
| CYP19A1 | E2F1 | activation |
| P53 | PTEN | activation |
| NFKB1 | PTEN | activation |
| ATR | CHK2 | activation |
| ATM | CHK2 | activation |
| BRCA1 | STAT1 | activation |
| JAK1 | STAT1 | activation |
| HIPK2 | ATF1 | activation |
| BRCA1 | ATF1 | activation |
| ATR | CHK1 | activation |
| ATM | CHK1 | activation |
| CDH1 | CHK1 | activation |
| BRCA1 | PLK3CA | activation |
| BRCA1 | HDAC1 | activation |
| BRCA1 | BLM | activation |
| BRCA1 | MSH2 | activation |
| BRCA1 | MSH6 | activation |
| BRCA1 | Q8NBS1 | activation |
| BRCA1 | MRE11 | activation |
| BRCA1 | RAD50 | activation |
| SP1 | DAG1 | activation |
| DAG1 | PKC-beta | activation |
| nNOS | PKC-beta | activation |
| GRIA3 | PKC-beta | activation |
| PKC-beta | RASGRP3 | activation |
| RASGRP3 | KRAS | activation |
| RASGEF1A | KRAS | activation |
| BCL2 | KRAS | activation |
| ERAL1 | KRAS | activation |
| P13K | KRAS | activation |
| ABC3G | RAP1A | activation |
| IPKA | RAP1A | activation |
| RAP1A | BRAF | activation |
| RAS | BRAF | activation |
| RASGRP3 | MPIP1 | activation |
| KRAS | MPIP1 | activation |
| NF1 | RAS | inhibition |
| BRAF | SMEK2 | activation |
| SMEK2 | CERK1 | activation |
| ANXA1 | CERK1 | inhibition |
| CERK1 | SELK | activation |
| RALGAPA1 | RAC1 | activation |
| CDC42 | RAC1 | activation |
| RALA | RALGAPA1 | activation |
| RHO | CDC42 | activation |
| ALKB1 | TSC1 | activation |
| AKT1 | TSC1 | inhibition |
| STK11 | TSC1 | activation |
| TSC1 | RHEB | inhibition |
| TSC2 | RHEB | inhibition |
| MPIP1 | AKT1 | activation |
| ERAL1 | AKT1 | activation |
| P13K | AKT1 | activation |
| P13 | AKT1 | activation |
| MPIP2 | AKT1 | activation |
| RHO | AKT1 | activation |
| ATF1 | GSK3A | inhibition |
| AKT1 | GSK3A | inhibition |
| CDK4 | CCND1 | activation |
| E2F1 | CCND1 | activation |
| PTEN | CCND1 | inhibition |
| KRAS | CCND1 | activation |
| AKT1 | CCND1 | activation |
| GSK3A | CCND1 | inhibition |
| BCL2 | CCND1 | activation |
| SMAD4 | CCND1 | inhibition |
| MYC | CCND1 | activation |
| ESR1 | CCND1 | inhibition |
| JUN | CCND1 | activation |
| SP1 | CCND1 | activation |
| NFKB1 | CCND1 | activation |
| P53 | MDM2 | activation |
| KRAS | MDM2 | activation |
| AKT1 | MDM2 | activation |
| P53 | NOXA1 | activation |
| AKT1 | BAD | inhibition |
| BCL2 | BAD | inhibition |
| GRN | BAD | activation |
| AKT2 | BAD | inhibition |
| AKT3 | BAD | inhibition |
| AKT1 | PAK1 | activation |
| PTEN | BCL2 | inhibition |
| AKT1 | BCL2 | activation |
| NOXA1 | BCL2 | inhibition |
| BAD | BCL2 | inhibition |
| UBIM | BCL2 | inhibition |
| EGFR | BCL2 | activation |
| VEGFA | BCL2 | activation |
| JUN | BCL2 | activation |
| CASP8 | BCL2 | inhibition |
| NCOA2 | BCL2 | activation |
| NFKB1 | BCL2 | activation |
| CDH1 | BCL2 | inhibition |
| BID | UBIM | activation |
| CDC42 | MMP1 | inhibition |
| MDM2 | MMP1 | activation |
| BCL2 | MMP1 | activation |
| BAK | MMP1 | activation |
| EGFR | MMP1 | activation |
| ESR1 | MMP1 | activation |
| JUN | MMP1 | activation |
| BRCA1 | PPRB/RB | activation |
| CDK2 | PPRB/RB | inhibition |
| CDK4 | PPRB/RB | inhibition |
| E2F1 | PPRB/RB | inhibition |
| UBP16 | PPRB/RB | inhibition |
| GADD45A | PPRB/RB | activation |
| ANDR | PPRB/RB | activation |
| CDK7 | PPRB/RB | activation |
| CDC25A | WEE1 | inhibition |
| CHK2 | CDC25A | inhibition |
| CHK1 | CDC25A | inhibition |
| WEE1 | CDC25A | inhibition |
| MYC | CDC25A | activation |
| PLK1 | CDC25A | activation |
| ATR | NAB1 | activation |
| ATM | NAB1 | activation |
| NAB1 | RAD51 | activation |
| CERK1 | SMAD4 | activation |
| SMAD2 | SMAD4 | activation |
| SMAD1 | SMAD4 | activation |
| SMAD3 | SMAD4 | activation |
| TGFR1 | SMAD4 | activation |
| TGFR2 | SMAD4 | activation |
| SMAD4 | UBE2F | activation |
| SMAD1 | UBE2F | activation |
| SMAD3 | UBE2F | activation |
| SMAD4 | SMAD2 | activation |
| RPP38 | MYC | activation |
| ESR1 | MYC | activation |
| SP1 | MYC | activation |
| GDI | MYC | activation |
| NFKB1 | MYC | activation |
| STK11 | MYC | inhibition |
| CTNB1 | MYC | activation |
| TCF/LEF | MYC | activation |
| ERR2 | MYC | activation |
| SMAD2 | MAX | inhibition |
| MYC | MAX | activation |
| RPP38 | MAX | activation |
| JUN | MAX | activation |
| fra-1 | MAX | activation |
| cyc-D | MAX | activation |
| PPAR | MAX | activation |
| SMAD2 | ZMIZ1 | inhibition |
| BMPR1A | BMPR2 | activation |
| SMAD7 | BMPR2 | inhibition |
| SIRT1 | SMAD7 | inhibition |
| SMAD3 | SMAD7 | activation |
| CERK1 | SMAD1 | activation |
| BMPR2 | SMAD1 | activation |
| SMAD6 | SMAD1 | inhibition |
| CERK1 | SMAD3 | activation |
| BMPR2 | SMAD3 | activation |
| SMAD6 | SMAD3 | inhibition |
| SMAD7 | TGFR1 | inhibition |
| SMAD7 | TGFR2 | inhibition |
| TGFR1 | TAK1L | activation |
| TGFR2 | TAK1L | activation |
| TGFR1 | TAB1 | activation |
| TGFR2 | TAB1 | activation |
| TAK1L | RPP38 | activation |
| TAB1 | RPP38 | activation |
| SMAD2 | CIP1 | activation |
| UBE2F | JKIP1 | activation |
| SMAD2 | JKIP1 | activation |
| SMAD2 | UBP15 | activation |
| CIP1 | UBP16 | inhibition |
| JKIP1 | UBP16 | inhibition |
| C9JNK1 | FADD | activation |
| FLIP1 | FADD | inhibition |
| C9JNK1 | TRADD | activation |
| FLIP1 | TRADD | inhibition |
| FADD | BID | activation |
| TRADD | BID | activation |
| P53 | UBP21 | activation |
| ESR1 | EGFR | activation |
| SP1 | EGFR | activation |
| NFKB1 | EGFR | activation |
| EGFR | JAK1 | activation |
| nAChR | JAK1 | activation |
| PTEN | VEGFA | inhibition |
| STAT1 | VEGFA | inhibition |
| KRAS | VEGFA | inhibition |
| MDM2 | VEGFA | activation |
| BCL2 | VEGFA | activation |
| MYC | VEGFA | activation |
| GRN | VEGFA | activation |
| JUN | VEGFA | activation |
| NFKB1 | VEGFA | activation |
| MEK1 | CERK | activation |
| KRAS | MEK1 | activation |
| RAS | MEK1 | activation |
| RAF | MEK1 | activation |
| AKT1 | IKKA | activation |
| RHO | IKKA | activation |
| P53 | B3KP27 | activation |
| AKT1 | B3KP27 | inhibition |
| RAS | RAF | activation |
| Raf | RAF | activation |
| Ras | RAF | activation |
| P53 | AQP73 | activation |
| AKT1 | AQP73 | inhibition |
| BRCA1 | GADD45A | activation |
| MYC | GADD45A | inhibition |
| IMPA1 | GADD45A | activation |
| DHT | GADD45A | activation |
| GCR | GADD45A | activation |
| CDC25B | GADD45A | activation |
| NROB1 | GADD45A | activation |
| MYT1 | MYT1 | inhibition |
| PLK1 | MYT1 | inhibition |
| PLK1 | PLK1 | activation |
| BCL2 | BAX | inhibition |
| JUN | BAX | activation |
| NFKB1 | BAX | activation |
| SP1 | GRN | inhibition |
| JAK1 | ERAL1 | activation |
| ESR1 | ERAL1 | activation |
| Fyn | ERAL1 | activation |
| JAK1 | P13K | activation |
| ESR1 | P13K | activation |
| Fyn | P13K | activation |
| IMPA1 | PR | activation |
| E2F1 | NCOA3 | activation |
| DHT | NCOA3 | activation |
| ERR2 | NCOA3 | activation |
| ESR2 | NCOA3 | activation |
| BRCA1 | ESR1 | inhibition |
| E2F1 | ESR1 | activation |
| RALA | ESR1 | activation |
| AKT1 | ESR1 | activation |
| SMAD2 | ESR1 | inhibition |
| SMAD3 | ESR1 | inhibition |
| GADD45A | ESR1 | inhibition |
| SMCA4 | ESR1 | activation |
| MAPKs | ESR1 | activation |
| ANDR | ESR1 | inhibition |
| NCOA2 | ESR1 | activation |
| NFKB1 | ESR1 | inhibition |
| AHR | ESR1 | activation |
| ERR3 | ESR1 | activation |
| SIRT1 | JUN | activation |
| ATF1 | JUN | activation |
| KRAS | JUN | activation |
| MYC | JUN | activation |
| RPP38 | JUN | activation |
| EGFR | JUN | inhibition |
| JAK1 | JUN | inhibition |
| VEGFA | JUN | activation |
| NCOA3 | JUN | inhibition |
| ESR1 | JUN | activation |
| GDI | JUN | activation |
| ABL1 | JUN | activation |
| TFPI | JUN | activation |
| CTNB1 | JUN | activation |
| TCF/LEF | JUN | activation |
| ERR2 | JUN | activation |
| ATR | BRCA2 | activation |
| ATM | BRCA2 | activation |
| SMCA4 | BRCA2 | activation |
| NFKB1 | BRCA2 | activation |
| RAD51 | RAD54L | activation |
| BRCA2 | RAD54L | activation |
| XRCC3 | RAD54L | activation |
| ATAD5 | RAD54L | activation |
| BRCA1 | BARD1 | activation |
| E2F1 | BARD1 | activation |
| C9JNK1 | SP1 | activation |
| MYC | SP1 | activation |
| ERR1 | SP1 | activation |
| ESR2 | SP1 | activation |
| ESRRA | SP1 | activation |
| ESR1 | P13 | activation |
| P85 | P13 | activation |
| IRS1 | P13 | inhibition |
| GR | P13 | activation |
| KRAS | ERK | activation |
| P13 | ERK | activation |
| PKA | ERK | activation |
| CaMK | ERK | activation |
| ESR1 | P38 | activation |
| BRCA1 | ANDR | activation |
| IMPA1 | ANDR | activation |
| DHT | ANDR | activation |
| GCR | ANDR | activation |
| CDC25B | ANDR | activation |
| NROB1 | ANDR | activation |
| CASP3 | GDI | activation |
| BCL2 | CASP3 | activation |
| VEGFA | CASP3 | inhibition |
| BAX | CASP3 | activation |
| CASP8 | CASP3 | activation |
| CASP9 | CASP3 | activation |
| ABL1 | P85 | activation |
| NAB1 | XRCC3 | activation |
| CHK2 | TFPI | activation |
| HDAC1/HDAC2 | TFPI | inhibition |
| RAD54L | HDAC1/HDAC2 | activation |
| DHT | P85A | activation |
| ERR2 | P85A | activation |
| ESR2 | P85A | activation |
| P53 | GCR | inhibition |
| ATR | PLK3 | activation |
| ATM | PLK3 | activation |
| CERK | NCOA2 | activation |
| AKT1 | CREB | activation |
| ERK | CREB | activation |
| SIRT1 | FOXO1 | activation |
| E2F1 | FOXO1 | activation |
| AKT1 | FOXO1 | activation |
| NCOA3 | IRS1 | inhibition |
| AKT1 | CASP9 | inhibition |
| BCL2 | CASP9 | activation |
| BAX | CASP9 | activation |
| CytC | CASP9 | activation |
| Apaf-1 | CASP9 | activation |
| AKT2 | CASP9 | inhibition |
| AKT3 | CASP9 | inhibition |
| E2F1 | NFKB1 | activation |
| IKKA | NFKB1 | activation |
| NFKB1 | NFKB1 | activation |
| ALKB1 | TSC2 | activation |
| AKT1 | TSC2 | inhibition |
| STK11 | TSC2 | activation |
| TSC1 | MTOR | activation |
| RHEB | MTOR | activation |
| TSC2 | MTOR | activation |
| MYC | CDH1 | activation |
| VEGFA | CDH1 | activation |
| NFKB1 | CDH1 | inhibition |
| AKT1 | EP300 | activation |
| ERK | EP300 | activation |
| KRAS | MPIP2 | activation |
| GR | ANXA1 | activation |
| RAS | RHO | activation |
| GEF-H1 | RHO | activation |
| TUBB1 | RHO | activation |
| TUBAL3 | RHO | activation |
| TUBA1C | RHO | activation |
| TUBB6 | RHO | activation |
| TUBA3C | RHO | activation |
| TUBB8 | RHO | activation |
| TUBA1A | RHO | activation |
| JUN | CDK7 | inhibition |
| ANXA1 | P65 | inhibition |
| GADD45A | CYP19A1 | activation |
| ANDR | CYP19A1 | activation |
| MYC | ODC1 | activation |
| MAX | ODC1 | activation |
| CTNB1 | TCF/LEF | activation |
| RPP38 | fra-1 | activation |
| ESR1 | fra-1 | activation |
| GDI | fra-1 | activation |
| CTNB1 | fra-1 | activation |
| TCF/LEF | fra-1 | activation |
| ERR2 | fra-1 | activation |
| RPP38 | cyc-D | activation |
| ESR1 | cyc-D | activation |
| GDI | cyc-D | activation |
| CTNB1 | cyc-D | activation |
| TCF/LEF | cyc-D | activation |
| ERR2 | cyc-D | activation |
| RPP38 | PPAR | activation |
| ESR1 | PPAR | activation |
| GDI | PPAR | activation |
| CTNB1 | PPAR | activation |
| TCF/LEF | PPAR | activation |
| ERR2 | PPAR | activation |
| DAG1 | UNC1 | activation |
| ACHE | M5 | activation |
| UNC1 | ACHE | activation |
| M5 | Gq11 | activation |
| Gq11 | PLC | activation |
| ACHE | M2 | activation |
| ACHE | M4 | activation |
| ACHE | nAChR | activation |
| nAChR | AC | activation |
| ERK | c-fos | activation |
| AC | PKA | activation |
| sAC | PKA | activation |
| nAChR | CaMK | activation |
| nAChR | Fyn | activation |
| ABC3G | FUMP | activation |
| FUMP | FUDP | activation |
| FUDP | RRM1 | activation |
| RRM1 | FdUDP | activation |
| FdUDP | FdUMP | activation |
| FdUMP | TYMS | inhibition |
| TYMS | FdUTP | activation |
| FdUTP | FdUTP | activation |
| BCL2 | CytC | inhibition |
| BCL2 | Apaf-1 | inhibition |
| ESPG | TUBA | activation |
| ESPG | TUBB | activation |
| TUBA | GEF-H1 | activation |
| TUBB | GEF-H1 | activation |
| RHO | ROCK | activation |
| PKC-beta | NOS | activation |
| NOS | NO | activation |
| NO | sAC | activation |
| NO | sGC | activation |
| sGC | PKG | activation |
| ESPG | TUBB1 | activation |
| ESPG | TUBAL3 | activation |
| ESPG | TUBB6 | activation |
| ESPG | TUBA3C | activation |
| ESPG | TUBB8 | activation |
| ESPG | TUBA1A | activation |
| Sos | Raf | activation |
| Grb2 | Sos | activation |
| Shc | Grb2 | activation |
| ERBB2 | Shc | activation |
| CHK1 | TLK1/2 | inhibition |
| CHK1 | CDC25C | activation |
| CDC25C | CDC2 | inhibition |
| CHK1 | CDC25B | activation |
| CDK1 | CDC25B | activation |
| ESR1 | ERR1 | activation |
| ESR1 | ERR2 | activation |
| CREB | ESR2 | activation |
| EP300 | ESR2 | activation |
| SP1 | ESRRA | activation |
| EGFR | GRB2 | activation |
| GRB2 | SOS | activation |
| SOS | Ras | activation |
| PRKCA | Ras | activation |

## Supplemental table 3

Drug-protein interactions for Breast Cancer case study

| Drug | Protein | Quantified interaction |
| --- | --- | --- |
| Anastrozole | CYP19A1 | inhibition |
| Capecitabine | TYMS | inhibition |
| Cycloheximide | P53 | inhibition |
| Cycloheximide | BRCA1 | inhibition |
| Cycloheximide | E2F1 | inhibition |
| Cycloheximide | CHK1 | inhibition |
| Cycloheximide | BLM | activation |
| Cycloheximide | MSH2 | activation |
| Cycloheximide | MSH6 | activation |
| Cycloheximide | Q8NBS1 | activation |
| Cycloheximide | MRE11 | activation |
| Cycloheximide | RAD50 | activation |
| Cycloheximide | AKT1 | activation |
| Cycloheximide | CDC25A | inhibition |
| Cycloheximide | MYC | activation |
| Cycloheximide | GADD45A | inhibition |
| Cycloheximide | NCOA3 | activation |
| Cycloheximide | ESR1 | activation |
| Cycloheximide | JUN | activation |
| Cycloheximide | BRCA2 | activation |
| Cycloheximide | SP1 | activation |
| Cycloheximide | ANDR | inhibition |
| Cycloheximide | CASP8 | inhibition |
| Cycloheximide | CASP3 | activation |
| Cycloheximide | CBP | inhibition |
| Cycloheximide | P85A | activation |
| Cycloheximide | CREB | activation |
| Cycloheximide | EP300 | activation |
| Cycloheximide | MPIP2 | inhibition |
| Cycloheximide | CYP19A1 | inhibition |
| Cycloheximide | fra-1 | activation |
| Cycloheximide | cyc-D | activation |
| Cycloheximide | PPAR | activation |
| Docetaxel | TUBB1 | activation |
| Doxorubicin | P53 | activation |
| Doxorubicin | AKT1 | activation |
| Doxorubicin | ABCB1 | activation |
| Epirubicin | CDH1 | inhibition |
| Exemestane | CYP19A1 | inhibition |
| Fluorouracil | P53 | activation |
| Fluoxymesterone | GADD45A | activation |
| Fluoxymesterone | ESR1 | inhibition |
| Fluoxymesterone | ANDR | activation |
| Fulvestrant | ESR1 | inhibition |
| Gemcitabine | RRM1 | inhibition |
| Gemcitabine | TYMS | inhibition |
| Lapatinib | EGFR | inhibition |
| Lapatinib | ERBB2 | inhibition |
| Letrozole | CYP19A1 | inhibition |
| Methotrexate | DHFR | inhibition |
| Miltefosine | AKT1 | inhibition |
| Paclitaxel | BCL2 | inhibition |
| Paclitaxel | TUBA | inhibition |
| Paclitaxel | TUBB | inhibition |
| Pamidronate | BCL2 | inhibition |
| Raloxifene | ESR1 | inhibition |
| Raloxifene | ESR2 | activation |
| Tamoxifen | C9JNK1 | activation |
| Tamoxifen | P53 | activation |
| Tamoxifen | CDK2 | activation |
| Tamoxifen | E2F1 | activation |
| Tamoxifen | PTEN | activation |
| Tamoxifen | AKT1 | activation |
| Tamoxifen | MYC | inhibition |
| Tamoxifen | UBP16 | activation |
| Tamoxifen | ESR1 | inhibition |
| Tamoxifen | JUN | activation |
| Tamoxifen | BRCA2 | activation |
| Tamoxifen | IRS1 | inhibition |
| Tamoxifen | CDH1 | activation |
| Tamoxifen | fra-1 | activation |
| Tamoxifen | cyc-D | activation |
| Tamoxifen | PPAR | activation |
| Tamoxifen | ESR2 | inhibition |
| Thiotepa | CASP3 | activation |
| Toremifene | ERBB2 | activation |
| Trastuzumab Emtansine | ERBB2 | inhibition |
| Vinblastine | TUBB | activation |
| Vinblastine | TUBA1A | activation |
| Diethylstilbestrol | ESR1 | activation |
| Diethylstilbestrol | ERR1 | inhibition |
| Diethylstilbestrol | ERR2 | inhibition |
| Diethylstilbestrol | ERR3 | activation |
| Diethylstilbestrol | ESR2 | activation |
| Diethylstilbestrol | ESRRA | activation |
| Dromostanolone Propionate | ESR1 | activation |
| Dromostanolone Propionate | ANDR | activation |
| Formestane | ESR1 | activation |
| Formestane | CYP19A1 | inhibition |
| Ixabepilone | TUBB | inhibition |
| Testolactone | CYP19A1 | inhibition |
| Vorozole | CYP19A1 | inhibition |
| Avastin | VEGFA | inhibition |
| Ethyl Carbamate | ESR1 | activation |
| Tivozanib | MTOR | inhibition |
| Imetelstat | PLK3CA | inhibition |
| Imetelstat | EGFR | inhibition |
| Imetelstat | ABL1 | inhibition |
| Bleomycin | RAD51 | activation |
| Bleomycin | SMAD4 | inhibition |
| Bleomycin | SMAD1 | inhibition |
| Bleomycin | SMAD3 | inhibition |
| Bleomycin | XRCC3 | activation |
| Corticosterone | MYC | inhibition |
| Corticosterone | JUN | inhibition |
| Corticosterone | GCR | inhibition |
| Corticosterone | fra-1 | inhibition |
| Corticosterone | cyc-D | inhibition |
| Corticosterone | PPAR | inhibition |
| Corticosterone | NCOA1 | activation |
| Daunorubicin | MDR1 | activation |
| Daunorubicin | ABCB1 | activation |
| Dexamethasone | P53 | inhibition |
| Dexamethasone | CDK2 | inhibition |
| Dexamethasone | PLK3CA | activation |
| Dexamethasone | AKT1 | inhibition |
| Dexamethasone | CDC25A | activation |
| Dexamethasone | UBP16 | inhibition |
| Dexamethasone | IKKA | activation |
| Dexamethasone | RAF | activation |
| Dexamethasone | GADD45A | activation |
| Dexamethasone | GRN | activation |
| Dexamethasone | NCOA3 | activation |
| Dexamethasone | ESR1 | activation |
| Dexamethasone | SP1 | activation |
| Dexamethasone | ANDR | activation |
| Dexamethasone | CASP8 | activation |
| Dexamethasone | P85A | activation |
| Dexamethasone | CREB | activation |
| Dexamethasone | FOXO1 | activation |
| Dexamethasone | IRS1 | inhibition |
| Dexamethasone | CDH1 | activation |
| Dexamethasone | EP300 | activation |
| Dexamethasone | CTNB1 | activation |
| Dexamethasone | ANXA1 | activation |
| Dexamethasone | NROB1 | activation |
| Dihydrotestosterone | SMAD4 | inhibition |
| Dihydrotestosterone | SMAD1 | activation |
| Dihydrotestosterone | SMAD3 | activation |
| Dihydrotestosterone | GADD45A | activation |
| Dihydrotestosterone | DHT | activation |
| Dihydrotestosterone | ANDR | activation |
| Dihydrotestosterone | GCR | inhibition |
| Donepezil | CREB | activation |
| Donepezil | EP300 | activation |
| Donepezil | ACHE | inhibition |
| Erbitux | EGFR | inhibition |
| Ethinyl Estradiol | ESR1 | activation |
| Flutamide | PPRB/RB | inhibition |
| Flutamide | GADD45A | inhibition |
| Flutamide | ANDR | inhibition |
| Flutamide | AHR | activation |
| Hydrocortisone | GADD45A | activation |
| Hydrocortisone | ANDR | activation |
| Hydrocortisone | GCR | inhibition |
| Hydrocortisone | ANXA1 | activation |
| Hydroxyurea | BRCA1 | inhibition |
| Hydroxyurea | CDK2 | activation |
| Hydroxyurea | CHK2 | activation |
| Hydroxyurea | CHK1 | inhibition |
| Hydroxyurea | BLM | activation |
| Hydroxyurea | MSH2 | inhibition |
| Hydroxyurea | MSH6 | inhibition |
| Hydroxyurea | Q8NBS1 | inhibition |
| Hydroxyurea | MRE11 | inhibition |
| Hydroxyurea | RAD50 | inhibition |
| Hydroxyurea | CDC25A | inhibition |
| Hydroxyurea | UBP16 | activation |
| Hydroxyurea | RRM1 | inhibition |
| Lithium Chloride | GSK3A | inhibition |
| Lithium Chloride | IMPA1 | inhibition |
| Lithium Chloride | CTNB1 | activation |
| Medrysone | GCR | activation |
| Methyl Methanesulfonate | CHK1 | activation |
| Methyl Methanesulfonate | BLM | activation |
| Methyl Methanesulfonate | MSH2 | activation |
| Methyl Methanesulfonate | MSH6 | activation |
| Methyl Methanesulfonate | Q8NBS1 | activation |
| Methyl Methanesulfonate | MRE11 | activation |
| Methyl Methanesulfonate | RAD50 | activation |
| Methyl Methanesulfonate | RAD51 | activation |
| Methyl Methanesulfonate | MYC | activation |
| Methyl Methanesulfonate | JUN | activation |
| Methyl Methanesulfonate | XRCC3 | activation |
| Methyl Methanesulfonate | fra-1 | activation |
| Methyl Methanesulfonate | cyc-D | activation |
| Methyl Methanesulfonate | PPAR | activation |
| Methylprednisolone | GCR | activation |
| Mitomycin | P53 | inhibition |
| Mitomycin | BLM | activation |
| Mitomycin | MSH2 | activation |
| Mitomycin | MSH6 | activation |
| Mitomycin | Q8NBS1 | activation |
| Mitomycin | MRE11 | activation |
| Mitomycin | RAD50 | activation |
| Mitomycin | PPRB/RB | inhibition |
| Mitomycin | RAD51 | activation |
| Mitomycin | BRCA2 | inhibition |
| Mitomycin | XRCC3 | activation |
| Mitomycin | nNOS | inhibition |
| Nocodazole | P53 | activation |
| Nocodazole | CHK1 | activation |
| Nocodazole | CDC25A | inhibition |
| Nocodazole | RAF | activation |
| Nocodazole | ESR1 | inhibition |
| Norethindrone | PR | activation |
| Pemetrexed | TYMS | inhibition |
| Pemetrexed | DHFR | inhibition |
| Plicamycin | MYC | inhibition |
| Plicamycin | JUN | inhibition |
| Plicamycin | SP1 | inhibition |
| Plicamycin | fra-1 | inhibition |
| Plicamycin | cyc-D | inhibition |
| Plicamycin | PPAR | inhibition |
| Prednisolone | GCR | activation |
| Prednisone | GCR | activation |
| Progesterone | ESR1 | activation |
| Testosterone | GADD45A | activation |
| Testosterone | ANDR | activation |
| Trilostane | ESR1 | activation |
| Trilostane | ESR2 | activation |
| Vandetanib | EGFR | inhibition |

## Supplemental table 4

Initial condition vector in Breast Cancer ER-positive subtype and ER-negative subtype after GEO2R preprocessing.

| Protein name | ER-positive | ER-Negative |
| --- | --- | --- |
| C9JNK1 | 0 | 1 |
| HIPK2 | -1 | -1 |
| CSNK1D | 0 | 0 |
| P53 | 0 | 0 |
| ATR | 0 | 0 |
| DCAKD | 0 | 0 |
| BRCA1 | 0 | 0 |
| PML | 0 | 0 |
| PIAS1 | 0 | 0 |
| SIRT1 | 0 | 0 |
| CDK2 | 0 | 0 |
| CDK4 | 0 | 1 |
| E2F1 | 0 | 1 |
| PTEN | 0 | 0 |
| ATM | 0 | 0 |
| CHK2 | 0 | 0 |
| STAT1 | 1 | 1 |
| ATF1 | 0 | 0 |
| CHK1 | 0 | 0 |
| PLK3CA | 0 | 0 |
| HDAC1 | 0 | 0 |
| BLM | 0 | 1 |
| MSH2 | 0 | 1 |
| MSH6 | 0 | 1 |
| Q8NBS1 | 0 | 0 |
| MRE11 | 0 | 0 |
| RAD50 | 1 | 0 |
| BACH1 | 0 | 0 |
| DAG1 | -1 | -1 |
| PKC-beta | 0 | 0 |
| RASGRP3 | 1 | 0 |
| KRAS | 1 | 0 |
| RASGEF1A | 0 | 0 |
| ABC3G | 0 | 0 |
| RAP1A | 0 | 0 |
| IPKA | 0 | 0 |
| BRAF | 0 | 0 |
| MPIP1 | 0 | 0 |
| RAS | 0 | 0 |
| SMEK2 | 0 | 0 |
| CERK1 | 0 | 0 |
| SELK | 0 | 0 |
| NF1 | 0 | 0 |
| RAC1 | 0 | 0 |
| RALGAPA1 | 0 | 0 |
| RALA | 0 | 0 |
| CDC42 | 0 | 0 |
| ALKB1 | 0 | 0 |
| TSC1 | 0 | 0 |
| RHEB | 0 | 0 |
| AKT1 | 0 | 0 |
| GSK3A | 1 | 1 |
| CCND1 | 0 | 0 |
| MDM2 | -1 | 0 |
| NOXA1 | 0 | 0 |
| BAD | -1 | 0 |
| PAK1 | 1 | 1 |
| BCL2 | -1 | -1 |
| UBIM | 0 | 0 |
| MMP1 | 1 | 1 |
| BAK | 0 | 0 |
| PPRB/RB | 0 | 0 |
| WEE1 | 0 | 0 |
| CDC25A | 0 | 0 |
| NAB1 | -1 | 0 |
| RAD51 | 1 | 1 |
| SMAD4 | 0 | 0 |
| UBE2F | 0 | 0 |
| SMAD2 | -1 | 0 |
| MYC | -1 | -1 |
| MAX | 1 | 0 |
| ZMIZ1 | 0 | 0 |
| BMPR1A | 0 | 0 |
| BMPR2 | 0 | 0 |
| SMAD7 | 0 | 0 |
| SMAD1 | 0 | 0 |
| SMAD3 | 0 | 0 |
| SMAD6 | -1 | 0 |
| TGFR1 | 0 | 0 |
| TGFR2 | 0 | 0 |
| TAK1L | 0 | 0 |
| TAB1 | 0 | 0 |
| RPP38 | 1 | 1 |
| CIP1 | 0 | 0 |
| JKIP1 | 0 | 0 |
| UBP15 | 0 | 0 |
| UBP16 | 0 | 0 |
| FADD | 0 | 0 |
| TRADD | 0 | 0 |
| FLIP1 | 0 | 0 |
| BID | 0 | 1 |
| UBP21 | 0 | 0 |
| B5MEC1 | 0 | 0 |
| EGFR | -1 | 0 |
| JAK1 | 0 | 0 |
| VEGFA | 0 | 0 |
| CERK | 0 | 0 |
| MEK1 | 0 | 0 |
| IKKA | 0 | 0 |
| B3KP27 | 0 | 0 |
| RAF | 0 | 0 |
| AQP73 | 0 | 0 |
| GADD45A | 0 | 0 |
| MYT1 | 0 | 0 |
| PLK1 | 0 | 0 |
| BAX | 1 | 1 |
| GRN | 0 | 0 |
| ITPKC | -1 | 0 |
| ERAL1 | 0 | 0 |
| P13K | 0 | 0 |
| IMPA1 | 0 | 0 |
| DHT | 0 | 0 |
| PR | 0 | 0 |
| NCOA3 | 0 | 0 |
| ESR1 | 1 | 0 |
| JUN | -1 | -1 |
| BRCA2 | 0 | 1 |
| RAD54L | 0 | 1 |
| BARD1 | 1 | 1 |
| SMCA4 | 0 | 0 |
| SP1 | 0 | 0 |
| MAPKs | 0 | 0 |
| P13 | 0 | 0 |
| ERK | 0 | 0 |
| P38 | 0 | 0 |
| ANDR | 0 | 0 |
| GDI | 0 | 0 |
| CASP8 | 0 | 0 |
| CASP3 | 1 | 1 |
| P85 | 0 | 0 |
| ABL1 | -1 | -1 |
| CBP | 0 | 0 |
| XRCC3 | 0 | 0 |
| TFPI | -1 | -1 |
| HDAC1/HDAC2 | 0 | 0 |
| P85A | 0 | 0 |
| GCR | 0 | 0 |
| PLK3 | 0 | 0 |
| NCOA2 | 0 | 1 |
| CREB | 0 | 0 |
| FOXO1 | -1 | 0 |
| IRS1 | -1 | -1 |
| CASP9 | 0 | -1 |
| NFKB1 | 0 | 0 |
| TSC2 | -1 | -1 |
| STK11 | -1 | -1 |
| MTOR | 0 | 0 |
| CDH1 | 0 | 0 |
| PHB | 0 | 0 |
| EP300 | 0 | 0 |
| CTNB1 | 0 | 0 |
| MPIP2 | 0 | 0 |
| GR | 0 | 0 |
| ANXA1 | -1 | -1 |
| AHR | 0 | 0 |
| RHO | -1 | -1 |
| CDK7 | 1 | 0 |
| AURKA | 0 | 0 |
| P65 | 0 | 0 |
| CYP19A1 | 0 | 0 |
| ODC1 | 0 | 0 |
| TCF/LEF | 0 | 0 |
| fra-1 | 0 | 0 |
| cyc-D | 0 | 0 |
| PPAR | 0 | 0 |
| UNC1 | 0 | 0 |
| M5 | 0 | 0 |
| ACHE | 0 | 0 |
| Gq11 | 0 | 0 |
| PLC | 0 | 0 |
| M2 | 0 | 0 |
| M4 | 0 | 0 |
| nAChR | 0 | 0 |
| AC | 0 | 0 |
| c-fos | 0 | 0 |
| PKA | 0 | 0 |
| CaMK | 0 | 0 |
| Fyn | -1 | 0 |
| FUMP | 0 | 0 |
| FUDP | 0 | 0 |
| RRM1 | 0 | 1 |
| FdUDP | 0 | 0 |
| FdUMP | 0 | 0 |
| TYMS | 1 | 1 |
| FdUTP | 0 | 0 |
| CytC | 0 | 0 |
| Apaf-1 | 0 | 0 |
| ESPG | 0 | 0 |
| TUBA | 0 | 0 |
| TUBB | -1 | -1 |
| GEF-H1 | 0 | 0 |
| ROCK | 0 | 0 |
| NOS | 0 | 0 |
| NO | 0 | 0 |
| sAC | 0 | 0 |
| sGC | 0 | 0 |
| PKG | 0 | 0 |
| nNOS | 0 | 0 |
| TUBB1 | 0 | 0 |
| TUBAL3 | 0 | 0 |
| TUBA1C | 0 | 0 |
| TUBB6 | 0 | 0 |
| TUBA3C | 0 | 0 |
| TUBB8 | 0 | 0 |
| TUBA1A | 0 | 0 |
| Raf | 0 | 0 |
| Sos | 0 | 0 |
| Grb2 | 1 | 1 |
| Shc | 0 | 0 |
| ERBB2 | 0 | 0 |
| AKT2 | 0 | 0 |
| AKT3 | 0 | 0 |
| TLK1/2 | 0 | 0 |
| CDC25C | 0 | 1 |
| CDC2 | 1 | 1 |
| ATAD5 | 0 | 0 |
| CDC25B | 0 | 0 |
| CDK1 | 0 | 0 |
| ERR1 | 0 | 0 |
| ERR2 | 0 | 0 |
| ERR3 | 0 | 0 |
| ESR2 | 0 | 0 |
| ESRRA | -1 | -1 |
| GRIA3 | 0 | 0 |
| LIG1/3 | 0 | 0 |
| NROB1 | 0 | 0 |
| GRB2 | 0 | 0 |
| SOS | 0 | 0 |
| Ras | 0 | 0 |
| PRKCA | 0 | 0 |
| CYP1A1 | 0 | 0 |
| MGMT | 0 | 0 |
| NCOA1 | 0 | -1 |
| CDK9 | 0 | 0 |
| NQO2 | 0 | 0 |
| MDR1 | 0 | 0 |
| ABCB1 | -1 | -1 |
| PIMP1 | 0 | 0 |
| DHFR | 0 | 0 |

## Supplemental table 5

Drugs’ Td scores in Breast Cancer: ER-positive subtype and ER-negative subtype

| Drug | Category | Td: ER-positive | Td: ER-negative |
| --- | --- | --- | --- |
| Anastrozole | D1 | 1.000 | -1.000 |
| Cycloheximide | D1 | 0.097 | 0.097 |
| Exemestane | D1 | 1.000 | -1.000 |
| Fluorouracil | D1 | -1.000 | 1.000 |
| Fluoxymesterone | D1 | -0.333 | -0.333 |
| Fulvestrant | D1 | 1.000 | 1.000 |
| Goserelin | D1 | 0.000 | 0.000 |
| Lapatinib | D1 | 0.000 | -1.000 |
| Letrozole | D1 | 1.000 | -1.000 |
| Megestrol | D1 | 0.000 | 0.000 |
| Methotrexate | D1 | 0.000 | 0.000 |
| Methyltestosterone | D1 | 0.000 | 0.000 |
| Miltefosine | D1 | -1.000 | -1.000 |
| Paclitaxel | D1 | 1.000 | 1.000 |
| Pamidronate | D1 | 1.000 | 1.000 |
| Raloxifene | D1 | 0.000 | 0.000 |
| Tamoxifen | D1 | 0.294 | 0.176 |
| Thiotepa | D1 | 1.000 | 1.000 |
| Trastuzumab Emtansine | D1 | -1.000 | -1.000 |
| Vinblastine | D1 | 0.000 | 0.000 |
| Avastin | D2 | -1.000 | -1.000 |
| Diethylstilbestrol | D2 | 0.000 | -0.333 |
| Dromostanolone Propionate | D2 | -1.000 | -1.000 |
| Ethyl Carbamate | D2 | -1.000 | -1.000 |
| Formestane | D2 | 0.000 | -1.000 |
| Hydralazine | D2 | 0.000 | 0.000 |
| Imetelstat | D2 | -0.333 | -1.000 |
| Ixabepilone | D2 | 1.000 | 1.000 |
| Tivozanib | D2 | -1.000 | -1.000 |
| Bleomycin | D3 | 0.600 | 0.600 |
| Corticosterone | D3 | 0.333 | 0.667 |
| Daunorubicin | D3 | -1.000 | 1.000 |
| Dexamethasone | D3 | -0.167 | 0.083 |
| Dihydrotestosterone | D3 | -0.714 | -0.714 |
| Donepezil | D3 | -0.333 | 1.000 |
| Erbitux | D3 | 1.000 | -1.000 |
| Ethinyl Estradiol | D3 | -1.000 | -1.000 |
| Flutamide | D3 | 1.000 | 0.000 |
| Hydrocortisone | D3 | -1.000 | 0.000 |
| Hydroxyurea | D3 | -0.077 | 0.077 |
| Lithium Chloride | D3 | -1.000 | -0.333 |
| Medrysone | D3 | 1.000 | -1.000 |
| Methyl Methanesulfonate | D3 | 0.143 | -0.143 |
| Methylprednisolone | D3 | 1.000 | -1.000 |
| Mitomycin | D3 | 0.500 | -0.167 |
| Nocodazole | D3 | -0.200 | 0.600 |
| Norethindrone | D3 | 1.000 | 1.000 |
| Oxandrolone | D3 | 0.000 | 0.000 |
| Plicamycin | D3 | 0.333 | 0.333 |
| Prednisolone | D3 | 1.000 | -1.000 |
| Prednisone | D3 | 1.000 | -1.000 |
| Progesterone | D3 | -1.000 | -1.000 |
| Testosterone | D3 | -1.000 | -1.000 |
| Trilostane | D3 | -1.000 | -1.000 |
| Vandetanib | D3 | 1.000 | -1.000 |

## Supplemental table 6

Protein-protein interactions in the Bladder Cancer system

| Interactor | Interactee | Mechanism of interaction |
| --- | --- | --- |
| MYC | MYC | inhibition |
| CDKN2A | MYC | activation |
| TGFB1 | MYC | inhibition |
| CCND1 | MYC | activation |
| CDKN1A | MYC | inhibition |
| IGF1 | MYC | activation |
| CDKN1B | MYC | inhibition |
| THBS1 | MYC | inhibition |
| CDK4 | MYC | activation |
| STK11 | MYC | inhibition |
| CCNB1 | MYC | activation |
| CSF3 | MYC | activation |
| ID2 | MYC | activation |
| LRIG1 | MYC | inhibition |
| CHKA | MYC | inhibition |
| CDKN2A | TBX3 | inhibition |
| CDH1 | TBX3 | inhibition |
| ERBB2 | ERBB2 | inhibition |
| CDH1 | ERBB2 | inhibition |
| CCND1 | ERBB2 | activation |
| CDKN1A | ERBB2 | activation |
| MMP2 | ERBB2 | activation |
| ESR1 | ERBB2 | inhibition |
| PTGS2 | ERBB2 | activation |
| CXCR4 | ERBB2 | activation |
| NFE2L2 | ERBB2 | activation |
| PRKCA | ERBB2 | activation |
| EGF | ERBB2 | activation |
| ILK | ERBB2 | activation |
| S100A4 | ERBB2 | activation |
| ELAVL1 | ERBB2 | activation |
| CSF3 | ERBB2 | activation |
| RECK | ERBB2 | inhibition |
| LRIG1 | ERBB2 | inhibition |
| MGAT5 | ERBB2 | activation |
| AKT1 | HIF1A | inhibition |
| TNF | HIF1A | activation |
| CDKN1A | HIF1A | activation |
| PTGS2 | HIF1A | activation |
| MTOR | HIF1A | inhibition |
| IGF1 | HIF1A | activation |
| SERPINE1 | HIF1A | inhibition |
| EDN1 | HIF1A | activation |
| AURKA | HIF1A | activation |
| HMOX1 | HIF1A | activation |
| IL18 | HIF1A | activation |
| PPARA | HIF1A | activation |
| CA9 | HIF1A | activation |
| GDF15 | HIF1A | activation |
| SOX9 | HIF1A | activation |
| HIPK2 | HIF1A | inhibition |
| RECK | HIF1A | inhibition |
| IL6 | CYP1A2 | inhibition |
| MYC | CDKN2A | activation |
| TBX3 | CDKN2A | inhibition |
| CCND1 | CDKN2A | inhibition |
| CDKN1A | CDKN2A | activation |
| MMP2 | CDKN2A | inhibition |
| JUN | CDKN2A | inhibition |
| CDK1 | CDKN2A | inhibition |
| ELAVL1 | CDKN2A | inhibition |
| CDK6 | CDKN2A | activation |
| E2F3 | CDKN2A | inhibition |
| EIF5A2 | CDKN2A | inhibition |
| HIF1A | AKT1 | inhibition |
| CDH1 | AKT1 | inhibition |
| TGFB1 | AKT1 | inhibition |
| CCND1 | AKT1 | activation |
| MMP2 | AKT1 | activation |
| PTGS2 | AKT1 | activation |
| BCL2 | AKT1 | activation |
| JAK2 | AKT1 | inhibition |
| IGF1R | AKT1 | activation |
| HMOX1 | AKT1 | activation |
| ICAM1 | AKT1 | activation |
| HRAS | AKT1 | inhibition |
| CDKN1B | AKT1 | inhibition |
| KDR | AKT1 | activation |
| FASLG | AKT1 | activation |
| ERBB3 | AKT1 | inhibition |
| SHH | AKT1 | activation |
| MME | AKT1 | inhibition |
| CCNB1 | AKT1 | inhibition |
| FOS | AKT1 | activation |
| GDF15 | AKT1 | activation |
| PIM1 | AKT1 | activation |
| ELK1 | AKT1 | inhibition |
| TGFB1 | APOE | activation |
| IL6 | APOE | inhibition |
| PTGS2 | APOE | activation |
| MMP1 | APOE | inhibition |
| MMP9 | MMP9 | activation |
| TNF | MMP9 | activation |
| TGFB1 | MMP9 | activation |
| IL6 | MMP9 | activation |
| CCL2 | MMP9 | activation |
| NOTCH1 | MMP9 | activation |
| MMP1 | MMP9 | activation |
| IL17A | MMP9 | activation |
| HRAS | MMP9 | activation |
| IL18 | MMP9 | activation |
| IL1A | MMP9 | activation |
| JUN | MMP9 | activation |
| MIF | MMP9 | activation |
| BSG | MMP9 | activation |
| CDH2 | MMP9 | activation |
| SMAD4 | MMP9 | activation |
| THBS1 | MMP9 | activation |
| KISS1 | MMP9 | inhibition |
| RPS6KB1 | MMP9 | activation |
| SMARCA4 | MMP9 | activation |
| CSF3 | MMP9 | activation |
| RECK | MMP9 | inhibition |
| HIF1A | TNF | activation |
| MMP9 | TNF | activation |
| TNF | TNF | activation |
| AR | TNF | activation |
| TGFB1 | TNF | activation |
| IL6 | TNF | activation |
| CCND1 | TNF | activation |
| CDKN1A | TNF | inhibition |
| MMP2 | TNF | activation |
| ESR1 | TNF | activation |
| PTGS2 | TNF | activation |
| IL10 | TNF | inhibition |
| COMT | TNF | activation |
| BCL2 | TNF | activation |
| CXCR4 | TNF | inhibition |
| IGF1 | TNF | activation |
| CCL2 | TNF | inhibition |
| SERPINE1 | TNF | activation |
| NOTCH1 | TNF | inhibition |
| HLA-A | TNF | activation |
| CAV1 | TNF | inhibition |
| MMP1 | TNF | activation |
| HMOX1 | TNF | inhibition |
| ICAM1 | TNF | activation |
| GSK3B | TNF | inhibition |
| MAPK14 | TNF | activation |
| RELA | TNF | activation |
| IL1A | TNF | activation |
| LGALS3 | TNF | activation |
| HMGB1 | TNF | activation |
| CD14 | TNF | activation |
| PLAU | TNF | activation |
| VEGFC | TNF | activation |
| GJA1 | TNF | inhibition |
| IL15 | TNF | activation |
| FOXO1 | TNF | activation |
| ANGPT2 | TNF | activation |
| THBD | TNF | inhibition |
| FOS | TNF | activation |
| GDF15 | TNF | activation |
| DCN | TNF | inhibition |
| IRF1 | TNF | inhibition |
| BBC3 | TNF | activation |
| AQP3 | TNF | inhibition |
| CXCL5 | TNF | activation |
| ST3GAL1 | TNF | activation |
| TBX3 | CDH1 | inhibition |
| ERBB2 | CDH1 | inhibition |
| AKT1 | CDH1 | inhibition |
| CDH1 | CDH1 | inhibition |
| AR | CDH1 | inhibition |
| TGFB1 | CDH1 | inhibition |
| CCND1 | CDH1 | activation |
| STAT3 | CDH1 | inhibition |
| NOTCH1 | CDH1 | inhibition |
| EGF | CDH1 | inhibition |
| ILK | CDH1 | inhibition |
| SDC1 | CDH1 | inhibition |
| CALR | CDH1 | inhibition |
| HMGA2 | CDH1 | inhibition |
| SMARCA4 | CDH1 | activation |
| SNAI2 | CDH1 | inhibition |
| ID2 | CDH1 | inhibition |
| IL12B | NFKB1 | inhibition |
| TNF | AR | activation |
| CDH1 | AR | inhibition |
| AR | AR | inhibition |
| IL6 | AR | activation |
| MMP1 | AR | inhibition |
| PA2G4 | AR | inhibition |
| MYC | TGFB1 | inhibition |
| AKT1 | TGFB1 | inhibition |
| APOE | TGFB1 | activation |
| MMP9 | TGFB1 | activation |
| TNF | TGFB1 | activation |
| CDH1 | TGFB1 | inhibition |
| IL6 | TGFB1 | activation |
| CCND1 | TGFB1 | inhibition |
| CDKN1A | TGFB1 | activation |
| MMP2 | TGFB1 | activation |
| PTEN | TGFB1 | inhibition |
| IL10 | TGFB1 | inhibition |
| BCL2 | TGFB1 | inhibition |
| CXCR4 | TGFB1 | activation |
| SERPINE1 | TGFB1 | activation |
| CAV1 | TGFB1 | inhibition |
| MMP1 | TGFB1 | inhibition |
| HMOX1 | TGFB1 | activation |
| ICAM1 | TGFB1 | inhibition |
| JUN | TGFB1 | activation |
| PDGFRA | TGFB1 | inhibition |
| EGR1 | TGFB1 | activation |
| TGFBR1 | TGFB1 | inhibition |
| DCN | TGFB1 | inhibition |
| SPHK1 | TGFB1 | activation |
| ID2 | TGFB1 | activation |
| ADAM9 | TGFB1 | activation |
| CYP1A2 | IL6 | inhibition |
| APOE | IL6 | inhibition |
| MMP9 | IL6 | activation |
| TNF | IL6 | activation |
| AR | IL6 | activation |
| TGFB1 | IL6 | activation |
| IL6 | IL6 | activation |
| CDKN1A | IL6 | activation |
| STAT3 | IL6 | activation |
| ESR1 | IL6 | inhibition |
| PTGS2 | IL6 | activation |
| CXCR4 | IL6 | activation |
| MET | IL6 | activation |
| CCL2 | IL6 | activation |
| MAPK1 | IL6 | activation |
| SERPINE1 | IL6 | activation |
| EDN1 | IL6 | activation |
| MMP1 | IL6 | activation |
| HMOX1 | IL6 | activation |
| IL17A | IL6 | activation |
| ICAM1 | IL6 | activation |
| HRAS | IL6 | inhibition |
| IL18 | IL6 | activation |
| PRKCA | IL6 | activation |
| KDR | IL6 | activation |
| IL1A | IL6 | activation |
| EGF | IL6 | activation |
| JUN | IL6 | activation |
| GPX1 | IL6 | inhibition |
| DNMT1 | IL6 | activation |
| IL15 | IL6 | activation |
| ALOX5 | IL6 | activation |
| CTSB | IL6 | activation |
| CXCL1 | IL6 | activation |
| CD163 | IL6 | activation |
| SENP1 | IL6 | activation |
| REG3A | IL6 | activation |
| NNAT | IL6 | activation |
| MYC | CCND1 | activation |
| ERBB2 | CCND1 | activation |
| CDKN2A | CCND1 | inhibition |
| AKT1 | CCND1 | activation |
| TNF | CCND1 | activation |
| CDH1 | CCND1 | activation |
| TGFB1 | CCND1 | inhibition |
| STAT3 | CCND1 | inhibition |
| PTEN | CCND1 | inhibition |
| ESR1 | CCND1 | inhibition |
| MTOR | CCND1 | activation |
| IGF1 | CCND1 | activation |
| EDN1 | CCND1 | activation |
| IL4 | CCND1 | inhibition |
| CAV1 | CCND1 | inhibition |
| MAPK14 | CCND1 | inhibition |
| EGF | CCND1 | activation |
| LGALS3 | CCND1 | activation |
| JUN | CCND1 | activation |
| ILK | CCND1 | activation |
| CDH2 | CCND1 | inhibition |
| SMAD4 | CCND1 | inhibition |
| SDC1 | CCND1 | inhibition |
| CREB1 | CCND1 | activation |
| THBS1 | CCND1 | inhibition |
| FHIT | CCND1 | inhibition |
| SHH | CCND1 | activation |
| FOXO1 | CCND1 | inhibition |
| EGR1 | CCND1 | activation |
| CDK4 | CCND1 | activation |
| STK11 | CCND1 | inhibition |
| SFRP1 | CCND1 | inhibition |
| KLF5 | CCND1 | activation |
| HOXA9 | CCND1 | activation |
| HK2 | CCND1 | inhibition |
| BTG2 | CCND1 | inhibition |
| RALA | CCND1 | activation |
| SENP1 | CCND1 | activation |
| MYC | CDKN1A | inhibition |
| ERBB2 | CDKN1A | activation |
| HIF1A | CDKN1A | activation |
| CDKN2A | CDKN1A | activation |
| TNF | CDKN1A | inhibition |
| TGFB1 | CDKN1A | activation |
| IL6 | CDKN1A | activation |
| CDKN1A | CDKN1A | inhibition |
| STAT3 | CDKN1A | inhibition |
| IL10 | CDKN1A | activation |
| NOTCH1 | CDKN1A | inhibition |
| EGF | CDKN1A | activation |
| RUNX3 | CDKN1A | activation |
| CDK4 | CDKN1A | activation |
| STK11 | CDKN1A | activation |
| ALOX5 | CDKN1A | activation |
| SOX9 | CDKN1A | activation |
| DCN | CDKN1A | activation |
| IRF1 | CDKN1A | activation |
| ELK1 | CDKN1A | inhibition |
| ID2 | CDKN1A | inhibition |
| SENP1 | CDKN1A | activation |
| TBX2 | CDKN1A | inhibition |
| IGF1 | EZR | inhibition |
| FOS | EZR | activation |
| ERBB2 | MMP2 | activation |
| CDKN2A | MMP2 | inhibition |
| AKT1 | MMP2 | activation |
| TNF | MMP2 | activation |
| TGFB1 | MMP2 | activation |
| PTEN | MMP2 | inhibition |
| IL10 | MMP2 | inhibition |
| CCL2 | MMP2 | activation |
| EDN1 | MMP2 | activation |
| EGF | MMP2 | activation |
| SLC2A1 | MMP2 | activation |
| FOXM1 | MMP2 | activation |
| YBX1 | MMP2 | activation |
| SMARCA4 | MMP2 | activation |
| CD9 | MMP2 | activation |
| NNMT | MMP2 | activation |
| CDH1 | STAT3 | inhibition |
| IL6 | STAT3 | activation |
| CCND1 | STAT3 | inhibition |
| CDKN1A | STAT3 | inhibition |
| STAT3 | STAT3 | inhibition |
| PTGS2 | STAT3 | activation |
| IL10 | STAT3 | activation |
| HMOX1 | STAT3 | inhibition |
| IL17A | STAT3 | activation |
| HRAS | STAT3 | inhibition |
| EGF | STAT3 | activation |
| BCL2L1 | STAT3 | inhibition |
| MMP7 | STAT3 | inhibition |
| IL5 | STAT3 | activation |
| KLF5 | STAT3 | activation |
| NNMT | STAT3 | activation |
| TGFB1 | PTEN | inhibition |
| CCND1 | PTEN | inhibition |
| MMP2 | PTEN | inhibition |
| PTEN | PTEN | activation |
| MTOR | PTEN | inhibition |
| NOTCH1 | PTEN | inhibition |
| IL17A | PTEN | inhibition |
| IL18 | PTEN | activation |
| CDKN1B | PTEN | activation |
| CD274 | PTEN | activation |
| JUN | PTEN | inhibition |
| EGR1 | PTEN | activation |
| TGFBR1 | PTEN | inhibition |
| FOS | PTEN | inhibition |
| MAP2K4 | PTEN | inhibition |
| ERBB2 | ESR1 | inhibition |
| TNF | ESR1 | activation |
| IL6 | ESR1 | inhibition |
| CCND1 | ESR1 | inhibition |
| ESR1 | ESR1 | inhibition |
| PTGS2 | ESR1 | inhibition |
| IGF1 | ESR1 | activation |
| RB1 | ESR1 | activation |
| JAK2 | ESR1 | activation |
| CAV1 | ESR1 | inhibition |
| MMP1 | ESR1 | activation |
| FASLG | ESR1 | activation |
| HMGA1 | ESR1 | activation |
| ERBB2 | PTGS2 | activation |
| HIF1A | PTGS2 | activation |
| AKT1 | PTGS2 | activation |
| APOE | PTGS2 | activation |
| TNF | PTGS2 | activation |
| IL6 | PTGS2 | activation |
| STAT3 | PTGS2 | activation |
| ESR1 | PTGS2 | inhibition |
| IL10 | PTGS2 | inhibition |
| ABCB1 | PTGS2 | activation |
| APOA1 | PTGS2 | activation |
| MAPK1 | PTGS2 | activation |
| IGF1R | PTGS2 | inhibition |
| IL4 | PTGS2 | inhibition |
| NOTCH1 | PTGS2 | activation |
| PIK3CA | PTGS2 | inhibition |
| HRAS | PTGS2 | activation |
| MAPK14 | PTGS2 | inhibition |
| CYP2E1 | PTGS2 | activation |
| IL1A | PTGS2 | activation |
| EGF | PTGS2 | activation |
| APC | PTGS2 | inhibition |
| IL13 | PTGS2 | activation |
| CYP1B1 | PTGS2 | activation |
| ILK | PTGS2 | activation |
| VEGFC | PTGS2 | activation |
| CREB1 | PTGS2 | inhibition |
| FYN | PTGS2 | inhibition |
| EGR1 | PTGS2 | activation |
| STK11 | PTGS2 | inhibition |
| PIK3CG | PTGS2 | inhibition |
| TRPV1 | PTGS2 | activation |
| ELAVL1 | PTGS2 | activation |
| FOS | PTGS2 | activation |
| RARB | PTGS2 | inhibition |
| BDKRB2 | PTGS2 | activation |
| NCOR1 | PTGS2 | inhibition |
| MMP8 | PTGS2 | activation |
| MAP2K4 | PTGS2 | activation |
| GDF9 | PTGS2 | activation |
| MAP2K6 | PTGS2 | activation |
| TNF | IL10 | inhibition |
| TGFB1 | IL10 | inhibition |
| CDKN1A | IL10 | activation |
| MMP2 | IL10 | inhibition |
| STAT3 | IL10 | activation |
| PTGS2 | IL10 | inhibition |
| IL10 | IL10 | activation |
| BCL2 | IL10 | activation |
| CXCR4 | IL10 | activation |
| MAPK1 | IL10 | activation |
| IL4 | IL10 | activation |
| HMOX1 | IL10 | activation |
| ICAM1 | IL10 | inhibition |
| HLA-G | IL10 | activation |
| CD14 | IL10 | activation |
| FASLG | IL10 | activation |
| IL12B | IL10 | inhibition |
| DCN | IL10 | activation |
| CD163 | IL10 | activation |
| CCL18 | IL10 | activation |
| ACE | ACE | activation |
| KRAS | ACE | activation |
| SERPINE1 | ACE | activation |
| HRAS | ACE | activation |
| CDKN1B | ACE | inhibition |
| PLAU | ACE | inhibition |
| NRAS | ACE | activation |
| FOS | ACE | activation |
| TNF | COMT | activation |
| AKT1 | BCL2 | activation |
| TNF | BCL2 | activation |
| TGFB1 | BCL2 | inhibition |
| IL10 | BCL2 | activation |
| EDN1 | BCL2 | activation |
| IL18 | BCL2 | inhibition |
| BCL2L1 | BCL2 | activation |
| CREB1 | BCL2 | activation |
| GLI1 | BCL2 | activation |
| SATB1 | BCL2 | activation |
| HIF1A | MTOR | inhibition |
| CCND1 | MTOR | activation |
| PTEN | MTOR | inhibition |
| IGF1R | MTOR | activation |
| ID2 | MTOR | activation |
| PTGS2 | ABCB1 | activation |
| EDN1 | ABCB1 | activation |
| PIK3CA | ABCB1 | activation |
| EGR1 | ABCB1 | activation |
| PIK3CG | ABCB1 | activation |
| YBX1 | ABCB1 | activation |
| ACE | KRAS | activation |
| FOS | KRAS | activation |
| NOTCH1 | JAG1 | activation |
| RELA | JAG1 | activation |
| ERBB2 | CXCR4 | activation |
| TNF | CXCR4 | inhibition |
| TGFB1 | CXCR4 | activation |
| IL6 | CXCR4 | activation |
| IL10 | CXCR4 | activation |
| CCL2 | CXCR4 | activation |
| IL4 | CXCR4 | activation |
| NOTCH1 | CXCR4 | activation |
| EGF | CXCR4 | activation |
| SELL | CXCR4 | activation |
| DLL4 | CXCR4 | inhibition |
| CYP1A1 | CYP1A1 | activation |
| THBS1 | CYP1A1 | activation |
| MYC | IGF1 | activation |
| HIF1A | IGF1 | activation |
| TNF | IGF1 | activation |
| CCND1 | IGF1 | activation |
| EZR | IGF1 | inhibition |
| ESR1 | IGF1 | activation |
| IGF1R | IGF1 | inhibition |
| CAV1 | IGF1 | activation |
| ICAM1 | IGF1 | activation |
| HRAS | IGF1 | activation |
| PARP1 | IGF1 | inhibition |
| TWIST1 | IGF1 | activation |
| EGF | IGF1 | activation |
| JUN | IGF1 | activation |
| CREB1 | IGF1 | activation |
| FOS | IGF1 | activation |
| BIRC3 | IGF1 | activation |
| MDM4 | IGF1 | activation |
| ID2 | IGF1 | activation |
| ESR1 | RB1 | activation |
| NFE2L2 | NQO1 | activation |
| PTGS2 | APOA1 | activation |
| IL6 | MET | activation |
| MET | MET | inhibition |
| AQP3 | MET | activation |
| MMP9 | CCL2 | activation |
| TNF | CCL2 | inhibition |
| IL6 | CCL2 | activation |
| MMP2 | CCL2 | activation |
| CXCR4 | CCL2 | activation |
| JAK2 | CCL2 | inhibition |
| MMP1 | CCL2 | activation |
| HMOX1 | CCL2 | inhibition |
| IL17A | CCL2 | activation |
| ICAM1 | CCL2 | activation |
| MAPK14 | CCL2 | activation |
| IL18 | CCL2 | activation |
| PARP1 | CCL2 | activation |
| PLAU | CCL2 | inhibition |
| FASLG | CCL2 | activation |
| PPARA | CCL2 | inhibition |
| SPHK1 | CCL2 | inhibition |
| AKT1 | JAK2 | inhibition |
| ESR1 | JAK2 | activation |
| CCL2 | JAK2 | inhibition |
| BCL2L1 | JAK2 | activation |
| IL6 | MAPK1 | activation |
| PTGS2 | MAPK1 | activation |
| IL10 | MAPK1 | activation |
| HIF1A | SERPINE1 | inhibition |
| TNF | SERPINE1 | activation |
| TGFB1 | SERPINE1 | activation |
| IL6 | SERPINE1 | activation |
| ACE | SERPINE1 | activation |
| SERPINE1 | SERPINE1 | activation |
| PLAU | SERPINE1 | activation |
| SMAD4 | SERPINE1 | inhibition |
| FOXO1 | SERPINE1 | inhibition |
| BIRC3 | SERPINE1 | activation |
| AKT1 | IGF1R | activation |
| PTGS2 | IGF1R | inhibition |
| MTOR | IGF1R | activation |
| IGF1 | IGF1R | inhibition |
| IGF1R | IGF1R | activation |
| VEGFC | IGF1R | activation |
| HOXA9 | IGF1R | activation |
| HIF1A | EDN1 | activation |
| IL6 | EDN1 | activation |
| CCND1 | EDN1 | activation |
| MMP2 | EDN1 | activation |
| BCL2 | EDN1 | activation |
| ABCB1 | EDN1 | activation |
| EDN1 | EDN1 | activation |
| ICAM1 | EDN1 | activation |
| IL18 | EDN1 | activation |
| ILK | EDN1 | activation |
| GJA1 | EDN1 | inhibition |
| PPARA | EDN1 | inhibition |
| FOS | EDN1 | activation |
| GATA2 | EDN1 | activation |
| KLF5 | EDN1 | activation |
| CCND1 | IL4 | inhibition |
| PTGS2 | IL4 | inhibition |
| IL10 | IL4 | activation |
| CXCR4 | IL4 | activation |
| IL4 | IL4 | activation |
| MMP1 | IL4 | activation |
| IL1RN | IL4 | activation |
| PLAU | IL4 | activation |
| FASLG | IL4 | inhibition |
| IL13 | IL4 | activation |
| IL15 | IL4 | activation |
| MMP9 | NOTCH1 | activation |
| TNF | NOTCH1 | inhibition |
| CDH1 | NOTCH1 | inhibition |
| CDKN1A | NOTCH1 | inhibition |
| PTEN | NOTCH1 | inhibition |
| PTGS2 | NOTCH1 | activation |
| JAG1 | NOTCH1 | activation |
| CXCR4 | NOTCH1 | activation |
| NOTCH1 | NOTCH1 | activation |
| ERBB4 | NOTCH1 | activation |
| SOX9 | NOTCH1 | activation |
| FOSL1 | NOTCH1 | activation |
| DLL4 | NOTCH1 | activation |
| HIF1A | AURKA | activation |
| GATA3 | AURKA | activation |
| PLAU | PON1 | inhibition |
| SREBF2 | PON1 | activation |
| TNF | HLA-A | activation |
| TNF | CAV1 | inhibition |
| TGFB1 | CAV1 | inhibition |
| CCND1 | CAV1 | inhibition |
| ESR1 | CAV1 | inhibition |
| IGF1 | CAV1 | activation |
| APOE | MMP1 | inhibition |
| MMP9 | MMP1 | activation |
| TNF | MMP1 | activation |
| AR | MMP1 | inhibition |
| TGFB1 | MMP1 | inhibition |
| IL6 | MMP1 | activation |
| ESR1 | MMP1 | activation |
| CCL2 | MMP1 | activation |
| IL4 | MMP1 | activation |
| MAPK14 | MMP1 | inhibition |
| IL1A | MMP1 | activation |
| EGF | MMP1 | activation |
| JUN | MMP1 | activation |
| BSG | MMP1 | activation |
| TRPV1 | MMP1 | activation |
| CSF3 | MMP1 | activation |
| SFN | MMP1 | activation |
| NR4A2 | MMP1 | inhibition |
| FOSL1 | MMP1 | activation |
| PTGS2 | PIK3CA | inhibition |
| ABCB1 | PIK3CA | activation |
| PIK3CA | PIK3CA | activation |
| ISG15 | PIK3CA | inhibition |
| HIF1A | HMOX1 | activation |
| AKT1 | HMOX1 | activation |
| TNF | HMOX1 | inhibition |
| TGFB1 | HMOX1 | activation |
| IL6 | HMOX1 | activation |
| STAT3 | HMOX1 | inhibition |
| IL10 | HMOX1 | activation |
| CCL2 | HMOX1 | inhibition |
| HMOX1 | HMOX1 | activation |
| IL18 | HMOX1 | inhibition |
| NFE2L2 | HMOX1 | activation |
| CDKN1B | HMOX1 | activation |
| CYP2E1 | HMOX1 | activation |
| JUN | HMOX1 | activation |
| ELAVL1 | HMOX1 | activation |
| CD163 | HMOX1 | activation |
| MMP9 | IL17A | activation |
| IL6 | IL17A | activation |
| STAT3 | IL17A | activation |
| PTEN | IL17A | inhibition |
| CCL2 | IL17A | activation |
| JUN | IL17A | activation |
| MMP3 | IL17A | activation |
| NFKBIA | IL17A | activation |
| DEFB4A | IL17A | activation |
| CASP3 | CASP3 | activation |
| FASLG | CASP3 | activation |
| DICER1 | CASP3 | inhibition |
| SFRP1 | CASP3 | inhibition |
| AKT1 | ICAM1 | activation |
| TNF | ICAM1 | activation |
| TGFB1 | ICAM1 | inhibition |
| IL6 | ICAM1 | activation |
| IL10 | ICAM1 | inhibition |
| IGF1 | ICAM1 | activation |
| CCL2 | ICAM1 | activation |
| EDN1 | ICAM1 | activation |
| ICAM1 | ICAM1 | activation |
| IL18 | ICAM1 | activation |
| PARP1 | ICAM1 | activation |
| IL1A | ICAM1 | activation |
| JUN | ICAM1 | activation |
| HMGB1 | ICAM1 | activation |
| APOC3 | ICAM1 | activation |
| NFKBIA | ICAM1 | activation |
| IL15 | ICAM1 | inhibition |
| MME | ICAM1 | activation |
| IL5 | ICAM1 | activation |
| CD80 | ICAM1 | inhibition |
| AKT1 | HRAS | inhibition |
| MMP9 | HRAS | activation |
| IL6 | HRAS | inhibition |
| STAT3 | HRAS | inhibition |
| PTGS2 | HRAS | activation |
| ACE | HRAS | activation |
| IGF1 | HRAS | activation |
| NFE2L2 | HRAS | inhibition |
| JUN | HRAS | activation |
| ERCC1 | HRAS | activation |
| GJA1 | HRAS | activation |
| MTDH | HRAS | activation |
| CCNB1 | HRAS | activation |
| FOS | HRAS | activation |
| TNF | GSK3B | inhibition |
| TNF | MAPK14 | activation |
| CCND1 | MAPK14 | inhibition |
| PTGS2 | MAPK14 | inhibition |
| CCL2 | MAPK14 | activation |
| MMP1 | MAPK14 | inhibition |
| HIF1A | IL18 | activation |
| MMP9 | IL18 | activation |
| IL6 | IL18 | activation |
| PTEN | IL18 | activation |
| BCL2 | IL18 | inhibition |
| CCL2 | IL18 | activation |
| EDN1 | IL18 | activation |
| HMOX1 | IL18 | inhibition |
| ICAM1 | IL18 | activation |
| BCL2L1 | IL18 | activation |
| IL12B | IL18 | activation |
| CHI3L1 | IL18 | activation |
| ERBB2 | NFE2L2 | activation |
| NQO1 | NFE2L2 | activation |
| HMOX1 | NFE2L2 | activation |
| HRAS | NFE2L2 | inhibition |
| SMARCA4 | NFE2L2 | activation |
| TNF | RELA | activation |
| JAG1 | RELA | activation |
| JUN | RELA | activation |
| FASLG | RELA | activation |
| BCL2L1 | RELA | inhibition |
| MYC | CDKN1B | inhibition |
| AKT1 | CDKN1B | inhibition |
| PTEN | CDKN1B | activation |
| ACE | CDKN1B | inhibition |
| HMOX1 | CDKN1B | activation |
| CDKN1B | CDKN1B | inhibition |
| EGF | CDKN1B | activation |
| JUN | CDKN1B | activation |
| TIMP2 | CDKN1B | activation |
| IL10 | HLA-G | activation |
| MLH1 | MLH1 | inhibition |
| PTGS2 | CYP2E1 | activation |
| HMOX1 | CYP2E1 | activation |
| IGF1 | PARP1 | inhibition |
| CCL2 | PARP1 | activation |
| ICAM1 | PARP1 | activation |
| PARP1 | PARP1 | activation |
| TIMP2 | PARP1 | inhibition |
| ERBB2 | PRKCA | activation |
| IL6 | PRKCA | activation |
| IL4 | IL1RN | activation |
| MMP3 | IL1RN | activation |
| AKT1 | KDR | activation |
| IL6 | KDR | activation |
| PPARA | KDR | inhibition |
| IGF1 | TWIST1 | activation |
| AGTR1 | AGTR1 | inhibition |
| FOS | AGTR1 | activation |
| MMP9 | IL1A | activation |
| TNF | IL1A | activation |
| IL6 | IL1A | activation |
| PTGS2 | IL1A | activation |
| MMP1 | IL1A | activation |
| ICAM1 | IL1A | activation |
| IL1A | IL1A | activation |
| MMP3 | IL1A | activation |
| PLAU | IL1A | activation |
| DEFB4A | IL1A | activation |
| CXCL1 | IL1A | activation |
| SPHK1 | IL1A | activation |
| LRAT | IL1A | inhibition |
| ERBB2 | EGF | activation |
| CDH1 | EGF | inhibition |
| IL6 | EGF | activation |
| CCND1 | EGF | activation |
| CDKN1A | EGF | activation |
| MMP2 | EGF | activation |
| STAT3 | EGF | activation |
| PTGS2 | EGF | activation |
| CXCR4 | EGF | activation |
| IGF1 | EGF | activation |
| MMP1 | EGF | activation |
| CDKN1B | EGF | activation |
| JUN | EGF | activation |
| PLAU | EGF | activation |
| BSG | EGF | inhibition |
| ILK | EGF | activation |
| RUNX3 | EGF | activation |
| BCL2L1 | EGF | activation |
| GJA1 | EGF | inhibition |
| MMP7 | EGF | activation |
| SDC1 | EGF | activation |
| THBS1 | EGF | activation |
| TGFBR2 | EGF | activation |
| EGR1 | EGF | activation |
| FOS | EGF | activation |
| DCN | EGF | inhibition |
| SPHK1 | EGF | activation |
| HSD3B2 | EGF | activation |
| TNF | LGALS3 | activation |
| CCND1 | LGALS3 | activation |
| LGALS3 | LGALS3 | inhibition |
| PTEN | CD274 | activation |
| CDKN2A | JUN | inhibition |
| MMP9 | JUN | activation |
| TGFB1 | JUN | activation |
| IL6 | JUN | activation |
| CCND1 | JUN | activation |
| PTEN | JUN | inhibition |
| IGF1 | JUN | activation |
| MMP1 | JUN | activation |
| HMOX1 | JUN | activation |
| IL17A | JUN | activation |
| ICAM1 | JUN | activation |
| HRAS | JUN | activation |
| RELA | JUN | activation |
| CDKN1B | JUN | activation |
| EGF | JUN | activation |
| JUN | JUN | activation |
| FASLG | JUN | activation |
| GJA1 | JUN | activation |
| CREB1 | JUN | activation |
| THBS1 | JUN | inhibition |
| CSF3 | JUN | activation |
| CCNA1 | JUN | activation |
| PTGS2 | APC | inhibition |
| TNF | HMGB1 | activation |
| ICAM1 | HMGB1 | activation |
| HRAS | ERCC1 | activation |
| ERCC1 | ERCC1 | inhibition |
| MSH2 | MSH2 | inhibition |
| MMP9 | MIF | activation |
| PSEN1 | PSEN1 | activation |
| ETS2 | PSEN1 | activation |
| TNF | CD14 | activation |
| IL10 | CD14 | activation |
| IL17A | MMP3 | activation |
| IL1RN | MMP3 | activation |
| IL1A | MMP3 | activation |
| PLCG1 | MMP3 | activation |
| TNF | PLAU | activation |
| ACE | PLAU | inhibition |
| CCL2 | PLAU | inhibition |
| SERPINE1 | PLAU | activation |
| IL4 | PLAU | activation |
| PON1 | PLAU | inhibition |
| IL1A | PLAU | activation |
| EGF | PLAU | activation |
| THBS1 | PLAU | activation |
| MAPKAPK2 | PLAU | inhibition |
| MAP2K6 | PLAU | activation |
| AKT1 | FASLG | activation |
| ESR1 | FASLG | activation |
| IL10 | FASLG | activation |
| CCL2 | FASLG | activation |
| IL4 | FASLG | inhibition |
| CASP3 | FASLG | activation |
| RELA | FASLG | activation |
| JUN | FASLG | activation |
| FASLG | FASLG | activation |
| CSF3 | FASLG | inhibition |
| PTGS2 | IL13 | activation |
| IL4 | IL13 | activation |
| GATA3 | IL13 | inhibition |
| PTGS2 | CYP1B1 | activation |
| CYP1B1 | CYP1B1 | activation |
| MMP9 | BSG | activation |
| MMP1 | BSG | activation |
| EGF | BSG | inhibition |
| ERBB2 | ILK | activation |
| CDH1 | ILK | inhibition |
| CCND1 | ILK | activation |
| PTGS2 | ILK | activation |
| EDN1 | ILK | activation |
| EGF | ILK | activation |
| KLF5 | ILK | activation |
| MMP2 | SLC2A1 | activation |
| TNF | VEGFC | activation |
| PTGS2 | VEGFC | activation |
| IGF1R | VEGFC | activation |
| FOXO1 | VEGFC | activation |
| CDKN1A | RUNX3 | activation |
| EGF | RUNX3 | activation |
| STAT3 | BCL2L1 | inhibition |
| BCL2 | BCL2L1 | activation |
| JAK2 | BCL2L1 | activation |
| IL18 | BCL2L1 | activation |
| RELA | BCL2L1 | inhibition |
| EGF | BCL2L1 | activation |
| BCL2L1 | BCL2L1 | inhibition |
| MMP9 | CDH2 | activation |
| CCND1 | CDH2 | inhibition |
| CDH2 | CDH2 | activation |
| SOX9 | CDH2 | activation |
| MMP9 | SMAD4 | activation |
| CCND1 | SMAD4 | inhibition |
| SERPINE1 | SMAD4 | inhibition |
| CDKN2A | CDK1 | inhibition |
| IL6 | GPX1 | inhibition |
| TNF | GJA1 | inhibition |
| EDN1 | GJA1 | inhibition |
| HRAS | GJA1 | activation |
| EGF | GJA1 | inhibition |
| JUN | GJA1 | activation |
| GJA1 | GJA1 | activation |
| TBX2 | GJA1 | inhibition |
| AKT1 | ERBB3 | inhibition |
| NFKB1 | IL12B | inhibition |
| IL10 | IL12B | inhibition |
| IL18 | IL12B | activation |
| IRF1 | IL12B | activation |
| STAT3 | MMP7 | inhibition |
| EGF | MMP7 | activation |
| HIF1A | PPARA | activation |
| CCL2 | PPARA | inhibition |
| EDN1 | PPARA | inhibition |
| KDR | PPARA | inhibition |
| APOC3 | PPARA | inhibition |
| NFKBIA | PPARA | activation |
| IL6 | DNMT1 | activation |
| FOS | DNMT1 | activation |
| CDH1 | SDC1 | inhibition |
| CCND1 | SDC1 | inhibition |
| EGF | SDC1 | activation |
| HPSE | SDC1 | activation |
| ID2 | SDC1 | activation |
| CCND1 | CREB1 | activation |
| PTGS2 | CREB1 | inhibition |
| BCL2 | CREB1 | activation |
| IGF1 | CREB1 | activation |
| JUN | CREB1 | activation |
| EGR1 | CREB1 | inhibition |
| ICAM1 | APOC3 | activation |
| PPARA | APOC3 | inhibition |
| FOXO1 | APOC3 | activation |
| MYC | THBS1 | inhibition |
| MMP9 | THBS1 | activation |
| CCND1 | THBS1 | inhibition |
| CYP1A1 | THBS1 | activation |
| EGF | THBS1 | activation |
| JUN | THBS1 | inhibition |
| PLAU | THBS1 | activation |
| DCN | THBS1 | activation |
| RRM2 | THBS1 | inhibition |
| MAP2K6 | THBS1 | activation |
| CCND1 | FHIT | inhibition |
| FHIT | FHIT | activation |
| MMP9 | KISS1 | inhibition |
| KISS1 | KISS1 | activation |
| AKT1 | SHH | activation |
| CCND1 | SHH | activation |
| SHH | SHH | activation |
| GATA3 | SHH | activation |
| SOX9 | SHH | activation |
| GLI1 | SHH | activation |
| AXL | SHH | activation |
| SFRP1 | SHH | activation |
| IL18 | CHI3L1 | activation |
| IL17A | NFKBIA | activation |
| ICAM1 | NFKBIA | activation |
| PPARA | NFKBIA | activation |
| EGF | TGFBR2 | activation |
| EGR1 | TGFBR2 | inhibition |
| TGFB1 | PDGFRA | inhibition |
| PTGS2 | FYN | inhibition |
| FOS | FYN | activation |
| CASP3 | DICER1 | inhibition |
| SOX4 | DICER1 | activation |
| TNF | IL15 | activation |
| IL6 | IL15 | activation |
| IL4 | IL15 | activation |
| ICAM1 | IL15 | inhibition |
| BECN1 | BECN1 | inhibition |
| TNF | FOXO1 | activation |
| CCND1 | FOXO1 | inhibition |
| SERPINE1 | FOXO1 | inhibition |
| VEGFC | FOXO1 | activation |
| APOC3 | FOXO1 | activation |
| FOXO1 | FOXO1 | inhibition |
| SELL | FOXO1 | activation |
| PCDH8 | CCR2 | inhibition |
| HRAS | MTDH | activation |
| TGFB1 | EGR1 | activation |
| CCND1 | EGR1 | activation |
| PTEN | EGR1 | activation |
| PTGS2 | EGR1 | activation |
| ABCB1 | EGR1 | activation |
| EGF | EGR1 | activation |
| CREB1 | EGR1 | inhibition |
| TGFBR2 | EGR1 | inhibition |
| EGR1 | EGR1 | inhibition |
| ALOX5 | EGR1 | activation |
| CSF3 | EGR1 | activation |
| ELK1 | EGR1 | inhibition |
| ID2 | EGR1 | activation |
| CXCL5 | EGR1 | activation |
| CDKN1B | TIMP2 | activation |
| PARP1 | TIMP2 | inhibition |
| TIMP2 | TIMP2 | activation |
| RECK | TIMP2 | activation |
| AKT1 | MME | inhibition |
| ICAM1 | MME | activation |
| MMP2 | FOXM1 | activation |
| FOXM1 | FOXM1 | activation |
| CCNB1 | FOXM1 | activation |
| GLI1 | FOXM1 | activation |
| TNF | ANGPT2 | activation |
| GATA2 | ANGPT2 | activation |
| SDC1 | HPSE | activation |
| TGFB1 | TGFBR1 | inhibition |
| PTEN | TGFBR1 | inhibition |
| MYC | CDK4 | activation |
| CCND1 | CDK4 | activation |
| CDKN1A | CDK4 | activation |
| FOS | CDK4 | inhibition |
| SFRP1 | CDK4 | activation |
| MYC | STK11 | inhibition |
| CCND1 | STK11 | inhibition |
| CDKN1A | STK11 | activation |
| PTGS2 | STK11 | inhibition |
| STK11 | STK11 | activation |
| CDH1 | CALR | inhibition |
| NOTCH1 | ERBB4 | activation |
| ERBB4 | ERBB4 | inhibition |
| ACE | NRAS | activation |
| FOS | NRAS | activation |
| PTGS2 | PIK3CG | inhibition |
| ABCB1 | PIK3CG | activation |
| ISG15 | PIK3CG | inhibition |
| MYC | CCNB1 | activation |
| AKT1 | CCNB1 | inhibition |
| HRAS | CCNB1 | activation |
| FOXM1 | CCNB1 | activation |
| YBX1 | CCNB1 | activation |
| FOS | CCNB1 | activation |
| HIF1A | CA9 | activation |
| ERBB2 | S100A4 | activation |
| PAK1 | PAK1 | inhibition |
| TNF | THBD | inhibition |
| MMP2 | YBX1 | activation |
| ABCB1 | YBX1 | activation |
| CCNB1 | YBX1 | activation |
| YBX1 | YBX1 | inhibition |
| AURKA | GATA3 | activation |
| IL13 | GATA3 | inhibition |
| SHH | GATA3 | activation |
| GATA2 | PAX6 | inhibition |
| PTGS2 | TRPV1 | activation |
| MMP1 | TRPV1 | activation |
| ERBB2 | ELAVL1 | activation |
| CDKN2A | ELAVL1 | inhibition |
| PTGS2 | ELAVL1 | activation |
| HMOX1 | ELAVL1 | activation |
| GDF15 | ELAVL1 | activation |
| MMP9 | RPS6KB1 | activation |
| AKT1 | FOS | activation |
| TNF | FOS | activation |
| EZR | FOS | activation |
| PTEN | FOS | inhibition |
| PTGS2 | FOS | activation |
| ACE | FOS | activation |
| KRAS | FOS | activation |
| IGF1 | FOS | activation |
| EDN1 | FOS | activation |
| HRAS | FOS | activation |
| AGTR1 | FOS | activation |
| EGF | FOS | activation |
| DNMT1 | FOS | activation |
| FYN | FOS | activation |
| CDK4 | FOS | inhibition |
| NRAS | FOS | activation |
| CCNB1 | FOS | activation |
| GDF15 | FOS | inhibition |
| IL5 | FOS | activation |
| ELK1 | FOS | inhibition |
| FOSL1 | FOS | activation |
| MAP2K6 | FOS | activation |
| IL17A | DEFB4A | activation |
| IL1A | DEFB4A | activation |
| IL6 | ALOX5 | activation |
| CDKN1A | ALOX5 | activation |
| EGR1 | ALOX5 | activation |
| CDH1 | HMGA2 | inhibition |
| SELL | ANXA1 | inhibition |
| HIF1A | GDF15 | activation |
| AKT1 | GDF15 | activation |
| TNF | GDF15 | activation |
| ELAVL1 | GDF15 | activation |
| FOS | GDF15 | inhibition |
| GDF15 | GDF15 | inhibition |
| MAP2K6 | GDF15 | activation |
| IL6 | CTSB | activation |
| HIF1A | SOX9 | activation |
| CDKN1A | SOX9 | activation |
| NOTCH1 | SOX9 | activation |
| CDH2 | SOX9 | activation |
| SHH | SOX9 | activation |
| SOX9 | SOX9 | activation |
| CTSD | CTSD | activation |
| IL6 | CXCL1 | activation |
| IL1A | CXCL1 | activation |
| MMP9 | SMARCA4 | activation |
| CDH1 | SMARCA4 | activation |
| MMP2 | SMARCA4 | activation |
| NFE2L2 | SMARCA4 | activation |
| XRCC6 | XRCC6 | inhibition |
| MMP3 | PLCG1 | activation |
| ESR1 | HMGA1 | activation |
| BCL2 | GLI1 | activation |
| SHH | GLI1 | activation |
| FOXM1 | GLI1 | activation |
| PTGS2 | RARB | inhibition |
| RARB | RARB | inhibition |
| STAT3 | IL5 | activation |
| ICAM1 | IL5 | activation |
| FOS | IL5 | activation |
| TNF | DCN | inhibition |
| TGFB1 | DCN | inhibition |
| CDKN1A | DCN | activation |
| IL10 | DCN | activation |
| EGF | DCN | inhibition |
| THBS1 | DCN | activation |
| TNF | IRF1 | inhibition |
| CDKN1A | IRF1 | activation |
| IL12B | IRF1 | activation |
| IL6 | CD163 | activation |
| IL10 | CD163 | activation |
| HMOX1 | CD163 | activation |
| ADAM10 | ADAM10 | inhibition |
| CDH1 | SNAI2 | inhibition |
| PEBP1 | PEBP1 | activation |
| CSF3 | S100A8 | activation |
| CDKN2A | CDK6 | activation |
| TGFB1 | SPHK1 | activation |
| CCL2 | SPHK1 | inhibition |
| IL1A | SPHK1 | activation |
| EGF | SPHK1 | activation |
| SHH | AXL | activation |
| PTGS2 | BDKRB2 | activation |
| BDKRB1 | BDKRB2 | activation |
| MYC | CSF3 | activation |
| ERBB2 | CSF3 | activation |
| MMP9 | CSF3 | activation |
| MMP1 | CSF3 | activation |
| JUN | CSF3 | activation |
| FASLG | CSF3 | inhibition |
| EGR1 | CSF3 | activation |
| S100A8 | CSF3 | activation |
| BIRC3 | CSF3 | activation |
| CXCL5 | CSF3 | activation |
| CD82 | CD82 | inhibition |
| CXCR4 | SELL | activation |
| FOXO1 | SELL | activation |
| ANXA1 | SELL | inhibition |
| IGF1 | BIRC3 | activation |
| SERPINE1 | BIRC3 | activation |
| CSF3 | BIRC3 | activation |
| MMP2 | CD9 | activation |
| CCND1 | SFRP1 | inhibition |
| CASP3 | SFRP1 | inhibition |
| SHH | SFRP1 | activation |
| CDK4 | SFRP1 | activation |
| AKT1 | PIM1 | activation |
| PIM1 | PIM1 | inhibition |
| HOXA9 | PIM1 | activation |
| PTGS2 | NCOR1 | inhibition |
| MMP1 | SFN | activation |
| IGF1 | MDM4 | activation |
| PTGS2 | MMP8 | activation |
| EDN1 | GATA2 | activation |
| ANGPT2 | GATA2 | activation |
| PAX6 | GATA2 | inhibition |
| GATA2 | GATA2 | activation |
| SATB1 | GATA2 | inhibition |
| ID2 | GATA2 | inhibition |
| BIRC7 | BIRC7 | activation |
| BCL2 | SATB1 | activation |
| GATA2 | SATB1 | inhibition |
| MMP1 | NR4A2 | inhibition |
| CCND1 | KLF5 | activation |
| STAT3 | KLF5 | activation |
| EDN1 | KLF5 | activation |
| ILK | KLF5 | activation |
| ICAM1 | CD80 | inhibition |
| TNF | BBC3 | activation |
| TNF | AQP3 | inhibition |
| MET | AQP3 | activation |
| PTEN | MAP2K4 | inhibition |
| PTGS2 | MAP2K4 | activation |
| PIK3CA | ISG15 | inhibition |
| PIK3CG | ISG15 | inhibition |
| CDKN2A | E2F3 | inhibition |
| PON1 | SREBF2 | activation |
| AKT1 | ELK1 | inhibition |
| CDKN1A | ELK1 | inhibition |
| EGR1 | ELK1 | inhibition |
| FOS | ELK1 | inhibition |
| HIF1A | HIPK2 | inhibition |
| ERBB2 | RECK | inhibition |
| HIF1A | RECK | inhibition |
| MMP9 | RECK | inhibition |
| TIMP2 | RECK | activation |
| MYC | ID2 | activation |
| CDH1 | ID2 | inhibition |
| TGFB1 | ID2 | activation |
| CDKN1A | ID2 | inhibition |
| MTOR | ID2 | activation |
| IGF1 | ID2 | activation |
| SDC1 | ID2 | activation |
| EGR1 | ID2 | activation |
| GATA2 | ID2 | inhibition |
| NOTCH1 | FOSL1 | activation |
| MMP1 | FOSL1 | activation |
| FOS | FOSL1 | activation |
| TNF | CXCL5 | activation |
| EGR1 | CXCL5 | activation |
| CSF3 | CXCL5 | activation |
| CXCR4 | DLL4 | inhibition |
| NOTCH1 | DLL4 | activation |
| CCND1 | HOXA9 | activation |
| IGF1R | HOXA9 | activation |
| PIM1 | HOXA9 | activation |
| PLAU | MAPKAPK2 | inhibition |
| IL10 | CCL18 | activation |
| DICER1 | SOX4 | activation |
| LRIG3 | NTN1 | inhibition |
| CCND1 | HK2 | inhibition |
| BDKRB2 | BDKRB1 | activation |
| JUN | CCNA1 | activation |
| CCND1 | BTG2 | inhibition |
| PTGS2 | GDF9 | activation |
| EGF | HSD3B2 | activation |
| PSEN1 | ETS2 | activation |
| THBS1 | RRM2 | inhibition |
| CCND1 | RALA | activation |
| RALA | RALA | inhibition |
| PTGS2 | MAP2K6 | activation |
| PLAU | MAP2K6 | activation |
| THBS1 | MAP2K6 | activation |
| FOS | MAP2K6 | activation |
| GDF15 | MAP2K6 | activation |
| TGFB1 | ADAM9 | activation |
| MMP2 | NNMT | activation |
| STAT3 | NNMT | activation |
| MYC | LRIG1 | inhibition |
| ERBB2 | LRIG1 | inhibition |
| ERBB2 | MGAT5 | activation |
| IL6 | SENP1 | activation |
| CCND1 | SENP1 | activation |
| CDKN1A | SENP1 | activation |
| AR | PA2G4 | inhibition |
| PA2G4 | PA2G4 | inhibition |
| CDKN1A | TBX2 | inhibition |
| GJA1 | TBX2 | inhibition |
| MYC | CHKA | inhibition |
| IL6 | REG3A | activation |
| IL1A | LRAT | inhibition |
| SLCO2B1 | SLCO2B1 | activation |
| CDKN2A | EIF5A2 | inhibition |
| IL6 | NNAT | activation |
| TNF | ST3GAL1 | activation |
| NTN1 | LRIG3 | inhibition |
| CCR2 | PCDH8 | inhibition |

## Supplemental table 7

Drug-protein interaction for treatment vector in Bladder Cancer

| Drug | Protein | quantified interaction |
| --- | --- | --- |
| Cisplatin | HIF1A | -1 |
| Cisplatin | AKT1 | 1 |
| Cisplatin | TNF | 1 |
| Cisplatin | IL6 | 1 |
| Cisplatin | CCND1 | -1 |
| Cisplatin | CDKN1A | 1 |
| Cisplatin | GSTP1 | 1 |
| Cisplatin | MMP2 | 1 |
| Cisplatin | PTGS2 | -1 |
| Cisplatin | MTOR | 1 |
| Cisplatin | HMOX1 | 1 |
| Cisplatin | ICAM1 | 1 |
| Cisplatin | MAPK14 | 1 |
| Cisplatin | RELA | 1 |
| Cisplatin | PARP1 | 1 |
| Cisplatin | ERCC1 | 1 |
| Cisplatin | MSH2 | 1 |
| Cisplatin | FASLG | 1 |
| Cisplatin | BCL2L1 | -1 |
| Cisplatin | GPX1 | -1 |
| Cisplatin | PPARA | -1 |
| Cisplatin | EGR1 | 1 |
| Cisplatin | YBX1 | 1 |
| Cisplatin | TRPV1 | 1 |
| Cisplatin | TNFRSF10A | 1 |
| Cisplatin | FOS | 1 |
| Cisplatin | GSTA1 | 1 |
| Cisplatin | MDM4 | -1 |
| Cisplatin | HIPK2 | 1 |
| Thiotepa | CASP3 | 1 |
| Mitomycin C | ABCB1 | -1 |
| Mitomycin C | CASP3 | 1 |
| Mitomycin C | MAPK14 | 1 |
| Mitomycin C | PARP1 | 1 |
| Mitomycin C | CD80 | -1 |
| Gemcitabine | TERT | -1 |
| Gemcitabine | AKT1 | -1 |
| Gemcitabine | NFKB1 | -1 |
| Gemcitabine | CDKN1A | -1 |
| Gemcitabine | HMOX1 | 1 |
| Gemcitabine | CASP3 | 1 |
| Gemcitabine | RELA | -1 |
| Gemcitabine | PARP1 | 1 |
| Gemcitabine | FOXM1 | -1 |
| Gemcitabine | SPHK1 | 1 |
| Gemcitabine | BIRC3 | 1 |
| Gemcitabine | BIRC2 | 1 |
| Gemcitabine | RRM2 | 1 |

## Supplemental table 8

Initial condition vector in Bladder Cancer.

| Gene | Expression |
| --- | --- |
| MYC | -1 |
| TP63 | 1 |
| FGFR3 | 0 |
| TERT | 0 |
| NAT2 | 0 |
| PSCA | 1 |
| SLC14A1 | 0 |
| TBX3 | 0 |
| CCNE1 | -1 |
| TP53 | 1 |
| BLCAP | 0 |
| TACC3 | 0 |
| EGFR | 0 |
| MTHFR | 0 |
| VEGFA | -1 |
| BRINP1 | 0 |
| ERBB2 | 1 |
| HIF1A | -1 |
| CYP1A2 | 0 |
| CDKN2A | -1 |
| AKT1 | 0 |
| APOE | 1 |
| MMP9 | -1 |
| TNF | 0 |
| GSTM1 | 0 |
| CDH1 | 0 |
| NFKB1 | 0 |
| AR | 0 |
| TGFB1 | 1 |
| GSTT1 | 0 |
| IL6 | -1 |
| CLPTM1L | 0 |
| CCND1 | 0 |
| CDKN1A | -1 |
| BIRC5 | 0 |
| GSTP1 | 1 |
| EZR | 1 |
| MMP2 | 0 |
| XRCC1 | 0 |
| TLR4 | 0 |
| NOS3 | 1 |
| STAT3 | 0 |
| UGT1A | 0 |
| PTEN | -1 |
| APOBEC3A | -1 |
| CXCL8 | -1 |
| MDM2 | 0 |
| UGT1A10 | 0 |
| ESR1 | -1 |
| PTGS2 | -1 |
| IL10 | 0 |
| ACE | 0 |
| CTNNB1 | 0 |
| COMT | 0 |
| BCL2 | -1 |
| UGT1A8 | 0 |
| ERCC2 | 1 |
| CD44 | 1 |
| MTOR | 0 |
| ABCB1 | -1 |
| PPARG | 0 |
| KRAS | -1 |
| JAG1 | 0 |
| CXCR4 | -1 |
| VDR | 1 |
| BRAF | -1 |
| CYP1A1 | 0 |
| SOD2 | -1 |
| TERC | 0 |
| IGF1 | -1 |
| OGG1 | 1 |
| CYP2D6 | 1 |
| FAS | -1 |
| RB1 | 0 |
| NQO1 | 0 |
| TMEM129 | 0 |
| APOA1 | 0 |
| MET | 0 |
| CCL2 | 0 |
| JAK2 | 0 |
| TNFSF10 | 0 |
| ITGB1 | -1 |
| ESR2 | 1 |
| MAPK1 | 1 |
| SERPINE1 | -1 |
| CTLA4 | -1 |
| IGF1R | 0 |
| EDN1 | 0 |
| NOS2 | 0 |
| IL4 | 0 |
| NOTCH1 | 1 |
| POU5F1 | 0 |
| AURKA | 0 |
| PON1 | 0 |
| HLA-A | 0 |
| CAV1 | 0 |
| MMP1 | -1 |
| BLACAT1 | 0 |
| XRCC3 | 0 |
| PIK3CA | 0 |
| HMOX1 | -1 |
| IL17A | 0 |
| MIR21 | -1 |
| MAPK3 | 0 |
| CXCL12 | 0 |
| CASP3 | 0 |
| XPC | 1 |
| APOB | 0 |
| ICAM1 | -1 |
| MUC1 | 0 |
| HRAS | 0 |
| GSK3B | 0 |
| CYP2C19 | 0 |
| RASSF1 | 0 |
| MAPK14 | 1 |
| IL18 | 0 |
| NFE2L2 | 0 |
| CASP8 | 0 |
| RELA | 0 |
| CDKN1B | -1 |
| ITGB3 | 0 |
| GJB2 | 0 |
| HLA-G | 0 |
| MLH1 | 0 |
| CYP2E1 | 1 |
| PARP1 | 0 |
| PRKCA | 0 |
| CDKAL1 | -1 |
| IL1RN | -1 |
| HGF | -1 |
| KDR | 0 |
| TWIST1 | -1 |
| AGTR1 | 0 |
| IL1A | -1 |
| EGF | 0 |
| BAX | 1 |
| APEX1 | 0 |
| LGALS3 | 0 |
| CD274 | 0 |
| JUN | -1 |
| APC | 1 |
| HMGB1 | 0 |
| ERCC1 | 0 |
| TP73 | 1 |
| MSH2 | 0 |
| ABCG2 | 0 |
| MIF | 0 |
| PSEN1 | 0 |
| EZH2 | 0 |
| CD14 | 0 |
| MIR34A | 0 |
| EP300 | 0 |
| MMP3 | 0 |
| PLAU | -1 |
| PTK2 | 1 |
| FASLG | 0 |
| MKI67 | 0 |
| IL13 | 0 |
| MAPK8 | 0 |
| CYP1B1 | 0 |
| LEPR | -1 |
| FGF2 | -1 |
| BSG | 1 |
| ILK | 1 |
| NPM1 | -1 |
| SERPINA1 | 0 |
| STAT1 | -1 |
| CLU | 1 |
| CHEK2 | 0 |
| BMI1 | 0 |
| SLC2A1 | 0 |
| VEGFC | -1 |
| IGF2 | 0 |
| TLR9 | 0 |
| MCL1 | -1 |
| RUNX3 | -1 |
| FLT1 | 0 |
| BCL2L1 | 1 |
| CCDC112 | 0 |
| FGFR2 | 1 |
| NAMPT | -1 |
| RUNX1 | -1 |
| CDH2 | 0 |
| SMAD4 | 0 |
| CDK1 | 0 |
| GPX1 | 0 |
| GJA1 | 0 |
| MIR145 | 0 |
| PLAUR | -1 |
| PLK1 | 0 |
| GRB2 | 1 |
| ERBB3 | 0 |
| IL12B | 0 |
| MMP7 | 0 |
| PDCD1 | 0 |
| PPARA | 0 |
| MIR155 | 0 |
| DNMT1 | -1 |
| SDC1 | 0 |
| PRKAR2B | 0 |
| LTA | 1 |
| CREB1 | 1 |
| APOC3 | 0 |
| SLC35E3 | 0 |
| PIK3R1 | 1 |
| VIM | 0 |
| PRKDC | 1 |
| TOP2A | 0 |
| THBS1 | -1 |
| PML | 1 |
| FHIT | 0 |
| MIR146A | 0 |
| KISS1 | 0 |
| SHH | 1 |
| CHI3L1 | 0 |
| NFKBIA | -1 |
| TGFBR2 | 1 |
| PDGFRA | 0 |
| CEBPA | 1 |
| FYN | -1 |
| GRN | 1 |
| IDO1 | 0 |
| DICER1 | 0 |
| IL15 | -1 |
| IL4R | 0 |
| MTR | 0 |
| SOX2 | 0 |
| BECN1 | 1 |
| FOXO1 | -1 |
| ID1 | 0 |
| CCR2 | -1 |
| MTDH | 0 |
| EGR1 | -1 |
| TIMP2 | 0 |
| MME | 0 |
| FOXM1 | 0 |
| MYH9 | 1 |
| ANGPT2 | 0 |
| HPSE | 0 |
| TGFBR1 | 0 |
| CDK4 | 0 |
| STK11 | 1 |
| PVT1 | 1 |
| NCOA3 | 0 |
| CALR | 1 |
| XRCC5 | 0 |
| INSIG1 | -1 |
| ERBB4 | -1 |
| NRAS | 0 |
| SLBP | 0 |
| H2AFX | 1 |
| LSP1 | 1 |
| PIK3CG | 0 |
| AATBC | 0 |
| CCNB1 | 0 |
| CA9 | 0 |
| S100A4 | 0 |
| CD34 | 0 |
| PAK1 | 1 |
| EPCAM | 0 |
| THBD | -1 |
| SERPINB5 | 0 |
| YBX1 | 0 |
| GATA3 | 0 |
| PAX6 | 0 |
| EPHX1 | 1 |
| TRPV1 | 0 |
| SNAI1 | 0 |
| ELAVL1 | 1 |
| CYP2A6 | 0 |
| S100A9 | 0 |
| TNFRSF10A | 0 |
| RPS6KB1 | 1 |
| IL23R | 0 |
| FOS | 0 |
| DEFB4A | 0 |
| ALOX5 | 1 |
| KLF4 | -1 |
| AURKB | 0 |
| SLCO1B1 | 0 |
| HMGA2 | 0 |
| ANXA1 | 0 |
| SULT1A1 | 0 |
| EIF4E | -1 |
| GDF15 | -1 |
| NR1I2 | 1 |
| CTSB | -1 |
| ROCK1 | 0 |
| WWOX | 0 |
| NAT1 | 0 |
| PPARD | 0 |
| COL1A2 | -1 |
| FASN | 1 |
| SERPINC1 | 0 |
| SOX9 | -1 |
| MRE11A | -1 |
| CTSD | 1 |
| ZEB1 | -1 |
| CXCL1 | 0 |
| SMARCA4 | 1 |
| XRCC6 | 0 |
| PLCG1 | 0 |
| PAG1 | 0 |
| HMGA1 | -1 |
| IL22 | 0 |
| CXCR2 | 1 |
| GLI1 | 0 |
| GC | -1 |
| PTCH1 | 1 |
| RARB | 0 |
| ANGPT1 | 0 |
| GSN | 1 |
| TSC1 | 1 |
| KPNA5 | 0 |
| POSTN | 0 |
| IFNA1 | 0 |
| IL5 | 0 |
| ANG | 0 |
| CTTN | 0 |
| COL18A1 | 0 |
| DIABLO | 0 |
| DCN | 0 |
| GSTA1 | 0 |
| IRF1 | -1 |
| CD163 | 0 |
| CEACAM1 | 0 |
| ADAM10 | 0 |
| SNAI2 | 0 |
| ERCC5 | 0 |
| PEBP1 | 1 |
| S100A8 | -1 |
| HTR5A | 0 |
| PXN | 0 |
| MIR221 | 0 |
| HDAC6 | 1 |
| IGFBP2 | 1 |
| NRP1 | -1 |
| EIF4EBP1 | 1 |
| CDK6 | 0 |
| STMN1 | -1 |
| CBX6 | 0 |
| MCF2L | -1 |
| PDPN | -1 |
| SPHK1 | 0 |
| ERCC4 | 0 |
| AXL | 0 |
| BAP1 | 0 |
| DAPK1 | -1 |
| TEK | 0 |
| BDKRB2 | -1 |
| CSF3 | 0 |
| XRCC4 | 0 |
| CD82 | 1 |
| SELL | -1 |
| BIRC3 | -1 |
| ACKR3 | -1 |
| H19 | 0 |
| CD9 | 0 |
| SFRP1 | 0 |
| KPNA2 | -1 |
| APOA2 | 0 |
| PIM1 | -1 |
| RBX1 | 0 |
| UHRF1 | 0 |
| SLC14A2 | 0 |
| NCOR1 | 0 |
| FOLH1 | 0 |
| SFN | -1 |
| MIR200B | 0 |
| CRK | 0 |
| IQGAP1 | 1 |
| IL27 | 0 |
| EDNRA | -1 |
| MIR126 | 0 |
| CHRNA5 | 0 |
| POR | 0 |
| MIR27A | 0 |
| CHRNA3 | 0 |
| CDH13 | 0 |
| MDM4 | 0 |
| MIR203A | 0 |
| ITGA3 | 1 |
| MMP8 | 0 |
| GATA2 | 1 |
| CGB3 | 0 |
| ERCC6 | 0 |
| BIRC7 | 0 |
| PAXIP1 | -1 |
| VCAN | -1 |
| MST1R | 0 |
| BIRC2 | 0 |
| TAGAP | 0 |
| SATB1 | 0 |
| HOTAIR | 0 |
| NR4A2 | -1 |
| KLF5 | 0 |
| MIR200C | 0 |
| ALOX12 | 0 |
| DMBT1 | 0 |
| CD80 | 0 |
| MIR143 | 0 |
| POU5F1B | 0 |
| BBC3 | 1 |
| ALCAM | 0 |
| AQP3 | 0 |
| HPGD | 0 |
| RHOC | 1 |
| ACTN4 | 0 |
| UGT2B7 | 0 |
| MUC5B | 0 |
| CXADR | 0 |
| JUP | 0 |
| SNCG | 1 |
| MAP2K4 | 0 |
| ISG15 | 0 |
| PAX5 | 0 |
| APOC2 | 0 |
| PLCE1 | 0 |
| E2F3 | 0 |
| DEK | 0 |
| CD74 | 0 |
| RGS2 | -1 |
| CRKL | 0 |
| AKR1C3 | 0 |
| PPP1CA | 0 |
| MIR196A2 | 0 |
| SREBF2 | 1 |
| MIR205 | 0 |
| KIF11 | 0 |
| KHDRBS1 | 0 |
| ELK1 | 0 |
| LAMC2 | 0 |
| SCD | 0 |
| CSF3R | 0 |
| EBAG9 | 0 |
| CUL3 | -1 |
| HIPK2 | 0 |
| DROSHA | -1 |
| CASP2 | 1 |
| RECK | 0 |
| TNFSF15 | 0 |
| SLCO1B3 | -1 |
| CDKN2B-AS1 | 0 |
| DEFA1 | 0 |
| MIR31 | 0 |
| DKK3 | 0 |
| OGT | 0 |
| IFNAR1 | 0 |
| MIR16-1 | 0 |
| UBE2C | 0 |
| FOXF2 | 0 |
| NDRG2 | 1 |
| MCM7 | 0 |
| ID2 | 0 |
| FOSL1 | -1 |
| MIR200A | 0 |
| CXCL5 | 0 |
| CDH3 | 0 |
| KIAA1524 | 0 |
| PPM1D | -1 |
| DLL4 | 0 |
| TGFBR3 | 0 |
| HOXA9 | 0 |
| MAPKAPK2 | -1 |
| MALAT1 | 0 |
| CCL18 | 0 |
| CTNNA1 | 0 |
| SOX4 | 1 |
| PEPD | 0 |
| ORM1 | 0 |
| WIF1 | 0 |
| NUMA1 | 1 |
| MIR125B1 | 0 |
| CDC25B | -1 |
| NTN1 | 0 |
| MSR1 | 1 |
| MIR499A | 0 |
| GSTO1 | 0 |
| MIR137 | 0 |
| HK2 | 0 |
| TDGF1 | 0 |
| UGT1A6 | 0 |
| MIR222 | 0 |
| KRT1 | 0 |
| KRT20 | -1 |
| UCA1 | 0 |
| HMMR | 0 |
| PTMA | 0 |
| BDKRB1 | 0 |
| CCNA1 | 0 |
| MDC1 | 0 |
| MYNN | 0 |
| MIR182 | 0 |
| MAPK11 | 0 |
| BTG2 | 0 |
| NRP2 | 1 |
| CHRNB4 | 0 |
| GDF9 | 0 |
| MIR141 | 0 |
| SLC15A1 | 0 |
| FMN1 | 0 |
| HSD3B2 | 0 |
| MIR218-1 | 0 |
| SUZ12 | 0 |
| FBLN1 | 0 |
| ETS2 | -1 |
| MIR10B | 0 |
| PKP2 | 0 |
| AS3MT | 0 |
| PCBP1 | 0 |
| CLIC1 | 0 |
| CLK3 | 0 |
| UGT1A7 | 0 |
| RRM2 | -1 |
| ARHGDIB | 0 |
| ZBTB7A | 1 |
| PELI2 | 0 |
| MIR214 | 0 |
| RALA | -1 |
| ID4 | 0 |
| SLC31A1 | 0 |
| MIR100 | 0 |
| MAP2K6 | 0 |
| PODXL | 0 |
| WDR5 | 0 |
| MIR9-1 | 0 |
| MIR1-1 | 0 |
| GNL3 | -1 |
| ADAM9 | 0 |
| EIF3A | 0 |
| IMPDH2 | 0 |
| CFAP57 | 0 |
| S100A11 | 1 |
| NNMT | -1 |
| LRIG1 | 0 |
| STAG2 | -1 |
| MIR29C | 0 |
| AKR1C2 | 0 |
| TBXAS1 | 0 |
| ESM1 | 0 |
| HAS2 | 0 |
| HSPA2 | 0 |
| CAV2 | -1 |
| MGAT5 | 0 |
| SENP1 | 0 |
| BCL2L12 | 0 |
| MAP2K7 | 0 |
| KDM6A | 0 |
| MIR451A | 0 |
| XAF1 | 0 |
| MIR19A | 0 |
| KDM5B | 0 |
| MIR195 | 0 |
| MAGEA3 | -1 |
| MIR133A1 | 0 |
| PA2G4 | 0 |
| IL20 | 0 |
| ARL6IP5 | 0 |
| RSPH3 | 0 |
| HYAL1 | 0 |
| EN2 | 0 |
| CSE1L | -1 |
| USP2 | 0 |
| TBX2 | 1 |
| LASP1 | 0 |
| MTSS1 | 1 |
| CHKA | 1 |
| RBBP7 | 0 |
| MEG3 | 1 |
| HAS1 | 0 |
| TMEFF2 | 0 |
| PCDH10 | 0 |
| MIR224 | 0 |
| CSMD1 | 0 |
| MIR96 | 0 |
| VEGFB | 1 |
| GGH | -1 |
| GSTZ1 | 1 |
| SLC7A11 | -1 |
| FZD4 | 0 |
| 4-Sep | 1 |
| BMX | 0 |
| AGO1 | -1 |
| GSTO2 | 0 |
| NUPR1 | 1 |
| RBP1 | 0 |
| REG3A | 0 |
| PBRM1 | 1 |
| LRAT | 0 |
| DEFA3 | 0 |
| TRIM24 | -1 |
| TRIM29 | 1 |
| MCM5 | -1 |
| FUBP1 | -1 |
| LTB4R2 | 0 |
| MAP2K3 | -1 |
| DAB2IP | 0 |
| MIR24-1 | 0 |
| RNH1 | 0 |
| MIR10A | 0 |
| SLCO2B1 | 0 |
| MIR99A | 0 |
| PBK | 0 |
| MIR18A | 0 |
| RAB25 | 0 |
| ADAM28 | -1 |
| MYCL | -1 |
| MAGEA4 | 0 |
| FANCL | 0 |
| MIR133B | 0 |
| GAS5 | 0 |
| MIR144 | 0 |
| MIR152 | 0 |
| MIR27B | 0 |
| HOXD10 | 0 |
| RALB | 0 |
| MIR193A | 0 |
| TPPP | 1 |
| MIR7-1 | 0 |
| PRSS3 | 0 |
| MIR192 | 0 |
| SMUG1 | 0 |
| CERS2 | 0 |
| MIR23B | 0 |
| MGEA5 | 0 |
| HEPACAM | 0 |
| PPM1A | 0 |
| CASP5 | 0 |
| ATPIF1 | 0 |
| PIWIL2 | 0 |
| DLGAP5 | 0 |
| ACTRT3 | 0 |
| SENP2 | 0 |
| GALNT1 | -1 |
| INPP4B | -1 |
| RHOBTB2 | 1 |
| UPK3A | 0 |
| HOXB5 | 1 |
| TLX3 | 0 |
| CYP4B1 | 0 |
| RPS4X | 0 |
| SRSF9 | 0 |
| MED19 | 0 |
| MIR449A | 0 |
| DHX35 | 0 |
| HMGN5 | 0 |
| CKS2 | -1 |
| EIF5A2 | 0 |
| RBM3 | 0 |
| PCDH17 | -1 |
| CARD10 | 0 |
| RIT1 | 0 |
| EOMES | 0 |
| UNC5B | 1 |
| IFNL2 | 0 |
| GLIPR1 | -1 |
| CREB3L1 | 0 |
| NNAT | 0 |
| CHD1L | -1 |
| ALDH1A2 | 1 |
| LRRC34 | 0 |
| TAGLN2 | 0 |
| ST3GAL1 | 0 |
| SAA4 | 0 |
| LRIG3 | 0 |
| DAPK2 | 0 |
| POU4F2 | -1 |
| IFN1@ | 0 |
| CCNE2 | -1 |
| CDH4 | 1 |
| UPK2 | 0 |
| MTUS1 | 1 |
| CTAG2 | 0 |
| ANXA10 | 0 |
| PFKFB4 | 0 |
| GNB4 | 0 |
| HMGB3 | 0 |
| MIR124-3 | 0 |
| MIR135A1 | 0 |
| FGFRL1 | 0 |
| SOCS7 | 0 |
| BAIAP2L1 | 0 |
| PHLPP2 | 0 |
| SKA1 | 0 |
| USP28 | 0 |
| PAQR3 | -1 |
| PCDH8 | 0 |
| ITIH5 | 0 |
| MIR30B | 0 |
| DMTF1 | 0 |
| FAM162B | 0 |
| HOXC9 | 0 |
| PFDN2 | 0 |
| MIR1-2 | 0 |
| NRG4 | 0 |
| MIR542 | 0 |
| MIR129-1 | 0 |
| MIR708 | 0 |
| PTPN21 | -1 |
| MAGEA9 | 0 |
| PMF1 | 1 |
| MIR20B | 0 |
| ZIC4 | 0 |
| MIR490 | 0 |
| GPR87 | 0 |
| L1TD1 | 0 |
| FUBP3 | 0 |
| ZAR1 | 0 |
| UNC5A | 0 |
| C20orf187 | 0 |
| LOC339593 | 0 |
| MIR7-3 | 0 |
| ZNF165 | -1 |
| MIR7-2 | 0 |
| SLC39A11 | 0 |
| PIGU | 0 |
| ALKBH8 | 0 |
| ZNF335 | 1 |
| PCAT1 | 0 |
| PRAC1 | 0 |
| PBOV1 | 0 |
| MIR135A2 | 0 |
| MIR517A | 0 |
| ZNF154 | 0 |
| CSTP1 | 0 |
| RALGAPA2 | 0 |
| CCNJ | 0 |
| MIR493 | 0 |
| ZDHHC11 | 0 |
| NEWENTRY | 0 |
| MIR576 | 0 |
| GHET1 | 0 |
| MIR320C1 | 0 |
| C16orf74 | 0 |
| ZEB2-AS1 | 0 |
| MIR1182 | 0 |
| MIR320C2 | 0 |
| COX2 | 0 |

## Supplemental table 9

*T*_d_ score for Bladder Cancer drugs

| Drug | Category | Score |
| --- | --- | --- |
| Cisplatin | D1 | 0.2 |
| Doxorubicin Hydrochloride | D1 | 0 |
| Thiotepa | D1 | 1 |
| Doxorubicin Hydrochloride | D2 | -0.2 |
| Gemcitabine | D2 | -0.09 |
| 4-methylpyrazole | D3 | 0 |
| 5-ASA | D3 | -1 |
| 5-azacytidine | D3 | -1 |
| 8-MOP | D3 | 1 |
| 9-cis-retinoic acid | D3 | 0 |
| abiraterone | D3 | 0 |
| acebutolol | D3 | 0 |
| acetaminophen | D3 | -1 |
| acitretin | D3 | -1 |
| adapalene | D3 | -1 |
| adenosine monophosphate | D3 | 1 |
| agomelatine | D3 | 1 |
| ajmaline | D3 | 0 |
| allylestrenol | D3 | 1 |
| amcinonide | D3 | -1 |
| AMD3100 | D3 | 1 |
| amiloride | D3 | -1 |
| aminoglutethimide | D3 | -1 |
| amiodarone | D3 | 1 |
| amitriptyline | D3 | 0 |
| amlodipine | D3 | 1 |
| amodiaquine | D3 | 0 |
| amrinone | D3 | -1 |
| amsacrine | D3 | 0 |
| anastrozole | D3 | 1 |
| arsenic trioxide | D3 | 1 |
| aspartame | D3 | 1 |
| aspirin | D3 | -1 |
| atomoxetine | D3 | 0 |
| atorvastatin | D3 | 0 |
| atropine | D3 | 1 |
| axitinib | D3 | -1 |
| azelastine | D3 | 0 |
| azilsartan medoxomil | D3 | 1 |
| azithromycin | D3 | 0 |
| benazepril | D3 | 1 |
| benzthiazide | D3 | -1 |
| betaxolol | D3 | 0 |
| bezafibrate | D3 | 1 |
| bicalutamide | D3 | -1 |
| biperiden | D3 | 0 |
| bortezomib | D3 | 0 |
| bosentan | D3 | 0 |
| bromfenac | D3 | -1 |
| bromocriptine | D3 | 1 |
| bumetanide | D3 | 1 |
| bupropion | D3 | 0 |
| cabozantinib | D3 | -1 |
| caffeine | D3 | 1 |
| candesartan | D3 | 1 |
| captopril | D3 | 0.333333 |
| carbinoxamine | D3 | 0 |
| carteolol | D3 | 0 |
| cefazolin | D3 | 1 |
| celecoxib | D3 | -1 |
| cerulenin | D3 | 0 |
| chloroquine | D3 | 0 |
| chlorpheniramine | D3 | 0 |
| chlorpromazine | D3 | 0 |
| cilazapril | D3 | 1 |
| cimetidine | D3 | 1 |
| cinacalcet | D3 | 0 |
| ciprofloxacin | D3 | 1 |
| cisplatin | D3 | -1 |
| citalopram | D3 | 1 |
| cladribine | D3 | 1 |
| clarithromycin | D3 | 1 |
| clemastine | D3 | 0 |
| clobetasol | D3 | 1 |
| clodronate | D3 | -1 |
| clofibrate | D3 | 1 |
| clomipramine | D3 | 0 |
| clotrimazole | D3 | 1 |
| clozapine | D3 | 1 |
| conjugated estrogens | D3 | 1 |
| corticotropin | D3 | 1 |
| cortisol | D3 | -1 |
| Cyclosporin A | D3 | 0 |
| cyproterone | D3 | -1 |
| dabrafenib | D3 | 0 |
| dalteparin | D3 | 0 |
| danazol | D3 | 0.333333 |
| darifenacin | D3 | 0 |
| decitabine | D3 | -1 |
| delavirdine | D3 | 1 |
| deprenyl | D3 | 1 |
| desipramine | D3 | 0 |
| desloratadine | D3 | 1 |
| desmopressin | D3 | 1 |
| desogestrel | D3 | 1 |
| dexamethasone | D3 | -1 |
| dexmedetomidine | D3 | 1 |
| dextromethorphan | D3 | 0 |
| diclofenac | D3 | 0.333333 |
| dicumarol | D3 | -1 |
| dienestrol | D3 | 1 |
| diethylcarbamazine | D3 | -1 |
| diflunisal | D3 | -1 |
| diltiazem | D3 | 0 |
| diphenhydramine | D3 | 0 |
| DMSO | D3 | 0 |
| dopamine | D3 | 1 |
| doxepin | D3 | 0 |
| doxorubicin | D3 | 0 |
| dronedarone | D3 | 0 |
| drospirenone | D3 | -1 |
| duloxetine | D3 | 0 |
| EDTA | D3 | 1 |
| efavirenz | D3 | 1 |
| eicosapentaenoic acid | D3 | 0 |
| eletriptan | D3 | 0 |
| eltrombopag | D3 | 1 |
| Enalapril | D3 | 1 |
| enoxacin | D3 | 1 |
| entacapone | D3 | 1 |
| epinastine | D3 | 0 |
| epinephrine | D3 | 1 |
| epirubicin | D3 | 0 |
| eprosartan | D3 | 1 |
| erlotinib | D3 | 0 |
| erythromycin | D3 | 1 |
| escitalopram | D3 | 0 |
| estradiol | D3 | 1 |
| estramustine | D3 | 1 |
| estriol | D3 | 1 |
| estrone | D3 | 1 |
| estropipate | D3 | 1 |
| ethinyloestradiol | D3 | 1 |
| ethynodiol diacetate | D3 | 1 |
| etodolac | D3 | -1 |
| etonogestrel | D3 | 1 |
| etoposide | D3 | 0 |
| etoricoxib | D3 | 0 |
| everolimus | D3 | 1 |
| felodipine | D3 | 0 |
| fenofibrate | D3 | 1 |
| fenoprofen | D3 | -1 |
| fexofenadine | D3 | 0 |
| fleroxacin | D3 | 0 |
| fludrocortisone | D3 | 1 |
| fluoxetine | D3 | 1 |
| fluphenazine | D3 | 1 |
| flurbiprofen | D3 | -1 |
| flutamide | D3 | 0 |
| fluvastatin | D3 | 1 |
| fluvoxamine | D3 | 1 |
| fondaparinux | D3 | 0 |
| fosinopril | D3 | 1 |
| gallium nitrate | D3 | 1 |
| gefitinib | D3 | 0 |
| gemfibrozil | D3 | 1 |
| glipizide | D3 | 0 |
| Glutathione | D3 | -1 |
| GR138950 | D3 | 1 |
| griseofulvin | D3 | -1 |
| halofantrine | D3 | 0 |
| heparin | D3 | 0 |
| histamine | D3 | 0 |
| Humalog | D3 | -1 |
| hydergin | D3 | 0 |
| hydrochlorothiazide | D3 | -1 |
| hydroflumethiazide | D3 | -1 |
| hydroxocobalamin | D3 | 0 |
| hydroxychloroquine | D3 | 0 |
| hydroxyurea | D3 | 0 |
| hydroxyzine | D3 | 0 |
| ibuprofen | D3 | -1 |
| Icatibant | D3 | -1 |
| idarubicin | D3 | 0 |
| imatinib | D3 | -1 |
| imipramine | D3 | 1 |
| indinavir | D3 | 0 |
| indomethacin | D3 | -1 |
| insulin detemir | D3 | -1 |
| irbesartan | D3 | 1 |
| isoniazid | D3 | 1 |
| isoproterenol | D3 | 1 |
| itraconazole | D3 | 0 |
| ketoconazole | D3 | 1 |
| ketoprofen | D3 | -1 |
| ketorolac | D3 | -1 |
| labetalol | D3 | 0 |
| lansoprazole | D3 | -1 |
| Lantus | D3 | -1 |
| lapatinib | D3 | -1 |
| lenalidomide | D3 | -1 |
| lercanidipine | D3 | 0 |
| letrozole | D3 | 0 |
| levofloxacin | D3 | 1 |
| levonorgestrel | D3 | 1 |
| lidocaine | D3 | 1 |
| lipoic acid | D3 | 0 |
| Lisinopril | D3 | 1 |
| lithium | D3 | -1 |
| L-methionine | D3 | 0 |
| lomefloxacin | D3 | 1 |
| lomustine | D3 | 0 |
| lopinavir | D3 | 1 |
| lornoxicam | D3 | -1 |
| losartan | D3 | 1 |
| lovastatin | D3 | 0 |
| lucanthone | D3 | 0 |
| lumefantrine | D3 | 0 |
| lumiracoxib | D3 | -1 |
| marimastat | D3 | -0.66667 |
| masoprocol | D3 | -1 |
| meclofenamate | D3 | -1 |
| medroxyprogesterone | D3 | 0 |
| mefenamic acid | D3 | -1 |
| mefloquine | D3 | 0 |
| melatonin | D3 | 0 |
| meloxicam | D3 | -1 |
| memantine | D3 | 0 |
| menadione | D3 | -1 |
| mepyramine | D3 | 0 |
| mestranol | D3 | 1 |
| methadone | D3 | 0 |
| methazolamide | D3 | 0 |
| methimazole | D3 | 1 |
| methylnaltrexone | D3 | 0 |
| methylphenidate | D3 | 0 |
| methyltestosterone | D3 | 1 |
| metoclopramide | D3 | 0 |
| metoprolol | D3 | 0 |
| metyrapone | D3 | 0 |
| mexiletine | D3 | 1 |
| mianserin | D3 | 0 |
| miconazole | D3 | 1 |
| midodrine | D3 | 0 |
| mifepristone | D3 | 0 |
| mimosine | D3 | -1 |
| minocycline | D3 | -1 |
| mirabegron | D3 | 0 |
| mitiglinide | D3 | 0 |
| mitoxantrone | D3 | 0 |
| moclobemide | D3 | 1 |
| moexipril | D3 | 1 |
| moxifloxacin | D3 | 0 |
| mycophenolate mofetil | D3 | 0 |
| mycophenolic acid | D3 | 0 |
| nabumetone | D3 | -1 |
| nafcillin | D3 | -1 |
| naloxone | D3 | 0 |
| naproxen | D3 | -1 |
| nateglinide | D3 | 0 |
| nelfinavir | D3 | 1 |
| neomycin | D3 | 1 |
| nepafenac | D3 | -1 |
| nevirapine | D3 | 1 |
| niacin | D3 | 0 |
| nicardipine | D3 | 0 |
| niclosamide | D3 | 1 |
| nicotine | D3 | -1 |
| nifedipine | D3 | 1 |
| niflumic acid | D3 | -1 |
| nilotinib | D3 | 0 |
| nilutamide | D3 | -1 |
| nilvadipine | D3 | 1 |
| nisoldipine | D3 | 1 |
| nitroprusside | D3 | 1 |
| norepinephrine | D3 | 1 |
| norfloxacin | D3 | 1 |
| norgestimate | D3 | 1 |
| nortriptyline | D3 | 0 |
| NovoLog | D3 | -1 |
| ofloxacin | D3 | 1 |
| olanzapine | D3 | 0 |
| olmesartan | D3 | 1 |
| omeprazole | D3 | 0 |
| ondansetron | D3 | 1 |
| orlistat | D3 | 0 |
| oxamniquine | D3 | 0 |
| oxandrolone | D3 | 1 |
| oxaprozin | D3 | -1 |
| oxprenolol | D3 | 0 |
| oxybutynin | D3 | 0 |
| paclitaxel | D3 | 1 |
| pantoprazole | D3 | -1 |
| paroxetine | D3 | 0 |
| pazopanib | D3 | -1 |
| pefloxacin | D3 | 1 |
| pentolinium | D3 | 0 |
| pentosan polysulfate | D3 | 0 |
| perhexiline | D3 | 0 |
| perindopril | D3 | 1 |
| perphenazine | D3 | 1 |
| phenobarbital | D3 | -1 |
| phenylbutazone | D3 | -1 |
| phenylephrine | D3 | -1 |
| Phosphoramidon | D3 | -1 |
| pilocarpine | D3 | 0 |
| pimecrolimus | D3 | -1 |
| pimozide | D3 | 0 |
| pioglitazone | D3 | 0 |
| pipotiazine | D3 | 0 |
| piroxicam | D3 | -1 |
| podophyllotoxin | D3 | 0 |
| pomalidomide | D3 | -1 |
| ponatinib | D3 | -1 |
| pranlukast | D3 | -1 |
| pravastatin | D3 | 0 |
| praziquantel | D3 | 0 |
| prednisolone | D3 | 0 |
| primaquine | D3 | 0 |
| primidone | D3 | -1 |
| progesterone | D3 | 1 |
| proguanil | D3 | 0 |
| promethazine | D3 | 0 |
| propafenone | D3 | 1 |
| propofol | D3 | 1 |
| propranolol | D3 | 1 |
| pyrimethamine | D3 | 0 |
| quetiapine | D3 | 0 |
| quinapril | D3 | 1 |
| quinidine | D3 | 1 |
| quinine | D3 | 0 |
| rabeprazole | D3 | -1 |
| raloxifene | D3 | 1 |
| ramipril | D3 | 1 |
| ranitidine | D3 | 1 |
| ranolazine | D3 | 0 |
| rapamycin | D3 | 1 |
| rasagiline | D3 | -1 |
| regorafenib | D3 | -1 |
| repaglinide | D3 | 0 |
| rescinnamine | D3 | 1 |
| retigabine | D3 | 0 |
| riboflavin | D3 | 0 |
| ridogrel | D3 | 0 |
| rifadin | D3 | -1 |
| rifapentine | D3 | 0 |
| rifaximin | D3 | 0 |
| rilpivirine | D3 | -1 |
| riluzole | D3 | 0 |
| risedronate | D3 | 1 |
| risperidone | D3 | 0 |
| ritonavir | D3 | -1 |
| rivastigmine | D3 | 0 |
| ropinirole | D3 | 1 |
| rosiglitazone | D3 | 1 |
| rotigotine | D3 | 1 |
| rutin | D3 | 0 |
| ruxolitinib | D3 | -1 |
| S-adenosylmethionine | D3 | -1 |
| salicylate | D3 | -1 |
| salsalate | D3 | -1 |
| saquinavir | D3 | 0 |
| secobarbital | D3 | -1 |
| sertraline | D3 | 0 |
| sildenafil | D3 | 0 |
| simvastatin | D3 | 0 |
| sorafenib | D3 | 0 |
| sparfloxacin | D3 | 0 |
| sparteine | D3 | 0 |
| Spirapril | D3 | 1 |
| spironolactone | D3 | -1 |
| streptozotocin | D3 | -1 |
| sulfaphenazole | D3 | 0 |
| sulfasalazine | D3 | -1 |
| sulindac | D3 | 0 |
| sulodexide | D3 | 0 |
| sunitinib | D3 | -1 |
| tamibarotene | D3 | -1 |
| tamoxifen | D3 | 0 |
| tapentadol | D3 | 0 |
| tasosartan | D3 | 1 |
| tazarotene | D3 | -1 |
| telithromycin | D3 | 0 |
| telmisartan | D3 | 1 |
| temsirolimus | D3 | 1 |
| teniposide | D3 | 0 |
| tenofovir | D3 | 1 |
| tenoxicam | D3 | -1 |
| terbinafine | D3 | 0 |
| testosterone | D3 | 1 |
| testosterone propionate | D3 | 1 |
| tetrahydrobiopterin | D3 | 1 |
| tetrahydrofolate | D3 | 0 |
| theophylline | D3 | 1 |
| thiabendazole | D3 | 1 |
| thioridazine | D3 | 1 |
| thiothixene | D3 | 0 |
| tiaprofenic acid | D3 | -1 |
| ticlopidine | D3 | 1 |
| tioconazole | D3 | 1 |
| tipranavir | D3 | 0 |
| tirofiban | D3 | 0 |
| tocainide | D3 | 1 |
| tolmetin | D3 | -1 |
| tramadol | D3 | 0 |
| trandolapril | D3 | 1 |
| tranylcypromine | D3 | 1 |
| treprostinil | D3 | 0 |
| triamcinolone | D3 | -1 |
| triamterene | D3 | 1 |
| trifluoperazine | D3 | 1 |
| triflusal | D3 | -1 |
| tripelennamine | D3 | 0 |
| triprolidine | D3 | 0 |
| troleandomycin | D3 | 0 |
| Trospium chloride (Sanctura | D3 | 0 |
| UDCA | D3 | 0 |
| valproate | D3 | 1 |
| valrubicin | D3 | 0 |
| valsartan | D3 | 1 |
| vandetanib | D3 | 0 |
| varenicline | D3 | 0 |
| vemurafenib | D3 | 1 |
| venlafaxine | D3 | 0 |
| verapamil | D3 | 0 |
| vinblastine | D3 | 0 |
| vinorelbine | D3 | 0 |
| vitamin B | D3 | 0 |
| vitamin D3 | D3 | 0 |
| vitamin E | D3 | 1 |
| vorinostat | D3 | 0 |
| yohimbine | D3 | 0 |
| zafirlukast | D3 | 1 |
| zidovudine | D3 | 0 |
| ziprasidone | D3 | 0 |
| zonisamide | D3 | -1 |

Fornari, F. A., J. K. Randolph, J. C. Yalowich, M. K. Ritke and D. A. Gewirtz (1994). "Interference by doxorubicin with DNA unwinding in MCF-7 breast tumor cells." Mol Pharmacol **45**(4): 649-656.

Lamb, J. (2007). "The Connectivity Map: a new tool for biomedical research." Nat Rev Cancer **7**(1): 54-60.

Subramanian, A., R. Narayan, S. M. Corsello, D. D. Peck, T. E. Natoli, X. Lu, J. Gould, J. F. Davis, A. A. Tubelli, J. K. Asiedu, D. L. Lahr, J. E. Hirschman, Z. Liu, M. Donahue, B. Julian, M. Khan, D. Wadden, I. C. Smith, D. Lam, A. Liberzon, C. Toder, M. Bagul, M. Orzechowski, O. M. Enache, F. Piccioni, S. A. Johnson, N. J. Lyons, A. H. Berger, A. F. Shamji, A. N. Brooks, A. Vrcic, C. Flynn, J. Rosains, D. Y. Takeda, R. Hu, D. Davison, J. Lamb, K. Ardlie, L. Hogstrom, P. Greenside, N. S. Gray, P. A. Clemons, S. Silver, X. Wu, W. N. Zhao, W. Read-Button, X. Wu, S. J. Haggarty, L. V. Ronco, J. S. Boehm, S. L. Schreiber, J. G. Doench, J. A. Bittker, D. E. Root, B. Wong and T. R. Golub (2017). "A Next Generation Connectivity Map: L1000 Platform and the First 1,000,000 Profiles." Cell **171**(6): 1437-1452 e1417.

Subramanian, A., P. Tamayo, V. K. Mootha, S. Mukherjee, B. L. Ebert, M. A. Gillette, A. Paulovich, S. L. Pomeroy, T. R. Golub, E. S. Lander and J. P. Mesirov (2005). "Gene set enrichment analysis: a knowledge-based approach for interpreting genome-wide expression profiles." Proc Natl Acad Sci U S A **102**(43): 15545-15550.
